# Uniformly shaped harmonization combines human transcriptomic data from different platforms while retaining their biological properties and differential gene expression patterns

**DOI:** 10.3389/fmolb.2023.1237129

**Published:** 2023-09-06

**Authors:** Nicolas Borisov, Victor Tkachev, Alexander Simonov, Maxim Sorokin, Ella Kim, Denis Kuzmin, Betul Karademir-Yilmaz, Anton Buzdin

**Affiliations:** ^1^ Omicsway Corp, Walnut, CA, United States; ^2^ Moscow Institute of Physics and Technology, Dolgoprudny, Russia; ^3^ Oncobox Ltd., Moscow, Russia; ^4^ World-Class Research Center “Digital Biodesign and Personalized Healthcare”, Sechenov First Moscow State Medical University, Moscow, Russia; ^5^ Clinic for Neurosurgery, Laboratory of Experimental Neurooncology, Johannes Gutenberg University Medical Centre, Mainz, Germany; ^6^ Department of Biochemistry, School of Medicine/Genetic and Metabolic Diseases Research and Investigation Center (GEMHAM) Marmara University, Istanbul, Türkiye; ^7^ Shemyakin-Ovchinnikov Institute of Bioorganic Chemistry, Moscow, Russia; ^8^ PathoBiology Group, European Organization for Research and Treatment of Cancer (EORTC), Brussels, Belgium

**Keywords:** gene expression, transcriptional profiles, RNA sequencing, microarray hybridization, data normalization and harmonization, platform bias, cancer transcriptomics, correlation analysis

## Abstract

**Introduction:** Co-normalization of RNA profiles obtained using different experimental platforms and protocols opens avenue for comprehensive comparison of relevant features like differentially expressed genes associated with disease. Currently, most of bioinformatic tools enable normalization in a flexible format that depends on the individual datasets under analysis. Thus, the output data of such normalizations will be poorly compatible with each other. Recently we proposed a new approach to gene expression data normalization termed Shambhala which returns harmonized data in a uniform shape, where every expression profile is transformed into a pre-defined universal format. We previously showed that following shambhalization of human RNA profiles, overall tissue-specific clustering features are strongly retained while platform-specific clustering is dramatically reduced.

**Methods:** Here, we tested Shambhala performance in retention of fold-change gene expression features and other functional characteristics of gene clusters such as pathway activation levels and predicted cancer drug activity scores.

**Results:** Using 6,793 cancer and 11,135 normal tissue gene expression profiles from the literature and experimental datasets, we applied twelve performance criteria for different versions of Shambhala and other methods of transcriptomic harmonization with flexible output data format. Such criteria dealt with the biological type classifiers, hierarchical clustering, correlation/regression properties, stability of drug efficiency scores, and data quality for using machine learning classifiers.

**Discussion:** Shambhala-2 harmonizer demonstrated the best results with the close to 1 correlation and linear regression coefficients for the comparison of training vs validation datasets and more than two times lesser instability for calculation of drug efficiency scores compared to other methods.

## 1 Introduction

Gene expression data are widely used in the fields of functional genomics and molecular medicine, e.g., in cancer research (∼350,000 PubMed papers found using search terms *gene expression* and *cancer* in April 2023). Two major approaches are used nowadays for large-scale transcriptional profiling: microarray hybridization (MH) of mRNA (Lashkari et al., 1997; Bednár, 2000; King and Sinha, 2001; Rew, 2001) and mRNA sequencing (RNAseq) (Nagalakshmi et al., 2008; Maher et al., 2009; Wang et al., 2009; Chu and Corey, 2012; Ingolia et al., 2012; Korir et al., 2015; Taylor et al., 2016). Both approaches utilize different rationales and can be further subdivided in several technological platforms. Consequently, the output data of MH and RNAseq are essentially platform-biased and require specific normalization/harmonization procedures in case of any inter-comparison may be needed.

Many aspects of such intra- and cross-platform normalization of MH gene expression profiles were studied in the recent 2 decades. Namely, the approaches were formulated for studying the incomparability of profiles obtained using different platforms (Shi et al., 2006; Chen et al., 2007; Liang, 2007), for normalization of their expression data (Bolstad et al., 2003; Benito et al., 2004; Jiang et al., 2004; Warnat et al., 2005; Johnson et al., 2007; Marron et al., 2007; Martinez et al., 2008; Shabalin et al., 2008; Xia et al., 2009; Huang et al., 2012), and for assessing quality of their co-normalization (Shi et al., 2006; Chen et al., 2007; Liang, 2007; Rudy and Valafar, 2011; Deshwar and Morris, 2014). In turn, the routine next-generation sequencing (NGS) of mRNA (RNAseq) has largely replaced MH in many applications and became the gold standard for transcriptomic profiling (Nagalakshmi et al., 2008; Maher et al., 2009; Wang et al., 2009; Chu and Corey, 2012; Ingolia et al., 2012; Korir et al., 2015; Taylor et al., 2016).

However, the emergence of NGS did not eliminate the problem of cross-platform bias, e.g., because different library preparation kits and different sequencing engines are in use, based on the different principles of signal detection (Borisov et al., 2022). In addition, in many applications comparisons of RNAseq and MH profiles were made that required cross-platform harmonization of data (Anders and Huber, 2010; Piccolo et al., 2013; Love et al., 2014; Maza, 2016; Thompson et al., 2016; Varet et al., 2016; Franks et al., 2018; Maleknia et al., 2020; Zhang et al., 2020; Fauteux et al., 2021; Tang et al., 2021; Huang et al., 2022, 292).

Most of such cross-platform harmonization/normalization methods return the results in a flexible format. As such, the shape of the output normalized gene expression profiles fully depends on the group of samples under normalization and can be poorly compatible with the results of another normalization involving different transcriptional profiles. Thus, a new normalization procedure is typically required for every comparison.

Furthermore, taking into account next-order variables utilizing gene expression data, such as molecular pathway activation levels (PALs) (Buzdin et al., 2014; Ozerov et al., 2016; Aliper et al., 2017; Borisov et al., 2017; Borisov et al., 2020a), drug efficiency scores (DES) (Poddubskaya et al., 2019; Tkachev et al., 2020b; Zolotovskaia M. et al., 2020), and machine learning (ML) models (Borisov et al., 2018; Borisov and Buzdin, 2019; Tkachev et al., 2019; Tkachev et al., 2020a; Borisov et al., 2020b; Borisov et al., 2021a; Borisov et al., 2021b), this flexibility may unpredictably complicate gene expression analyses due to probable inconsistence of quantitative characteristic gene expression features.

More recently, a new concept was formulated for the harmonization methods: conversion of a whole set of profiles into the shape of a pre-defined experimental platform, e.g., in the Training Distribution Machine (TDM) method (Thompson et al., 2016). According to this paradigm, the harmonized results should look as if they were obtained using a single pre-defined gene expression profiling platform.

We then introduced a new type of uniformly shaped cross-platform harmonizers that use several mathematical transforms (Borisov et al., 2019; 2022; Borisov and Buzdin, 2022). The first version of such software, Shambhala-1 (Borisov et al., 2019) used uniformly shaped harmonization that employed the piecewise-linear gene expression transformation method XPN (Shabalin et al., 2008; Rudy and Valafar, 2011). Later on, the XPN method was replaced in Shambhala by a more advanced piecewise-cubic method CuBlock (Junet et al., 2021), thus giving next version termed Shambhala-2 (Borisov et al., 2022).

Current versions of Shambhala utilize transformation of a fraction of ∼8,000 most strongly expressed human genes because their transcriptional activities can be assessed with the greatest precision compared to the low-expressed genes (Borisov et al., 2019). In our previous report (Borisov et al., 2022) we showed that Shambhala-2 returns transformed gene expression profiles that are clustered according to their biological origin rather than by their experimental platform. However, it remained unexplored whether this harmonization also retains differential gene expression features that can functionally characterize the samples under analysis.

Here, we tested Shambhala performance in retention of fold-change gene expression features and other functional characteristics of gene clusters such as pathway activation levels and predicted cancer drug activity scores. Using 6,793 cancer and 11,135 normal tissue gene expression profiles from the literature and experimental datasets, we applied twelve performance criteria for different versions of Shambhala and other methods of transcriptomic harmonization with flexible output data format. Such criteria dealt with the biological type classifiers, hierarchical clustering, correlation/regression properties, and stability of drug efficiency scores. We also assessed the quality of Shambhala output data for building both local and global machine learning expression-based classifiers of human cancer and normal tissue types. The piecewise-cubic Shambhala-2 harmonizer demonstrated the best results with the close to 1 correlation and linear regression coefficients for the comparison of training vs. validation datasets and more than two times lesser instability for calculation of drug efficiency scores compared to other methods.

## 2 Materials and methods

### 2.1 Study design

**FIGURE 1:**
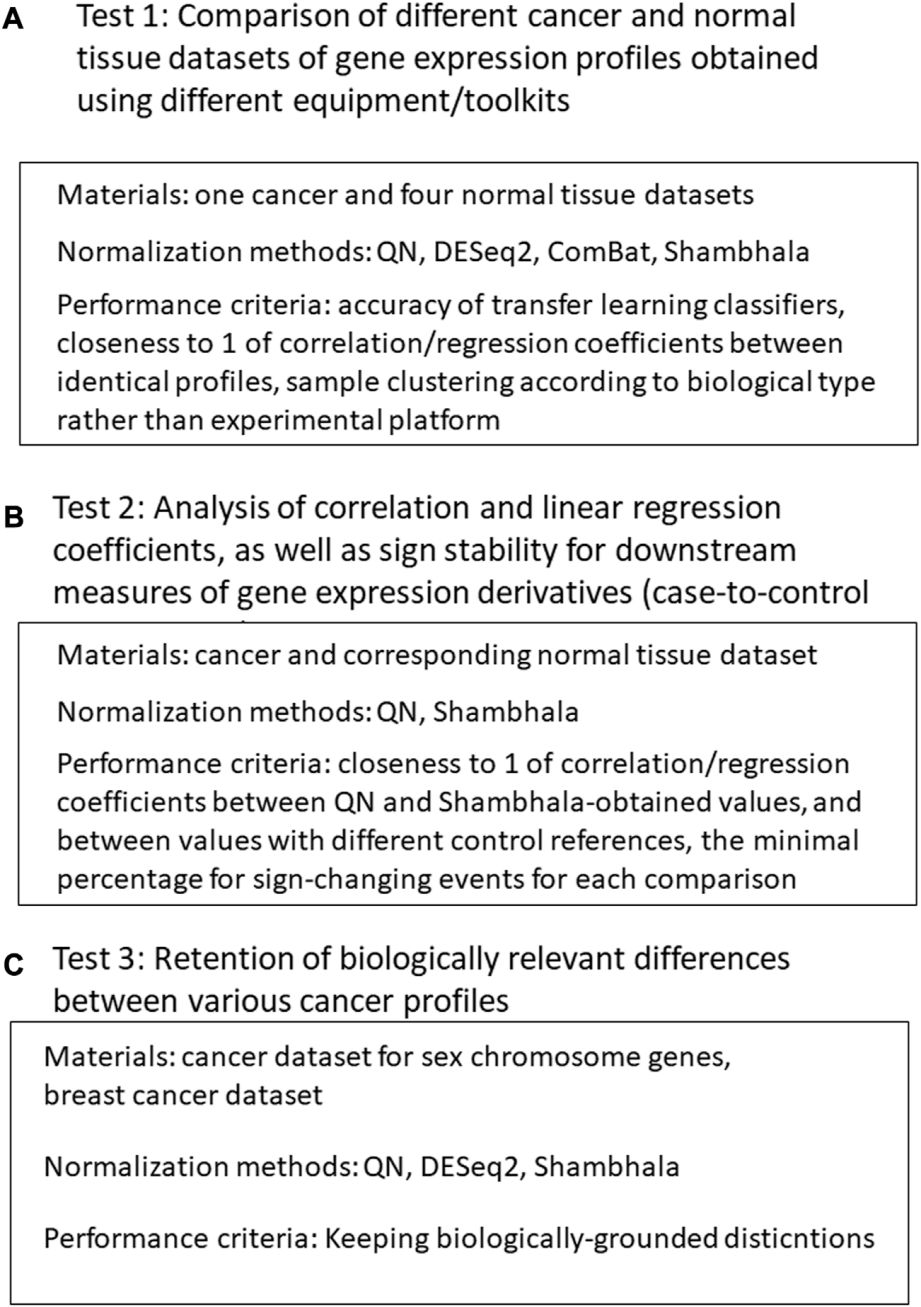
Study design. **(A)**: Comparison of different cancer and normal tissue datasets of gene expression profiles obtained using different equipment/toolkits. **(B)** Analysis of correlation and linear regression coefficients, as well as sign stability for downstream measures of gene expression derivatives. **(C)** Analysis for retention of biologically relevant differences between various cancer profiles after harmonization.

Our validation of the Shambhala method for uniformly-shaped harmonization included the following tests (Figure 1).

1) Comparison of different cancer and normal tissue datasets of gene expression profiles obtained using different equipment/protocols. The normalization methods included QN (Bolstad et al., 2003), DESeq2 (Love et al., 2014), Empirical Bayes a.k.a ComBat (Johnson et al., 2007; Lagani et al., 2016), and different modification of linear and cubic Shambhala. The quality control metrics included:
  - the accuracy of the transfer learning classifier, where the ML model is trained on profiles obtained in one batch and validated on profiles obtained in different batches and using different platforms/protocols;
  - clustering quality of expression profiles after harmonization: “good harmonization” means that such clustering corresponds to biological type of the sample rather than to batch or experimental platform;
  - correlation and linear regression coefficient between expression profiles for the same tissue type, obtained using different equipment/protocols: should be close to 1 for a good harmonization.
2) Analysis of correlation and linear regression coefficients, as well as sign stability for downstream measures of gene expression derivatives (case-no-control log-fold changes, pathway activation levels (PALs) (Aliper et al., 2017), and drug balanced efficiency score (BES) (Tkachev et al., 2020b). For a good harmonization, the correlation and linear regression coefficients should be close to 1, and the sign change rates should be as low as possible. The correlation, linear regression and sign stability tests were performed in the following conditions:
  - different linear and cubic Shambhala modes vs. QN;
  - LFC, PAL, and BES values calculated using Oncobox ANTE control sampling vs. GTEx control sampling.
3) Retention of biologically relevant differences between various types of profiles after harmonization (QN, DESeq2, and multiple Shambhala modifications):
  - differences between the male and female individual samples for the genes located on sex chromosomes,
  - differences between cancer expression profiles in hormone-dependent, HER2-positive, and triple-negative breast cancer patients.

### 2.2 Gene expression datasets

We curated six gene expression datasets for cancer and corresponding normal transcriptomic profiles, namely, The Cancer Genome Atlas (TCGA) (Tomczak et al., 2015)—both for cancer (i) and normal (ii) tissues; Gene-Tissue Expression Consortium (GTEx) (GTEx Consortium, 2013; The GTEx Consortium et al., 2015)—normal samples obtained using both RNAseq (iii) and MH (iv); (v) Oncobox Atlas of Normal Tissue Expression, ANTE, (Suntsova et al., 2019), and (vi) Oncobox experimental collection of human cancer expression profiles. Among these six gene expression datasets, five were obtained by RNA sequencing (with platforms Illumina HiSeq 2000 and 3000, and one–using expression microarray platform Affymetrix Human Gene 1.1 ST Array (Table 1). Four RNA expression profiling protocols were used to obtain the above six datasets. Two datasets represented cancer samples, and four were obtained for normal human tissues (Table 1). All sequencing data used here represented full-length RNA sequencing.

**TABLE 1:** Gene expression sample types and group sizes used for the tissue type classifier comparisons.

Sample type	Cancer TCGA	Normal TCGA illumina HiSeq 2000; protocol by (Tomczak et al., 2015)	Normal GTEx illumina HiSeq 2000; protocol by (The GTEx Consortium et al., 2015)	Normal GTEx	Normal ANTE illumina HiSeq 3000; protocol by (Suntsova et al., 2019)	Cancer oncobox illumina HiSeq 3000; protocol by (Suntsova et al., 2019)
	Illumina HiSeq 2000			Affymetrix human gene 1.1 ST array		
	Protocol by (Tomczak et al., 2015)			Protocol by (GTEx Consortium, 2013)		
Acute myeloid leukemia/bone marrow	358	–	–	–	–	20
Adrenocortical carcinoma/adrenal gland	79	3	190	–	5	4
Normal bladder	–	19	11	–	4	–
Breast cancer/normal breast	1142	114	290	–	5	79
Cervical cancer (all types)/normal cervix (uterus)	306	38	129	–	8	18
Cholangiocarcinoma	36					5
CNS glioblastoma/normal CNS: brain	169	5	1671	206	5	47
Normal CNS: other	–	–	414	54	5	–
Colorectal cancer/normal colorectal intestine	–	51	507	–	6	107
Normal esophagus	–	13	1021	–	7	–
Normal fat (adipose tissue)	–	–	797	33	–	–
Normal heart	–	–	600	62	–	–
Hepatocellular carcinoma/normal liver	374	50	175	–	6	7
Lung cancer (all types)/normal lung	1046	110	427	–	7	62
Melanoma/normal skin	472	1	1203	65	6	10
Normal ovary	–	–	133	–	4	–
Pancreatic adenocarcinoma/normal pancreas	179	4	248	–	5	17
Normal peripheral blood	–	–	537	–	6	–
Normal prostate	–	52	152	1	6	–
Renal cell carcinoma/normal kidney	837	129	45	–	6	32
Soft tissue sarcoma, non-rhabdomyosarcoma/normal skeletal muscle	263	-	564	86	6	11
	–	–	137	–	5	–
Stomach adenocarcinoma/normal stomach	456	37	262	–	7	29
Thyroid cancer/normal thyroid gland	513	59	446	69	6	108
Uterine corpus endometrial carcinoma	558	–	–	–	–	7
Total number of samples	6,230	685	9,959	576	115	563

### 2.3 Shambhala harmonization of gene expression profiles

Harmonization of datasets using Shambhala-1 and Shambhala-2 methods was done as previously described (Borisov et al., 2022), Figure 2. Both methods perform harmonization of each gene expression profile independently in the initial raw (*R*) dataset. The procedure relies on two preselected auxiliary datasets: the calibration (*P*) and reference definitive (*Q*) datasets. Every single profile is taken from the *R*-dataset, and quantile-normalize (Bolstad et al., 2003) with the *P*-dataset, to form the transformed dataset *P*ʹ. Then *P*ʹ-dataset is normalized using the XPN (Shabalin et al., 2008) or CuBlock (Junet et al., 2021) protocols for Shambhala-1 and -2 methods, respectively, to produce the double transformed dataset *P*ʺ. From the dataset *P*ʺ, the finally harmonized individual profile is obtained; the harmonization procedure is repeated for every different profile in the dataset *R*. The whole procedure converts the initial dataset *R* into the harmonized dataset *H*.

**FIGURE 2:**
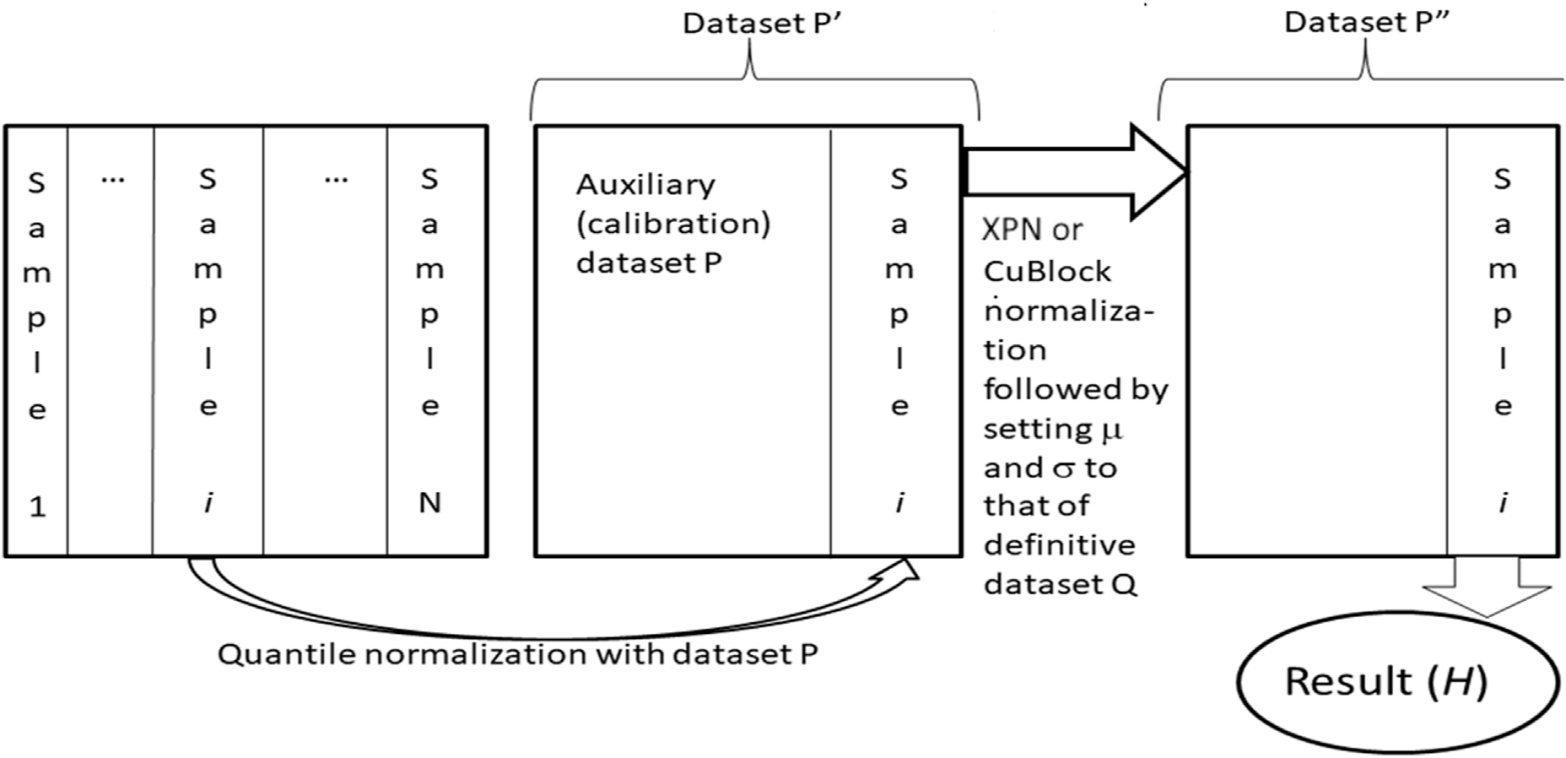
Shambhala-1/2 approach (Borisov et al., 2019; 2022; Borisov and Buzdin, 2022) to uniformly shaped harmonization of gene expression data. Gene expression profiles for samples (1, … ,*N*), e.g., obtained using different experimental platforms are taken one by one, separately merged, and quantile-normalized with an auxiliary calibration dataset *P*. The resulting dataset *P*ʹ is then transformed into the format of the reference definitive dataset *Q*, which results in dataset *P*ʺ. The latter contains the finally transformed profile of initial sample *i*, which is considered harmonized (*H*). The profiles of all other samples are harmonized one by one using the same algorithm.

We used three alternative rescaling modes for Shambhala-2 method. The *P*-based rescaling (Borisov et al., 2022) utilizes simple translation and multiplication of the log-expression level (*LE*_*g*_ʺ) for each gene *g* in the dataset *H* as follows: *LE*_*g*_ = μ_*gQ*_ *+ LE*_*g*_ʺ·σ_*gQ*_, where μ_*gQ*_ and + σ_*gQ*_ are mean value and standard deviation, respectively, for log-expression level of gene *g* in the *Q-*dataset. Second mode termed *Q-*based rescaling*,* utilizes setting the mean value, and the standard deviation, for the log-expression levels of each gene in the dataset *H*, to the corresponding levels of the dataset *Q*, equal to μ_*gQ*_ and σ_*gQ*_, respectively: *LE*_*g*_ = μ_*gQ*_ *+* (*LE*_*g*_ʺ − μ_*gH*_)·σ_*gQ*_ */*σ_*gH*_. The third mode termed *R-*based rescaling, has the mean value, and the standard deviation, for the log-expression levels of each gene *g* that are used in the dataset *H* to the corresponding levels of the dataset *R*, equal to μ_*gR*_ and σ_*gR*_, respectively: *LE*_*g*_ = μ_*gR*_ *+* (*LE*_*g*_ʺ *−* μ_*gH*_)·σ_*gR*_ */*σ_*gH*_.

As the auxiliary datasets *P* and *Q*, we used the dataset *P*_0_ (obtained using the MH platform Affymetrix Human Genome U133A 2.0 Array) and *Q*_0_ (obtained using the NGS platform GTEx Ilumina HiSeq 2000), respectively. Among other tested *P*- and *Q*-datasets, the datasets *P*_0_ and *Q*_0_ showed the best results in our previous studies (Borisov et al., 2022).

### 2.4 Classification of tissue type using harmonized gene expression profiles

The following six methods were used for harmonization of gene expression data.

1) Quantile normalization (QN) (Bolstad et al., 2003), implemented as the *normalize.quantiles* method from the *preprocessCore* R package, available at https://github.com/bmbolstad/preprocessCore;
2) DESeq2 normalization (Love et al., 2014; Maza, 2016; Varet et al., 2016), implemented as the *estimateSizeFactors* method from the *DESeq2* R package, available at https://bioconductor.org/packages/release/bioc/html/DESeq2.html. Although DESeq2 can be used for differential gene analysis, it can also be applied for agnostic normalization of the NGS data, if all profiles are considered as belonging to one batch.
3) ComBat, or Empirical Bayes, designed for artificial elimination of the batch effect between two gene expression datasets (Johnson et al., 2007; Lagani et al., 2016), available with the *sva* package at Bioconductor code repository: https://bioconductor.org/packages/release/bioc/html/sva.html;
4) Shambhala-1, or linear Shambhala (Borisov et al., 2019). The code for Shambhala-1 (R package *HARMONY*) is available at https://github.com/oncobox-admin/harmony;
5) Shambhala-2 (Borisov et al., 2022) with *P*-based rescaling (Sh2PBR) of the output data, written as R code that calls a MATLAB function, available at https://github.com/BorisovNM/Shambhala2;
6) Shambhala-2 with *Q*-based rescaling (Sh2QBR) of the output data;
7) Shambhala-2 with *R*-based rescaling (Sh2RBR) of the output data.

To avoid overtraining, we applied the transfer learning approach to the sample tissue type classifiers. After harmonization of gene expression profiles, we used one group of samples as the training dataset, and another group as the validation dataset (Table 1). The number of classes, *i.e.*, biological sample types, was fifteen for the cancer type classifier, and varied from 8 to 20 (depending on the selection of training and validation datasets) for normal tissue classifiers. All the classifiers used the Euclidean feature space of log-expression of each gene (for the kNN approach) and of 20 principal components (for the SVM approach), applying three machine learning methods: (i) 11 nearest neighbors (11nn); (ii) first nearest neighbor (1nn); (iii) linear support vector machine (SVM). The SVM calculations were performed using the *e1071*R package (Liu and Wang, 2015) with the C++ library *libsvm* (Chang and Lin, 2011).

### 2.5 Clustering quality assessment of harmonized expression profiles

We used Watermelon Multisection (WM) method to quantitatively assess the quality of clustering for the expression profiles under analysis according to (Zolotovskaia M. A. et al., 2020; Borisov and Buzdin, 2022; Borisov et al., 2022). This method returns a specific metric for the assessment of an entropy-based quality of clustering on dendrograms according to known predefined classes. WM can evaluate performance of hierarchical clustering relative to a trait of interest, e.g., known tissue type in our case. When moving from the root of the dendrogram to its distal branches, one can measure information gain (IG) at each node of the dendrogram. In WM, the overall process of gradual information gain at each node is referred to as the observed information gain trajectory. Shortly, the WM metric is the normalized difference between the observed IG, theoretically maximal IG that corresponds to the fastest separation of predefined classes into the distinct branches, and null IG_0_ trajectory that describes the worst (totally random) distribution of predefined classes on the dendrogram (Zolotovskaia M. A. et al., 2020; Borisov et al., 2022). Therefore, 0 < WM < 1, and the higher value means better class separation on the dendrogram. High-quality harmonization is expected to result in clustering according to the tissue type, but not according to the experimental platform or other technical factors. Thus, the ratio $R=\frac{{WM}_{S}}{{WM}_{P}}$ of WM-metrics according to biological sample classes (*WM*_*S*_) and experimental platform classes (*WM*_*P*_) may be used for evaluating harmonization quality: the higher *R* means better harmonization quality (Borisov and Buzdin, 2022; Borisov et al., 2022).

WM metric calculation code was implemented in R, available at https://gitlab.com/oncobox/cluster-analysis.

We used the following protocol for WM metric evaluation (Borisov et al., 2022). For each harmonizing dataset, we randomly selected five samples for each known combination of tissue type and experimental profiling platform and then calculated the WM metrics for such a selection. Each selection was randomized and repeated 25 times according to (Borisov et al., 2022).

### 2.6 Correlation/regression analysis for median gene expression vectors

For each pair of training and validation datasets, we calculated the median log-expression levels for each gene and each sample type. Let us call the median log-expression level vectors for a certain biological type as ***v***_1_ and ***v***_2_, for the training and validation datasets, respectively. For each of ***v***_1_ vs. ***v***_2_ pairs, we calculated the Spearman correlation and linear regression coefficient (*k*). The value *k* was the geometric mean over the values *k*_1_ and *k*_2_. Here *k*_1_ and *k*_2_, are the linear regression coefficients with and without the offset item *b*, in the regression models: ***v***_2_ = *k*_1_ · ***v***_1_ + *b*, and ***v***_2_ = *k*_2_ · ***v***_1_, respectively. If *k*_1_·*k*_2_ < 0, then we set the resulting *k* value to zero.

### 2.7 Calculation of cancer-to-normal log-fold change, pathway activation level, and anticancer drug efficiency score values

Cancer-to-normal log-fold change (LFC) of gene expression, pathway activation levels (PALs), and balanced efficiency score of anticancer targeted drugs (BES) were calculated according to (Borisov et al., 2020a; Tkachev et al., 2020b). Cancer and normal tissue samples included in the analysis are listed in Table 2. Note that BES values are calculated using not only drug target genes, but also on the basis of PAL values, which calculation in turn requires nearly 8,000 genes that survive the Shambhala harmonization.

**TABLE 2:** Number of cancer and normal tissue gene expression profiles used for the functional tests of harmonization methods.

Cancer type	Cancer samples	Corresponding normal tissue samples	
	Oncobox RNAseq; protocol by (Suntsova et al., 2019)	ANTE RNAseq; protocol by (Suntsova et al., 2019)	GTEx RNAseq protocol by (The GTEx Consortium et al., 2015)
Adrenocortical carcinoma	4	5	190
Breast cancer	83	5	290
Carcinosarcoma	1	6	111
Cervical cancer (all types)	18	4	18
CNS glioblastoma	47	5	1671
CNS other tumors	28	5	414
Colorectal cancer	107	6	507
Esophageal carcinoma	1	7	1021
Hepatocellular carcinoma	7	6	175
Lung cancer (all types)	56	7	427
Melanoma	9	6	1,203
Ovarian cancer	34	4	133
Pancreatic adenocarcinoma	17	5	248
Prostate adenocarcinoma	2	6	152
Renal cell carcinoma	32	6	45
Skin carcinoma	6	6	1,203
Soft tissue sarcoma, non-rhabdomyosarcoma	6	6	111
Stomach adenocarcinoma	29	7	262
Thyroid cancer	111	6	446
Uterine corpus endometrial carcinoma	7	6	111
Total	605	114	8,738

### 2.8 Correlation, regression, and stability analysis for BES values

Shambhala applicability for BES calculations was tested as follows. We performed the correlation and linear regression analysis for two comparisons of BES vectors ***v***_1_ vs. ***v***_2_.

1) BES values calculated with normalization of cancer and corresponding ANTE normal samples (Suntsova et al., 2019) using QN^1^ (regarded as vector ***v***_1_) vs. using different modes of Shambhala: Sh1, Sh2PBR, Sh2QBR, or Sh2RBR (regarded as vector ***v***_2_);
2) BES values calculated with ANTE normal samples (regarded as vector ***v***_1_) vs. with GTEx normal samples (GTEx Consortium, 2013) (regarded as vector ***v***_2_).

For each comparison, we calculated the correlation and linear regression coefficient (*k*), similarly to the way that we applied to median log-expression values between similar biological types in the training and validation datasets.

Additionally, for these two comparisons, we calculated the percentage of sign-changed BES values as a function of the width (*w*) of a significance threshold around zero. If for the *i-*th component (corresponding to the drug *i*) of the vector ***v***_1_, *v*_1*i*_ < − *w*/2, and, simultaneously, *v*_*2i*_ > *w*/2, or *vice versa*, then the *i-*th component is considered sign-changing for the comparison of ***v***_1_ vs. ***v***_2_. The resulting percentage of sign-changed values is the ratio of the number of sign-changed components and of the total number of components.

Since different modes of Shambhala harmonization could affect the absolute values of LFC, PAL and BES, we calculated the percentage of the sign-changed BES values using two modes.

1) For the BES values without correction, marked “as is”;
2) For the BES values divided by the corresponding linear regression coefficient *k*, marked as “divided by *k*.”

## 3 Results

In this study, we tried to characterize the ability of Shambhala approach to provide tissue specific clustering of the harmonized gene expression profiles, and at the same time to retain their characteristic differential gene expression patterns. These points were assessed after co-harmonization of gene expression profiles obtained using two different experimental platforms and four library preparation protocols (Table 1).

### 3.1 Differential clustering of human normal and cancer expression profiles

We first investigated the ability of Shambhala to support tissue specific clustering of harmonized expression profiles in comparison with the other, non-uniformly shaped harmonization methods. We took human tissue gene expression datasets from the Gene-Tissue Expression (GTEx) repository (GTEx Consortium, 2013; The GTEx Consortium et al., 2015), The Cancer Genome Atlas (TCGA) database (Tomczak et al., 2015), Atlas of Normal Tissue Expression (ANTE) collection of expression profiles (Suntsova et al., 2019), and from the Oncobox experimental collection of cancer tissue RNA sequencing profiles. The samples were obtained using the versions of NGS platform Illumina: HiSeq 2000 for the TCGA and GTEx RNA sequencing data, and HiSeq 3000 for the ANTE and Oncobox cancer data, and the microarray hybridization (MH) platform Affymetrix Human Gene 1.1 ST Array for the GTEx MH data. Taken together, they represented 37 human tissue types including 15 cancer and 22 normal tissue types. Four different gene library preparation protocols were used for obtaining these datasets (Table 1), one common for the ANTE and Oncobox data; one common for the normal and cancer TCGA data, and specific protocols for the GTEx RNA sequencing, and MH data (Table 1).

To compare performance, we did harmonization procedure by using the following alternative methods: (i) Quantile normalization, QN, a gold standard for normalization of the MH gene expression data (Bolstad et al., 2003); (ii) DESeq2, a gold standard for normalization of the RNAseq gene expression data (Love et al., 2014; Maza, 2016; Varet et al., 2016); (iii) ComBat, specially developed for batch effect elimination (Johnson et al., 2007; Lagani et al., 2016); (iv) Shambhala-1, uniformly shaped harmonization method with the XPN gene expression transformation module (Borisov et al., 2019), Sh1; (v) Shambhala-2 (Borisov et al., 2022), uniformly shaped harmonization method with CuBlock gene expression transformation module and *P*-based rescaling (Sh2PBR); (vi) Shambhala-2 with the *Q*-based rescaling (Sh2QBR); (vii) Shambhala-2 with the *R*-based rescaling (Sh2RBR).

The *P*-, *Q*-, and *R*-rescaling modes for Shambhala-2 differ by different mean and standard deviation values, which are applied to set the log-expression levels after the harmonization procedure. Note that all modes of Shambhala reduced the number of genes after harmonization to ∼8,000 most strongly expressed “reaper” human genes (Borisov et al., 2019), Table 3. The genes in the harmonized output are formed by the intersection of genes in three datasets: the raw (*R*), and two auxiliaries (*P* and *Q*). The best results, in terms of stressing the biological origin of the profiles and banning the artifacts generated by platform/protocol-specific bias, were obtained for the *P*-dataset Affymetrix Human Genome U133A 2.0 Array (dataset *P*_0_) with 8,385 genes (Borisov et al., 2022). This MH platform is often used for routine transcriptomic profiling. Thus, using this *P*-dataset acts fairly similarly to the explicit expression filtering, and increases the signal-to-noise ratio, thereby highlighting the biological origin of the samples. For the sake of comparability between different normalization methods, we made all QN, DESeq2, and ComBat calculations with this set of highly expressed ∼8,000 genes, which help determine the biological origin of the profile.

**TABLE 3:** Overview of tissue type transfer learning classifiers.

Test ID	Training dataset	Validation dataset	Number of tissue types	Number of genes after Shambhala harmonization
1	TCGA cancer	Oncobox cancer	15	8,214
2	GTEx normal, RNAseq	TCGA normal	15	8,174
3	GTEx normal, RNAseq	ANTE normal	20	8,174
4	TCGA normal	GTEx normal, RNAseq	15	8,174
5	TCGA normal	ANTE normal	10	8,174
6	GTEx normal, RNAseq	GTEx normal, MH	8	7,862

We then applied the following machine learning (ML) methods to build tissue type classifiers for the harmonized profiles: (i) 11 nearest neighbors; (ii) first nearest neighbor; (iii) linear support vector machine (SVM). We performed 6 ML tests, one with cancer samples, and five with normal samples. In such tests, training and validation groups of samples were taken from different initial datasets (Table 3).

For each transfer learning test, and each sample type in the classifier, we calculated the accuracy, *i.e.*, percentage of correct tissue type predictions in the validation dataset.

**FIGURE 3:**
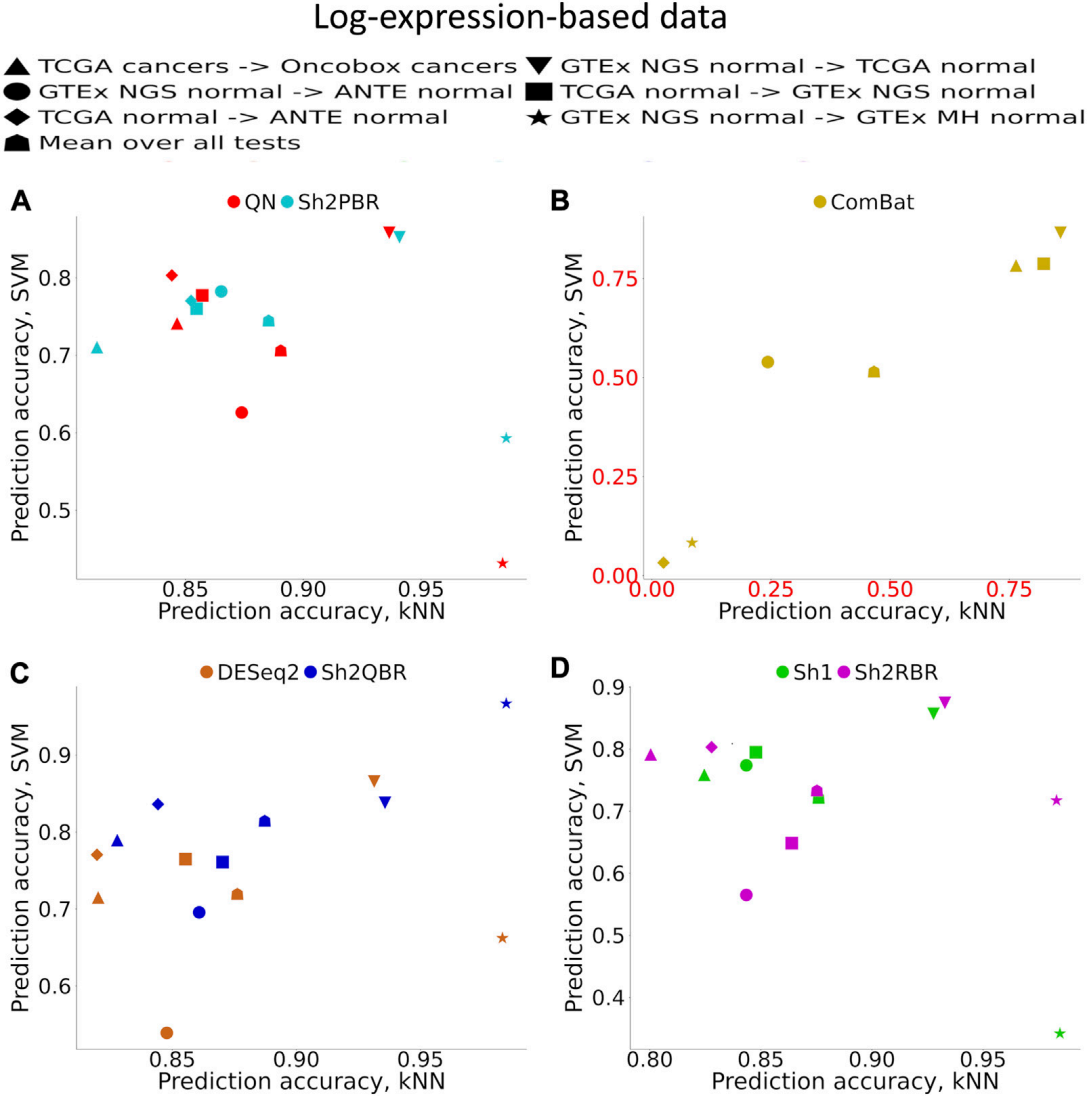
Total tissue type prediction accuracies calculated for local (kNN) and global (SVM) ML methods for different training and validation datasets. **(A)** QN, Sh2PBR, **(B)** ComBat, **(C)** DESeq2. Sh2QBR, **(D)** Sh1, Sh2RBR.

**FIGURE 4:**
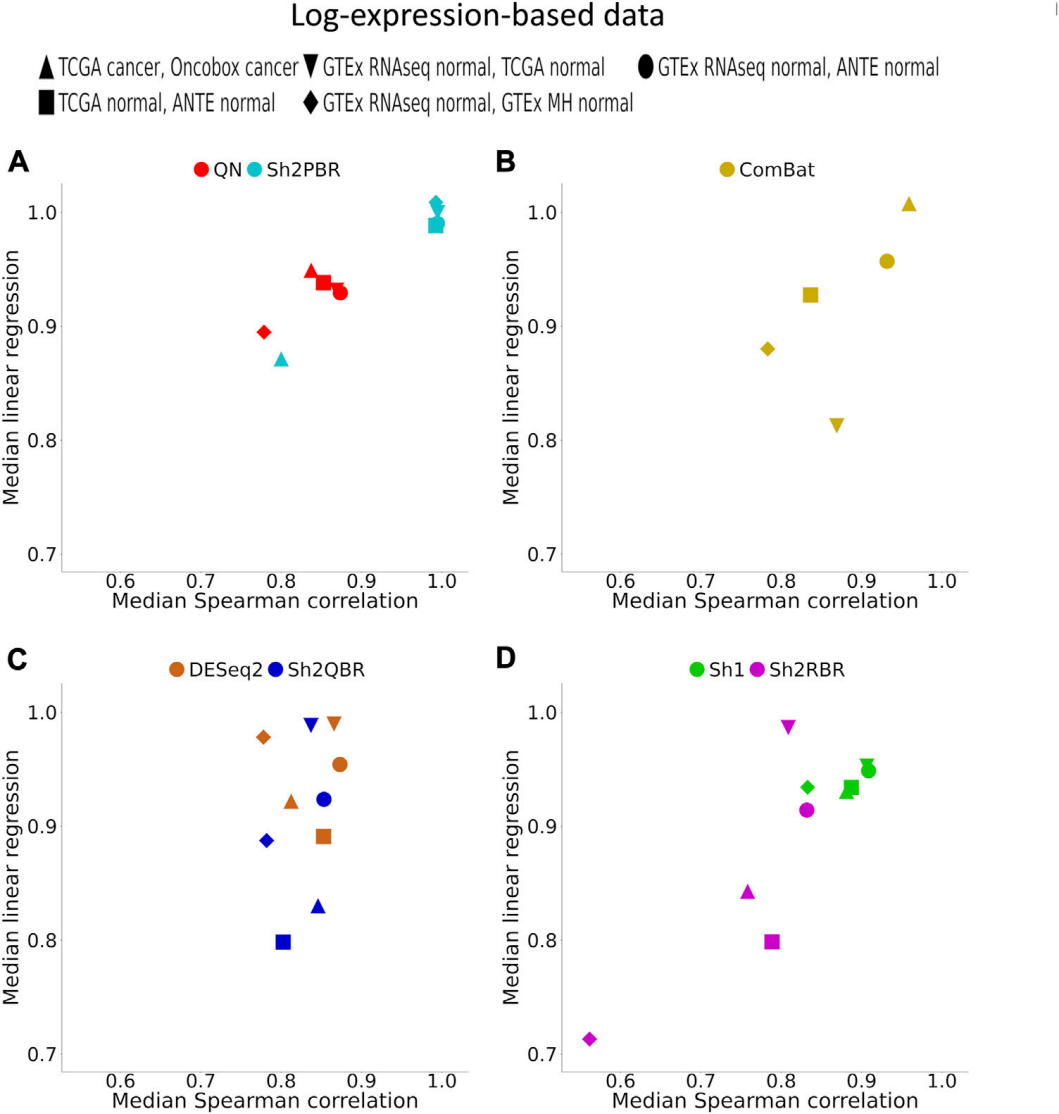
Median (over all possible tissue types) Spearman correlation and linear regression coefficients between identical tissue types from training and validation datasets. **(A)** QN, Sh2PBR, **(B)** ComBat, **(C)** DESeq2. Sh2QBR, **(D)** Sh1, Sh2RBR.

Supplementary Material S1 (Supplementary Figures S1–S6) demonstrates accuracy trends for predicting tissue types. Importantly, uniform output Shambhala method showed comparable performance with the gold standard flexible output methods QN and DESeq2, for both local (kNN-based) and global (SVM-based) classifiers (Figures 3A, C, D; Figure 4). Note that QN, DESeq2, and ComBat methods were also applied to the ∼8,000 genes, which survived Shambhala harmonization. Note also that the ComBat method designed for the batch affect removal, artificially makes two or more datasets under analysis to look similar, but mixes up the profiles with the different biological origin obtained using the same platform, which results in poor ML performance (Figures 3B, 4).

In addition, bigger number of samples in the *validation* dataset generally corresponded to greater prediction accuracy, although this trend is rather vague (Supplementary Material S1). From the Supplementary Figures S3A–C it looks like a number of samples higher than approximately 30 is required for all three classifiers tested to get a high accuracy, with a few exceptions. The reason for this phenomenon that can be seen in Supplementary Figure S1 is unknown to the authors, since the validation dataset is not used for ML model construction, and the number of cases in the validation dataset theoretically should not affect the expected accuracy of prediction. Interestingly, correlation of the prediction accuracy with the number of samples in the *training* dataset was even lower (data not shown).

Furthermore, Shambhala harmonization helped to restore distant order in the correct grouping of biologically similar samples (Table 4; Supplementary Material S2; Supplementary Figures S1, S2 to Supplementary Figures S2–S5). We assessed elimination of the platform/protocol-specific bias after harmonization using statistical method *Watermelon Multisection* (WM) (Zolotovskaia M. A. et al., 2020; Borisov and Buzdin, 2022; Borisov et al., 2022). WM enables tracking the entropy loss/information gain at each node of the clustering dendrogram when moving in the direction from the root to the distal branches, thus giving WM metric for a given dendrogram. It can assess the quality of sample clustering according to known predefined groups (in our case, tissue types). The higher is WM metric, the better is the clustering according to known tissue types, and *vice versa*.

**TABLE 4:** Median *R* values for WM-based quality metrics of different harmonization experiments.

Merged datasets	QN	DESeq2	ComBat	Sh1	Sh2PBR	Sh2QBR	Sh2RBR
TCGA cancer, Oncobox cancer	0.60	0.50	1.45	0.65	0.63	0.57	0.55
GTEx RNAseq normal, TCGA normal	0.88	0.84	1.42	1.17	1.50	1.26	0.93
GTEx RNAseq normal, ANTE normal	0.79	0.68	0.50	0.98	1.24	0.79	0.72
TCGA normal, ANTE normal	0.85	0.77	2.19	1.00	1.31	0.84	0.83
GTEx RNAseq normal, GTEx MH normal	0.82	0.77	6.29	0.92	1.24	1.01	0.75

We, therefore, calculated WM metrics for the harmonized tissue samples in two major settings: *WM*_*P*_ for the classes corresponding to experimental platforms/protocols (*e.g.*, TCGA-RNAseq, Oncobox-RNAseq, etc.), and *WM*_*S*_ for the classes corresponding to tissue types. Thus, the ratio *R* $=\frac{{WM}_{S}}{{WM}_{P}}$ may serve as the measure for harmonization quality. The higher is *R*, the better is clustering according to tissue type in relation to platform-specific bias, and *vice versa*.

In our analysis, the WM metric showed that the ComBat method had the best performance for platform bias elimination for most ML trials with cancer and normal human tissues, except for merging GTEx and TCGA normal RNAseq profiles, and merging GTEx and ANTE normal RNAseq profiles (Table 4; Supplementary Figures S2–S6 to Supplementary Figures S2–S10 in Supplementary Material S2). However, this elimination of the batch effect is only apparent, and does not result in proper clustering of the same sample types. The high values of *R* for WM metrics are provided by low values of *WM*_*P*_, rather than the high values of *WM*_*S*_, and profile clustering after ComBat is not done according to the tissue type (Supplementary Figures S2–S6 to Supplementary Figures S2–S10). Thus, according to our findings, ComBat method did not demonstrate an overall superior output as it blurs the similarity between the profiles of the same biological type, even when obtained using one experimental platform.

In addition, Sh2PBR harmonization mode also showed the optimal performance in the correlation-regression analysis of profiles for the same tissue samples. Having averaged the log-expression levels for each gene over all samples of certain type in the training and validation datasets, we arrived at the expression level vectors ***v***_1_ and ***v***_2_, respectively, for each tissue type. The distribution of Spearman correlation and linear regression coefficients between the corresponding ***v***_1_ and ***v***_2_ vectors are shown on Figure 4 and Supplementary Material S3.

An ideal method for cross-platform harmonization should result in as similar as possible transcriptomic profiles for the same samples or the same tissue types, even when obtained using different platforms or protocols. Consequently, the correlation and linear regression coefficients between the ***v***_1_ and ***v***_2_ vectors should be as close as possible to 1 (note that the linear regression coefficients may be also lower or greater than 1). We found that Sh2PBR method results in the correlation and linear regression coefficients very close to 1, thus being the method of choice for harmonization of human normal transcriptomic profiles (Figure 4 and Supplementary Material S3).

In brief, we may summarize that.

- In transfer learning tests, when the ML models were trained on gene expression profiles obtained using one experimental platform and validated on profiles obtained using another platform, the Shambhala modes showed performance better or comparable to QN and DESeq2, and better than ComBat;
- In terms of correlation/linear regression coefficients, the Sh2PBR mode showed the results maximally close to 1;
- The ComBat method, which is designed to eliminate the batch effect intentionally, showed the least performance in the tests with one cancer and four normal tissue datasets.

### 3.2 Correlation, regression, and sign-change analysis of cancer drug balanced efficiency score (BES) after application of different methods of harmonization

The universal harmonization of gene expression data is of interest not only for proper classification of biosamples. It has also important clinical implications. For example, bioinformatic platform Oncobox is designed for personalized prediction of cancer drug activities using cancer and normal gene expression profiles (Poddubskaya et al., 2019; Borisov et al., 2020a; Tkachev et al., 2020b; Zolotovskaia et al., 2022). It evaluates differential gene expression (reflected by log-fold change (LFC) values) and identifies altered molecular pathways (reflected by pathway activation levels, PALs) which may serve as the molecular targets for cancer drugs, thus giving balanced efficiency score (BES) for every drug under analysis. Other possible gene expression-based methods of drug efficiency scoring should be also mentioned in this context (Lazar et al., 2015; Solomon et al., 2022). However, finding proper tumor-matching normal tissues is frequently challenging and cannot accommodate for statistically correct differential gene analysis. It is, therefore, important to compare experimental cancer expression with the normal tissue datasets, e.g., published as the part of GTEx, TCGA, and ANTE projects. Such a comparison may require data harmonization, provided that different equipment, reagents and protocols could be employed for obtaining different datasets.

**FIGURE 5:**
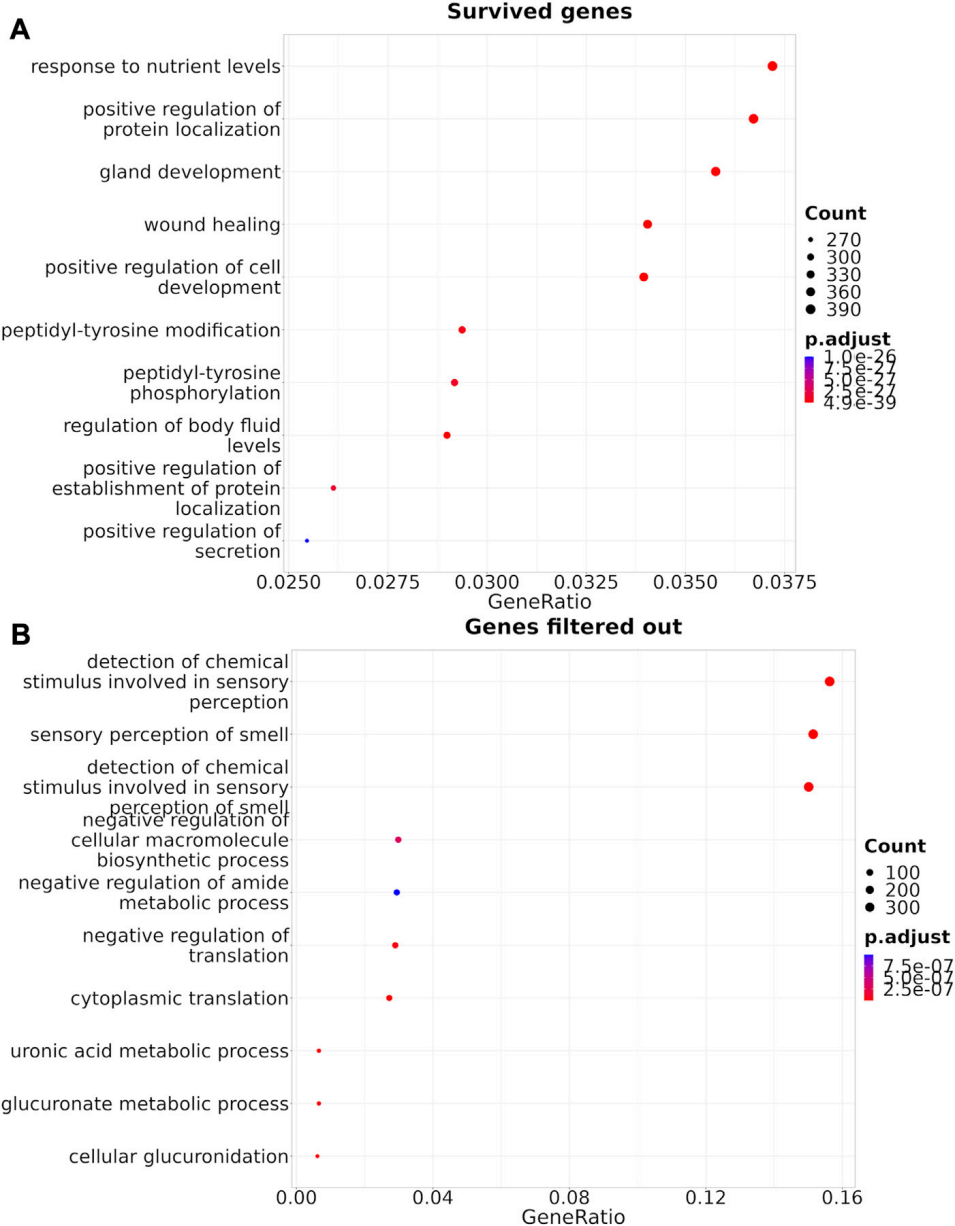
Gene Ontology terms enrichment analysis (Yu et al., 2012) for the genes that survived Shambhala harmonization **(A)**, or that were removed by filtering **(B)**.

The Shambhala harmonization has filtered away approximately 60% of protein-coding genes with the lowest expression (Table 3, Supplementary Material S4). However, among all 167 drugs in the Oncobox database, 129 (77%) retained all target genes after harmonization (Table 5). The enrichment analysis of Gene Ontology terms for the survived vs. filtered out genes (Figure 5) shows that the majority of survived genes govern important physiological process, including cell cycle, whereas the majority of rejected genes are related to sensory/olfactory mechanism, and only a minor part of neglected genes deal with the mitosis and cancer. Note also that important genes distinguishing between cancer subtypes, e.g., for breast cancer, like ERBB2 (HER2), ESR1 (ER), and two forms of membrane component of PR, PGRMC1 and PGRMC2, survived shambhalization.

**TABLE 5:** Genes survived after Shambhala harmonization which are included in the molecular pathway and drug target databases (Zolotovskaia et al., 2022) used in this study.

Database	Number of genes	Number of survived genes	Percentage of survived genes	Number of pathways	Number of pathways with >75% of genes survived, or number of drugs with all targets survived	Percentage of pathways with >75% of genes survived, or percentage of drugs with all targets survived
Balanced (Zolotovskaia et al., 2022) TCGA 826 1.4	5,484	4,212	77	328	328	100
Biocarta (Nishimura, 2001) 1.123	1,082	964	89	337	337	100
Metabolism (Zolotovskaia et al., 2022) 1.123	1,038	796	77	319	319	100
NCI (Schaefer et al., 2009)1.123	2,214	1,894	86	775	775	100
Qiagen (Zolotovskaia et al., 2022) 1.123	2,493	2,039	82	380	380	100
Reactome (Croft et al., 2014) 1.123	6,105	4,471	73	945	2	0.21
Drug targets (Tkachev et al., 2020b) 4.2	163	146	90	167	129	77

Thus, we assessed the influence of Shambhala harmonization on the BES values for these 129 targeted cancer drugs, for totally 605 experimental RNA sequencing samples of 20 cancer types (Table 2). We then analyzed performance of four Shambhala modifications (Sh1, Sh2PBR, Sh2QBR, and Sh2RBR) in a series of tests for correlation, regression, and sign-change assessments.

**FIGURE 6:**
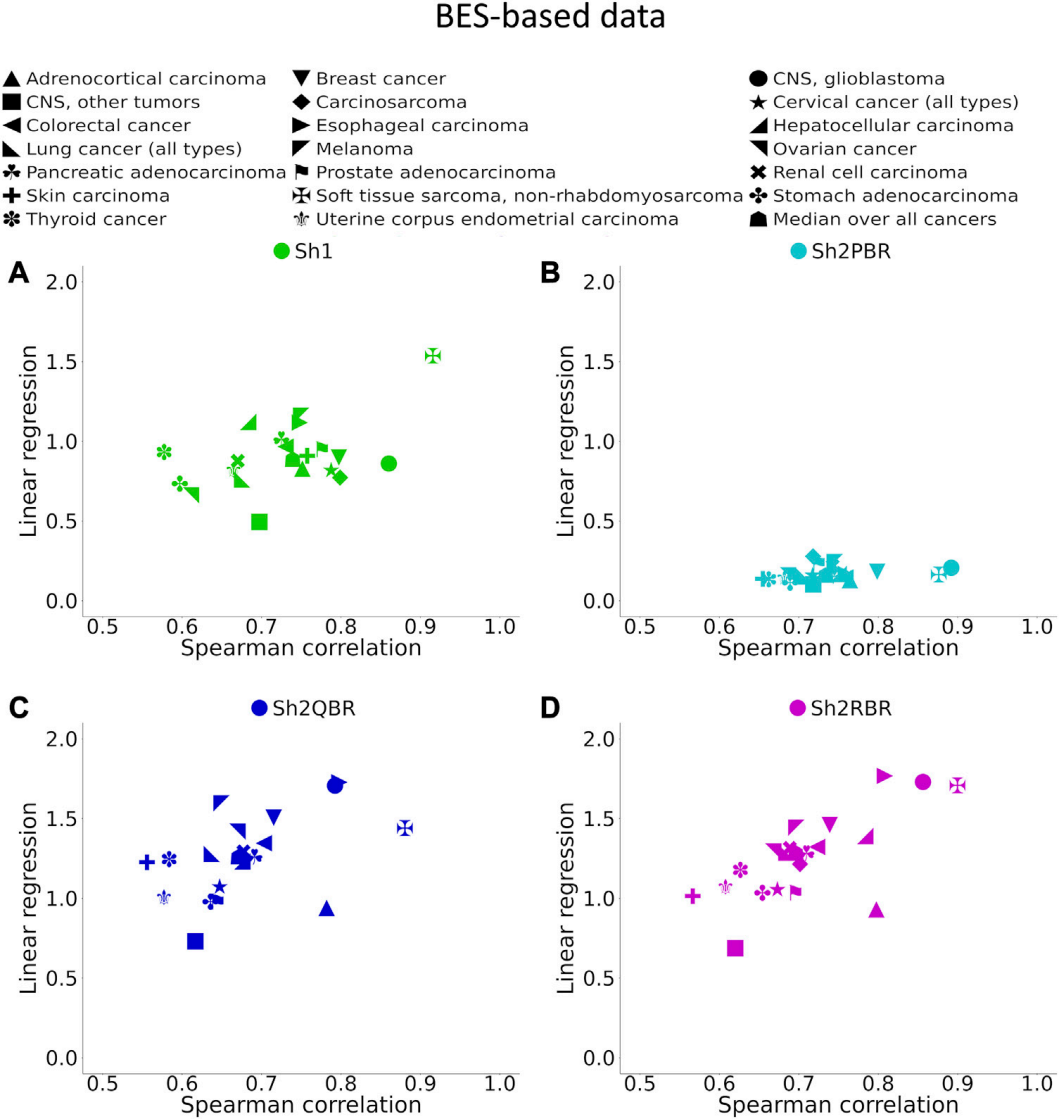
Spearman correlation and linear regression coefficients between BES values for QN vs. Shambhala comparison of 20 cancer types and 129 targeted cancer drugs. **(A)** QN, Sh2PBR, **(B)** ComBat, **(C)** DESeq2. Sh2QBR, **(D)** Sh1, Sh2RBR.

**FIGURE 7:**
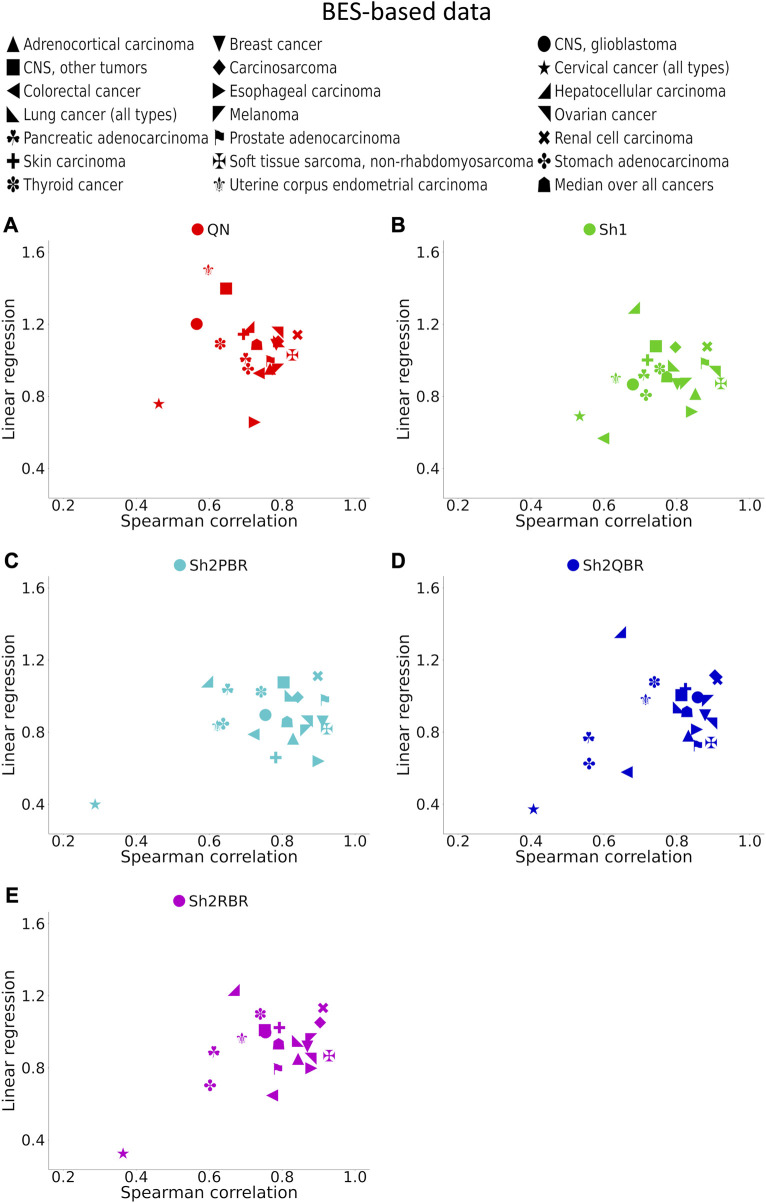
Spearman correlation and linear regression coefficients between the corresponding BES values for the same cancer drugs calculated with ANTE vs. GTEx normal references for 20 cancer types and 129 targeted cancer drugs. **(A)** QN, **(B)** Sh1, **(C)** Sh2PBR, **(D)** Sh2QBR, **(E)** Sh2RBR.

(i) first, comparison of BES values (***v***_1_), which were obtained using QN for Oncobox cancer and ANTE normal gene expression profiles, which is a routinely used protocol for BES calculation in clinical use (Tkachev et al., 2020b), vs. those (***v***_2_) which were obtained after Shambhala harmonization of cancer and normal profiles (Figure 6, Supplementary Figures S1–S6)
(ii) second, comparison of BES values (***v***_1_), which were obtained using ANTE normal gene expression profiles (Suntsova et al., 2019), vs. those (***v***_2_) obtained with the corresponding GTEx (GTEx Consortium, 2013) normal profiles (Figure 7, Supplementary Figures S2–S6).

We found that the BES values for QN and all modes of Shambhala harmonization were strongly correlated in all cancer types under analysis (Figure 6, Supplementary Figures S1–S6). It can be expected that ideal data harmonization will keep stable the cancer-to-normal LFC, and subsequent PAL and BES magnitudes, compared to the gold standard normalization methods such as QN or DESeq2. In other words, this should set the linear regression coefficient *k* close to 1 for the QN vs. Shambhala comparison. However, although this ideal figure was more or less true for the Sh1 mode, the best mode in terms of correlation coefficient, Sh2PBR, at the same time exhibited the worst (*i.e.*, the maximally distant from 1) values for the regression coefficient *k*, with *k* < 1 (Figure 6B, Supplementary Figures S1–S6). In contrast, other versions of Shambhala-2 (Sh2QBR and Sh2PBR) had *k* > 1 (Figure 6B, Supplementary Figures S1–S6). We have developed and tested the SH2QBR and Sh2RBR modes as the alternatives to the previous mode Sh2PBR (Borisov et al., 2022), since the latter tended to artificially decrease the absolute values of LFC, PAL, and BES metrics.

**FIGURE 8:**
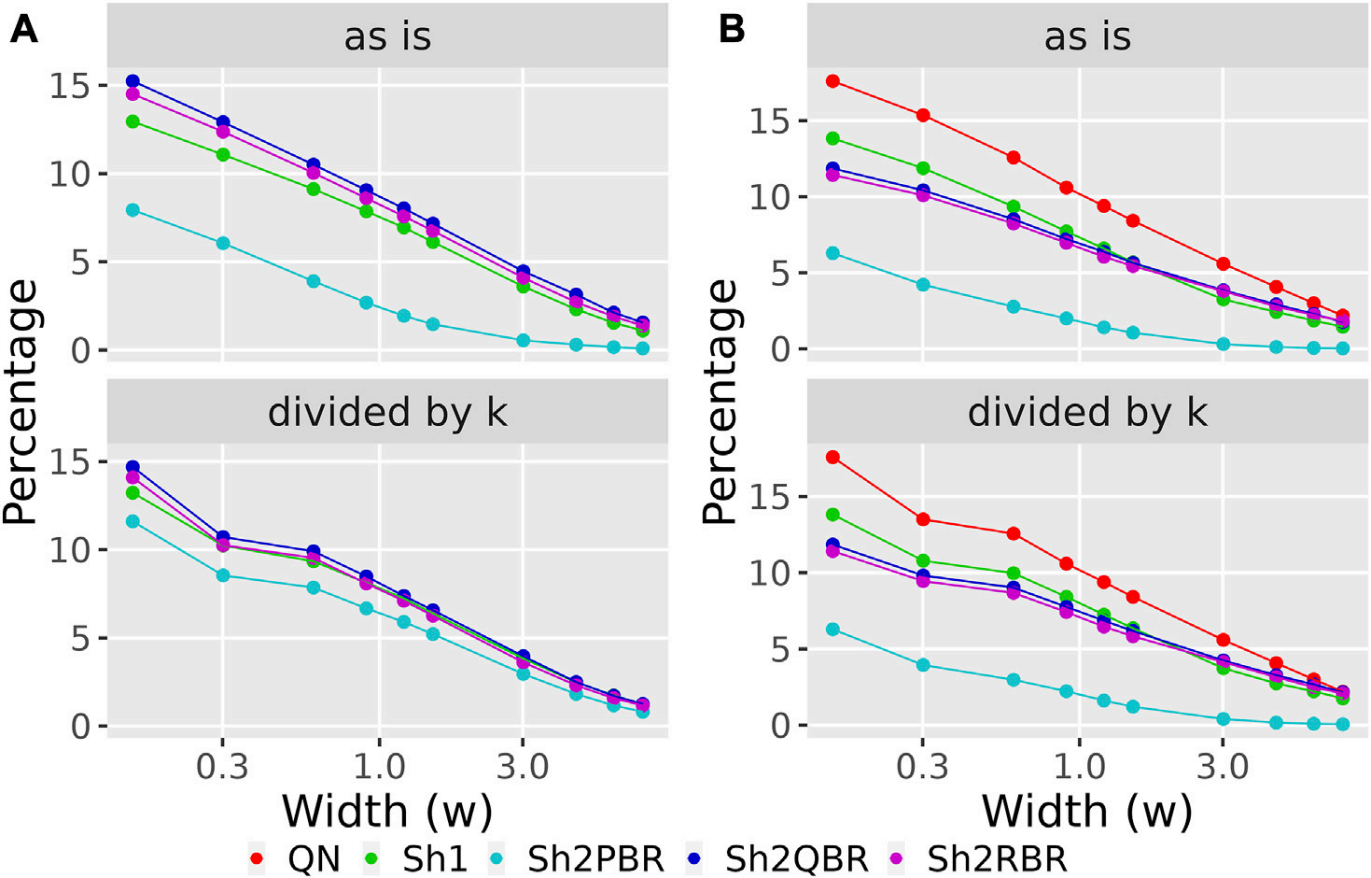
Percentage of BES values, which change their sign when using Shambhala harmonization instead of QN **(A)**, and when using GTEx instead of the ANTE normal references **(B)**, as a function of width (*w*) for the sign-changing significance threshold in all 605 experimental Oncobox samples of 20 cancer types, and 129 anti-cancer target drugs. Mode “as is” is given for BES values without correction. Mode “divided by *k*” is given for BES values divided by the corresponding linear regression coefficients (see Figure 3 for **(A)**; Figure 4 for **(B)**). The width (*w*) was measured in terms of median absolute values of BES for all cancer cases.

The values of regression coefficients, *k*, between BES values with QN and different Shambhala modes, varied significantly: from 0.17 till 1.32 (Figure 6, Supplementary Figures S1–S6). Likewise, the magnitudes of BES varied accordingly. This variation in magnitude lead us to check whether it can affects the sign of BES. The BES value predicts the ability of a drug to inhibit abnormally activated molecular pathways in individual cancer. The positive/negative sign for BES is crucially important, as it indicates the potential beneficial/harmful effect of a certain drug for a certain cancer treatment. We found here that the use of Shambhala harmonization instead of QN has relatively minor effect on the sign of the BES values (Figure 8A, Supplementary Figures S3–S6 in Supplementary Material S6). When the width of sign-changing significance threshold (*w*) around zero is one median value for all BES values in all cases and drugs (from–*w*/2 to + *w*/2), then less than 10% of BES values change their sign. Changing sign of BES is meaningful because it indicates whether a drug is potentially helpful against a particular tumor (BES positive), or not (BES negative or zero).

We considered the BES value as sign-changing for the drug *i* during comparison of two values, *v*_1*i*_ vs. *v*_2*i*_, and a predefined significance threshold width *w*, if *v*_1*i*_ < –*w*/2, and, simultaneously, *v*_2*i*_ > *w*/2, or *vice versa*. With the growth of the significance threshold, the percentage of sign-changing BES values rapidly decreases. The strongest decrease was observed for the QN vs. Sh2PBR comparison (Figure 8A, Supplementary Figures S3–S6 in Supplementary Material S6).

The division of the Shambhala BES values by the regression coefficient *k* increased the percentage of sign-changing events for the Sh2PBR mode. However, even after this division the Sh2PBR mode showed the best results in terms of BES sign stability: compare the options “as is”, *i.e.*, without correction, and “divided by *k*” in Figure 10A, Supplementary Figures S3–S6 in Supplementary Material S6. Interestingly, using Shambhala harmonization instead of QN increased correlation between the BES values calculated for Oncobox cancers vs. ANTE and GTEx norms (Figure 7, Supplementary Figures S2–S6). Moreover, using the GTEx instead of the ANTE normal samples did not change much the BES magnitude, and all regression coefficients (*k*) were close to 1 for the ANTE vs. GTEx normal reference comparison (Figure 7, Supplementary Figures S2–S6).

The proper selection of the *P*-dataset considerably increases the biologically relevant properties of the harmonized data. In our previous study (Borisov et al., 2022), we tried eight different *P*-datasets obtained using different MH and NGS profiling methods, and the Affymetrix Human Genome U133A 2.0 Array-based *P*-dataset apparently showed the best ability for distinguishing the biological nature of samples after harmonization. The relatively good performance of QN in our experiments may be due to the nature of testing gene set including ∼8,000 genes with the greatest expression level, which were included in the *P*-dataset.

In the Oncobox cancer dataset, the same equipment and protocol were used for RNA sequencing as in the ANTE collection of normal samples; instead, different protocol but the same equipment was used for sequencing in GTEx project. Thus, technically ANTE norms better correspond to the Oncobox cancers than GTEx norms for the same tissues. However, the number of Oncobox ANTE normal samples is limited, whereas GTEx tissue samples are much more numerous. Figure 7, Supplementary Figures S2–S6 show that both correlation and linear regression coefficients between the BES values with Oncobox ANTE and GTEx norms are close to 1 for all Shambhala modes, which perform better than QN. Note also the poor correlation values for cervical cancer for the substitution of Oncobox ANTE normal samples with the GTEx samples (Figure 7). This poor correlation may be caused by unusually small number of normal samples (only 4 samples for the Oncobox ANTE collection, and only 18 samples for the GTEx collection), when the sample heterogeneity may play a crucial role.

**FIGURE 9:**
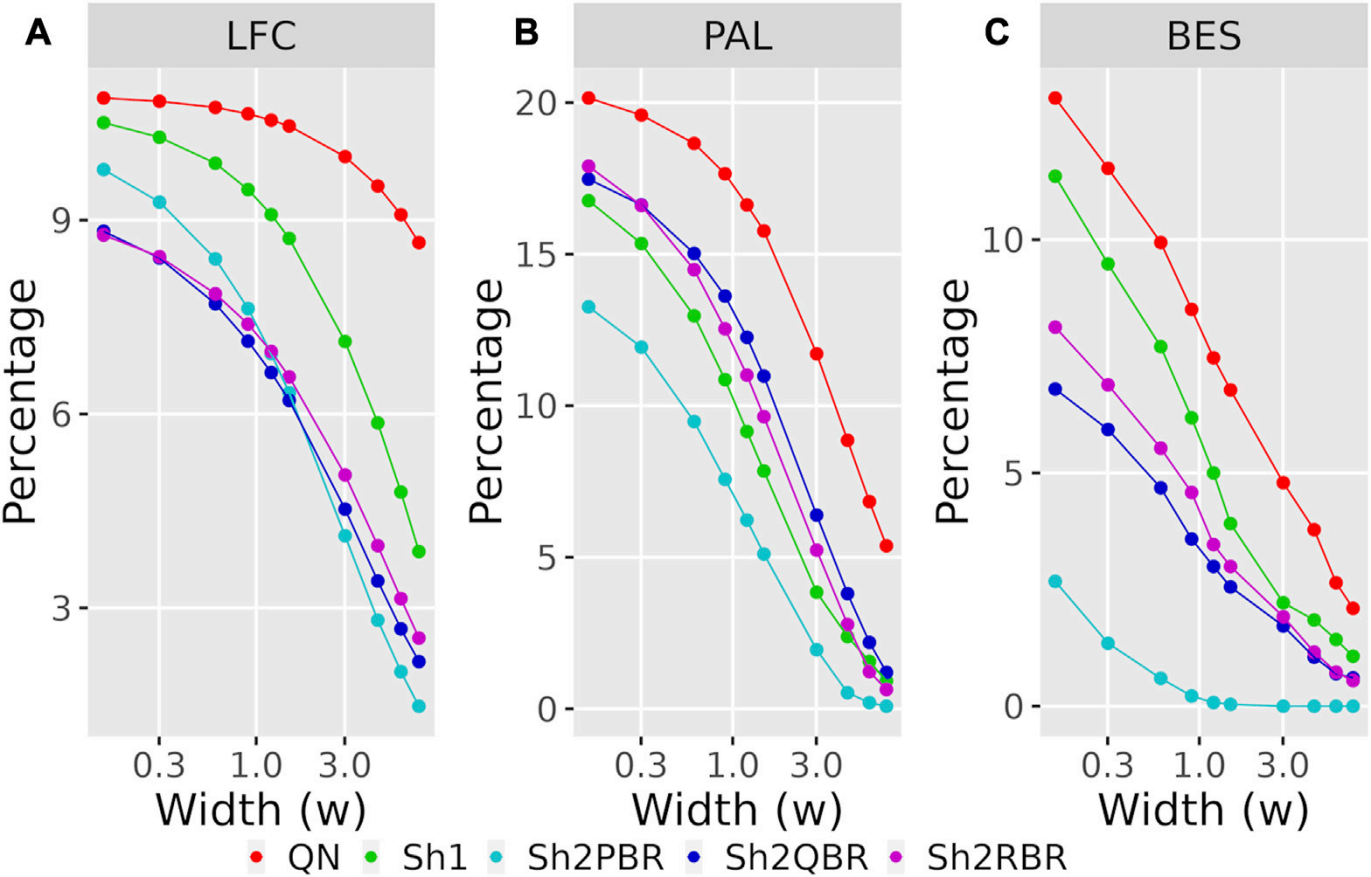
Percentage of LFC **(A)**, PAL **(B)**, and BES **(C)** values, which change their sign when using GTEx instead of the ANTE normal references, as a function of width (*w*) for the sign-changing significance threshold in 74 breast cancer samples with known cancer subtype (hormone-dependent, HERS-positive, and triple negative, Supplementary Material S7). The width (*w*) was measured in terms of median absolute values of LFC, PAL, or BES for breast cancer cases.

Likewise, the use of Shambhala harmonization decreased up to five times the percentage of sign-changing events for BES values calculated using the GTEx instead of the ANTE normal reference samples (Figure 8B, Supplementary Figures S4–S6 in Supplementary Material S6). Again, the Sh2PBR modification showed the best performance (Figure 8B). Note that division of BES values by linear regression coefficient *k* did not affect much the percentage of sign-changing values (Figure 8B, Supplementary Figures S4–S6 in Supplementary Material S6). Note also that the Sh2PBR mode provides the best sign stability over other Shambhala modes and over the standard Oncobox normalization protocol QN at the level of distinct gene LFC and PAL for different pathways (Figure 9, Supplementary Material S7, on the example of breast cancer samples).

We, therefore, conclude that our tests with the case-to-control LFC, PAL, and BES metrics showed that the Sh2PBR mode had the best performance in terms of close to 1 correlation and regression coefficients, and the minimal percentage of sign-changing LFC/PAL/BES values.

### 3.3 Retention of biological properties after uniformly shaped harmonization

**FIGURE 10:**
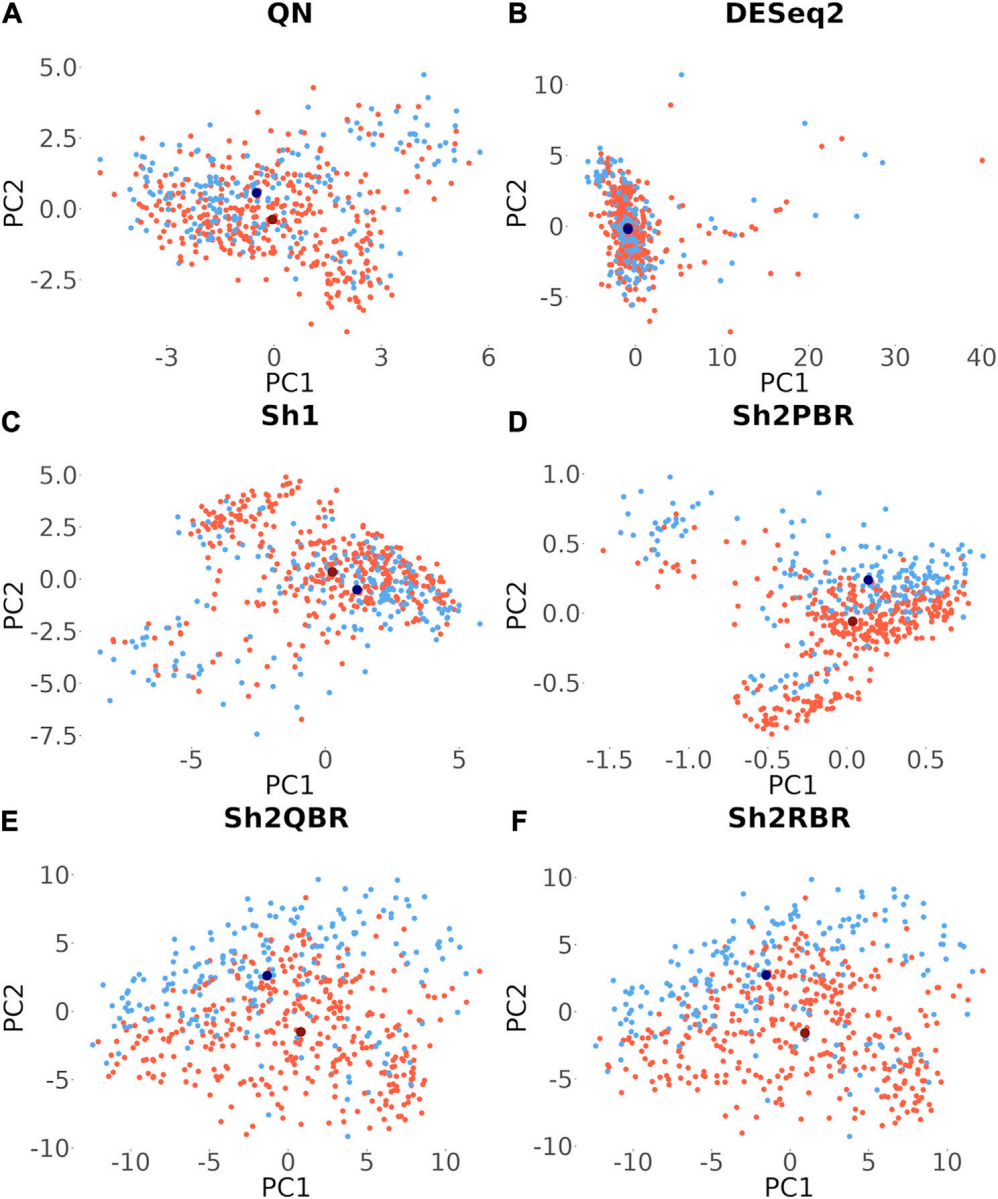
Principle component analysis for the 285 genes located on X chromosome, which survived Shambhala harmonization. Red and blue dots correspond to female and male cancer patients, respectively. Dark dots represent the median values. **(A)** QN, **(B)** DESeq2, **(C)** Sh1, **(D)** Sh2PBR, **(E)** Sh2QBR, **(F)** Sh2RBR.

Besides many statistical performance indicators such as correlation and regression coefficients, sign stability rates, and ML accuracy, it is also important whether the harmonization retains general biological characteristics of biosamples. Otherwise harmonization output will have little sense even when the statistical metrics look acceptable. One such simple test for retention of biological significance can be done with the sex-specific gene expression. Out of 848 X-chromosome genes, 285 survived Shambhala harmonization, and none survived among the 47 Y-chromosome genes (Supplementary Material S8). Using expression of these survivor X-inked genes as the biomarkers, we performed a principal component analysis assay to check for the presence of distinct clusters formed by the male and female patient biosamples from the Oncobox database (for 202 male and 357 female patients). It can be seen from Figure 10 that all Shambhala modes retained sex specific gene expression pattern comparable to QN, and better than for DESeq2.

**FIGURE 11:**
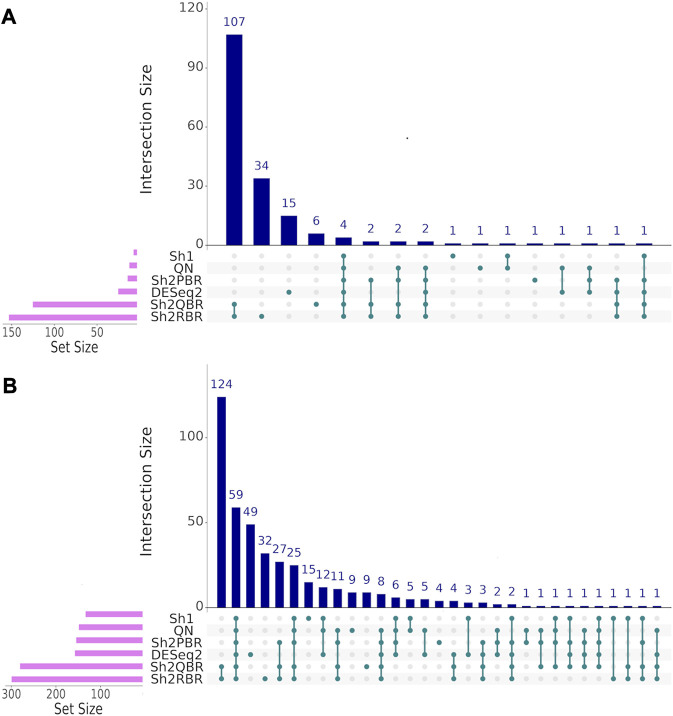
UpSet intersection diagrams (Conway et al., 2017) of marker genes (AUC > 0.80) for the comparison of breast cancer (BC) expression profiles of different molecular subtypes. **(A)** HER2-positive vs. hormone-dependent BC. **(B)** triple negative vs. HER2-positive BC. Along the *x*-axis of the UpSet diagrams, all observed intersections of the analyzed sets are represented. The intersections are marked with turquoise dots, with the rows of the dot matrix corresponding to different sets, and columns showing the intersection combinations. UpSet diagrams were proposed as more informative alternative to traditional Venn diagrams for the analysis of more than four intersecting datasets (Lex et al., 2014).

We also considered the differential gene patterns between different breast cancer molecular subtypes. Although we expect no essential batch effect in the Oncobox data, the overall expression pattern seems tangled. In terms of clustering dendrograms, the hormone-dependent (ESR1 and PGR-positive subtypes), HER2-positive, and triple negative cancer samples are mixed together for all normalization/harmonization methods (Supplementary Material S9). The standard pipelines for differential gene expression analysis (Kuznetsova et al., 2021) seem inappropriate since Shambhala affects the absolute values of LFC. However, Shambhala showed strong ability to retain ROC AUC^2^ metrics between these tumor subtypes. Here we obtained pools of marker genes by calculating AUC (with the threshold of AUC > 0.8) for two pairwise comparisons: (i) HER2-positive vs. hormone-dependent BC; (ii), Triple negative vs. HER2-positive BC. For triple negative vs. HER2-positive, and for HER2-positive vs. hormone-dependent breast cancer, the Sh2QBR and Sh2RBR methods introduced numerous artifacts with the marker genes that were absent for QN. Interestingly, DESeq2 also introduced many genes with AUC > 0.8 for HER2-positive vs. hormone-dependent comparison that were absent for QN, Sh2PBR, and Sh1 (Figure 11, Supplementary Material S10). Table 6 and Supplementary Material S11 show the most frequently prescribed (by the Oncobox drug scoring system) drugs, and most up-and downregulated genes (with gene description) and pathways for three BC subtypes and different harmonization methods.

**TABLE 6:** Most frequently prescribed drugs, according to Oncobox drug scoring system for 74 breast cancer samples and different harmonization methods.

Subtype	Drug	QN	DESeq2	Sh1	Sh2PBR	Sh2QBR	Sh2RBR
Hormone-dependent	1st	Toremifene	Toremifene	Pertuzumab	Toremifene	Perifosine	Erdafitinib
	2nd	Pertuzumab	Pertuzumab	Trastuzumab	Erdafitinib	Toremifene	Perifosine
	3rd	Trastuzumab	Trastuzumab	Erdafitinib	Pertuzumab	Erdafitinib	Toremifene
HER2 positive	1st	Pertuzumab	Pertuzumab	Duvelisib	Pertuzumab	Duvelisib	Duvelisib
	2nd	Trastuzumab	Trastuzumab	Pertuzumab	Trastuzumab	Pertuzumab	Pertuzumab
	3rd	Flavopiridol (Alvocidib)	Flavopiridol (Alvocidib)	Trastuzumab	Flavopiridol (Alvocidib)	Trastuzumab	Trastuzumab
Triple negative	1st	Flavopiridol (Alvocidib)	Flavopiridol (Alvocidib)	Duvelisib	Aflibercept	Duvelisib	Duvelisib
	2nd	Duvelisib	Ethinylestradiol	Flavopiridol (Alvocidib)	Flavopiridol (Alvocidib)	Perifosine	Ethinylestradiol
	3rd	Binimetinib (MEK162)	Bosutinib	Binimetinib (MEK162)	Bevacizumab	Ethinylestradiol	Perifosine

Thus, our tests with the expression of the X-chromosome genes in male and female cancer patients, and with marker genes for the hormone-dependent vs. HER2-positive vs. triple-negative breast cancers confirmed the best performance of the Sh1 and Sh2PBR modes over other methods tested.

## 4 Discussion

Dozens of methods for cross-platform normalization have been developed for the analysis of both microarray hybridization and RNA sequencing gene expression data, yet none of them is currently recognized as an overall gold standard (Borisov and Buzdin, 2022). The majority of these methods return output data in a flexible format (Piccolo et al., 2013; Thompson et al., 2016; Franks et al., 2018; Maleknia et al., 2020; Zhang et al., 2020; Tang et al., 2021; Huang et al., 2022) which requires recalculation of all expression samples every time upon the addition of new gene expression profiles. In search for the universal output format, we developed a novel approach termed Shambhala for uniformly shaped cross-platform harmonization of gene expression data (Borisov et al., 2019; Borisov et al., 2022; Borisov and Buzdin, 2022). The key feature of Shambhala is the one-by-one conversion of each individual profile into the universal shape of the reference definitive dataset independently from the other profiles. This not only creates the basis for unlimited number of further updates of the gene expression bank(s), but also makes it possible to combine together any number of expression datasets. Shambhala allows adding new samples to previous normalized/harmonized data set without the need for renormalizing them, with no currently known limitations for the number of merged datasets and number of samples in such datasets.

In our opinion, the current versions of Shambhala have the following major limitations: limited repertoire of normalized human genes (∼8,000, or ∼40% of all protein-coding genes) and higher calculation costs per individual profile due to the algorithm complexity. However, Shambhala has the following advantage over other normalization methods: its output is returned in a universal format comparable with all other “shambhalized” human profiles obtained using any experimental platform or protocol. This requires no recalculation of the whole dataset upon the addition of new sample(s). In the current version of Shambhala, the maximum amplification of signal-to-noise ratio was prioritized whereas it resulted in a reduced spectrum of genes in the harmonized output. Thus, we added reliability expression filters (Borisov et al., 2019) and the selection of the most highly expressed genes during intersection with the auxiliary datasets (Borisov et al., 2022). The resulting reduced gene set, however, includes 73%–89% of genes participating in molecular pathways, depending on the pathway database (Nishimura, 2001; Schaefer et al., 2009; Croft et al., 2014; Zolotovskaia et al., 2022) and ∼90% of molecular targets of targeted cancer therapeutics (Table 5) which makes such an analysis meaningful for cancer research. However, developing the next versions of Shambhala enabling the high-quality transformation of a greater proportion (ideally all) of human genes, and also non-human genes for other model objects will be a matter of our further studies.

Our previous analysis (Borisov et al., 2022) has demonstrated the strong capacity of the cubic transformation-based Shambhala method (Shambhala-2) to eliminate the platform bias from the clustering dendrograms of gene expression profiles, which assured grouping of the samples according to their biological origin. Here we further assessed the performance of four modifications of Shambhala by applying twelve criteria that characterize the retention of biological functional properties and differential gene expression patterns in thousands of samples from various human tissue types.

Ideally, harmonization of gene expression should provide.

- Clustering of samples according to their biological features, and not according to technical factors like platform/protocol used;
- Close to 1 correlation coefficients for gene expression values in comparison with the initial datasets;
- Close to 1 linear regression coefficients for relative gene expression values and their derivatives like pathway activation levels (PAL) and drug efficiency scores (BES) in comparison with the initial datasets;
- Stability of sign for logarithmic relative gene expression values like fold-change and their derivatives like drug efficiency score in comparison with the initial datasets.
- Conservation or “common sense” biological properties, such as expression levels of sex-related genes, or marker genes between disease subtypes.

In addition, taking into account Big Data analytic approaches, another desirable feature is data suitability for various ML methods, e.g., local and global. An ideal harmonization of gene expression data has to generate similar molecular profiles for the samples of similar biological origin, even when obtained using different equipment and protocols. This means that the ML process trained on the harmonized data obtained using one platform and validated on the harmonized data obtained using another platform should show high overall accuracy. Likewise, the profiles of similar biological origin but obtained using different equipment/protocols, should be strongly correlated.

We compared performance of four available versions of Shambhala with the gold standard flexible output methods QN and DESeq2 according to twelve analytic criteria reflecting the above considerations (Table 7). For most criteria the Sh2PBR mode was either better (criteria #3, 4, 5, 8, 11, and 12) or at least comparable (#1, 2, and 6) than any other method under consideration. Only for the criterion #7, the method Sh2PBR showed unusually low linear regression coefficient *k* ∼ 0.20÷0.25, which indicates that Sh2PBR causes four-to-five-fold decrease of case-to-control log-fold changes in gene expression levels (Figure 6B). However, this proportional variation of case-to-control log-fold changes does not perturb the correlation coefficients, order, and expression ranks of the individual genes and molecular pathways, and also of the predicted drug efficiency scores (Supplementary Figures S1–S6). Moreover, after the Sh2PRB harmonization, the percentage of sign-changed BES values remained lower than all other harmonization methods by approximately 20% (Figures 8A,B).

**TABLE 7:** Twelve performance criteria for versions of Shambhala method with universal gene expression harmonization output in comparison with the flexible output methods QN, DESeq2, and ComBat.

	Criteria	Sh1	Sh2PBR	Sh2QBR	Sh2RBR
1	Prediction accuracy for local ML methods (kNN)	Similar to QN and DESeq2, Better than Combat ∼ 0.87	Similar to QN and DESeq2. Better than Combat ∼ 0.87	Similar to QN and DESeq2. Better than Combat ∼ 0.87	Similar to QN and DESeq2 Better than Combat ∼ 0.87
2	Prediction accuracy for global ML methods (SVM)	Similar to QN and DESeq2. Better than Combat ∼ 0.75	Similar to QN and DESeq2. Better than Combat ∼ 0.75	Similar to QN and DESeq2. Better than Combat ∼ 0.75	Similar to QN and DESeq2. Better than Combat 0.75
3	Quality of hierarchical clustering by biological features	Slightly higher than for QN and DESeq2. Comparable performance to ComBat	Significantly higher than for QN and DESeq2. Comparable performance to ComBat	Similar to QN and DESeq2. Comparable performance to ComBat	Similar to QN and DESeq2. Comparable performance to ComBat
4	Correlation of gene expression profiles before/after harmonization	Comparable to QN, DESeq2, and ComBat ∼0.85	Significantly higher than for QN, DESeq2, and ComBat ∼1	Comparable to QN, DESeq2, and ComBat ∼0.85	Lower than for QN, DESeq2, and ComBat ∼0.80
5	Linear regression coefficient for gene expression profiles before/after harmonization	Similar to QN and DESeq2 ∼0.92	Better than for QN and DESeq2 ∼ 1	Lower than for QN and DESeq2 ∼0.87	Lower than for QN and DESeq2 ∼0.85
6	Correlation of BES values after QN (gold standard) and Shambhala	Similar for all versions of Shambhala ∼0.75	Similar for all versions of Shambhala ∼0.75	Similar for all versions of Shambhala ∼0.75	Similar for all versions of Shambhala ∼0.75
7	Linear regression coefficient for BES values after QN and Shambhala	∼1	∼0.25	∼1.25	∼1.25
8	Retention of positive/negative sign of BES after QN (gold standard) and Shambhala	Drops from 15% to 0% for the width of significance threshold from 0 to 10	The best retention rate, drops from 12% to 0% for the width of significance threshold from 0 to 10	Drops from 15% to 0 for the width of significance threshold from 0 to 10	Drops from 15% to 0 for the width of significance threshold from 0 to 10
9	Correlation of BES values using ANTE and GTEx normal reference sets	Higher than for QN by approximately 20%	Higher than for QN by approximately 20%	Higher than for QN by approximately 20%	Higher than for QN by approximately 20%
10	Linear regression coefficient of BES values for ANTE and GTEx normal reference	∼1.15	∼0.85	∼0.85	∼0.85
11	Retention of positive/negative sign of BES after harmonization and using ANTE or GTEx normal reference	About 40% higher than QN	More than 2 times higher than QN, about two times higher than any other version of Shambhala	About 40% higher than QN	About 40% higher than QN
12	Retention of biological properties	Similar to QN, better than DESeq2	Similar to QN, better than DESeq2	Worse than QN and DESeq2: introduced many artifact marker genes	Worse than QN and DESeq2: introduced many artifact marker genes

In Table 8 we summarized the recommendations for the use of different normalization/harmonization methods. Overall, our correlation, regression, and sign-change analysis has demonstrated the best results of the Sh2PBR version of Shambhala. We, therefore, suggest that Sh2PBR can be considered as the method of choice for harmonization of various types of human gene expression data.

**TABLE 8:** Recommendations for applications of gene expression normalization/harmonization methods.

Reference	Method	Mathematical principle	Algorithmic complexity	Advantages	Shortcomings
Bolstad et al. (2003)	Quantile normalization (QN)	Ranking the expression levels of different genes within each profile and setting the expression level of each gene to the mean value (over all profiles) for the respective rank	Relatively simple	Gold standard method for intra-platform normalization of the MH data	Avoiding being used for cross-platform harmonization of the MH data; requiring recalculation of all gene expression-based values after addition of new samples
Love et al. (2014)	Differential Gene Expression in Sequencing 2 (DESeq2)	Transform based on the negative binomial distribution	Moderately complex	Gold standard for intra-platform normalization of RNAseq data	Requiring recalculation of all gene expression-based values after addition of new samples
Borisov et al. (2019)	Shambhala-1 (linear Shambhala)	Uniformly shaped harmonization based on the XPN (Shabalin et al., 2008) method	Complex	Working for harmonization of unlimited number of datasets of any size, for both MH and RNAseq data or their combinations; not requiring recalculation of gene expression-based values after addition of new samples	Resource-demanding. Reduces the number of protein-coding genes in the harmonized output down to ∼8000 items
Borisov et al. (2022)	Shambhala-2 (cubic Shambhala)	Uniformly shaped harmonization based on the CuBlock (Junet et al., 2021) method	Complex	Working for harmonization of the unlimited number of datasets of any size, for both MH and RNAseq data or their combinations; not requiring recalculation of gene expression-based values after addition of new samples	Resource-demanding. Reduces the number of protein-coding genes in the harmonized output down to ∼8000 items

## Data Availability

Publicly available datasets were analyzed in this study. This data can be found here: https://zenodo.org/record/6415067.

## Footnotes

1 QN is a standard normalization technique in the Oncobox method for calculation of drug efficiency scores.

2 The ROC (receiver–operator curve) is a widely-used graphical plot that illustrates the diagnostic ability of a binary classifier system as its discrimination threshold is varied. The ROC is created by plotting the true positive rate (TPR) against the false positive rate (FPR) at various threshold settings. The area under the ROC curve, called ROC AUC, or simply AUC, is routinely employed for assessment of any classifier’s quality.

## Abbreviations

ANTE, Atlas of Normal Tissue Expression; AUC, Area under Curve; AUROC, Area under Receiver-Operator Curve; BC, Breast Cancer; BES, Balanced Efficiency Score; CuBlock, Cubic Blocks; DES, Drug Efficiency Score; DESeq2, Differential gene Expression Analysis in Sequencing 2; ER, estrogen receptor; GTEx, Genotype-Tissue Expression; kNN, *k* Nearest Neighbors; LFC, log-fold change; MH, Microarray Hybridization; NGS, Next-Generation Sequencing; PAL, Pathway Activation Level; PR, progesterone receptor; QN, Quantile NormalizationRNAseq; RNA, sequencing; ROC, Receiver-Operator Curve; Sh1, Shambhala-1; Sh2, Shambhala-2; Sh2PBR, Shambhala-2 with P-Based Rescaling; Sh2QBR, Shambhala-2 with Q-Based Rescaling; Sh2RBR, Shambhala-2 with R-Based Rescaling; SVM, Support Vector Machine; TCGA, The Cancer Genome Atlas; TDM, Training Distribution Machine; UPC, Universal Expression Code; WM, Watermelon Multisection; XPN, Cross-Platform Normalization.
