# Supplementary material for: Uniformly shaped harmonization combines human transcriptomic data from different platforms while retaining their biological properties and differential gene expression patterns
Source: Front Mol Biosci. 2023 Sep 6;10:1237129. doi: 10.3389/fmolb.2023.1237129 (PMC10511763; doi:10.3389/fmolb.2023.1237129)
Supplement: Supplementary file 1 [file DataSheet6.docx]

Supplementary Material 6

Uniformly shaped harmonization combines human transcriptomic data from different platforms while retaining their biological properties and differential gene expression patterns

Nicolas Borisov, Victor Tkachev, Alexander Simonov, Maxim Sorokin, Ella Kim, Denis Kuzmin, Betul Karademir-Yilmaz, and Anton Buzdin

*** Correspondence:** Nicolas Borisov. [nicolasborissoff@gmail.com](mailto:nicolasborissoff@gmail.com)


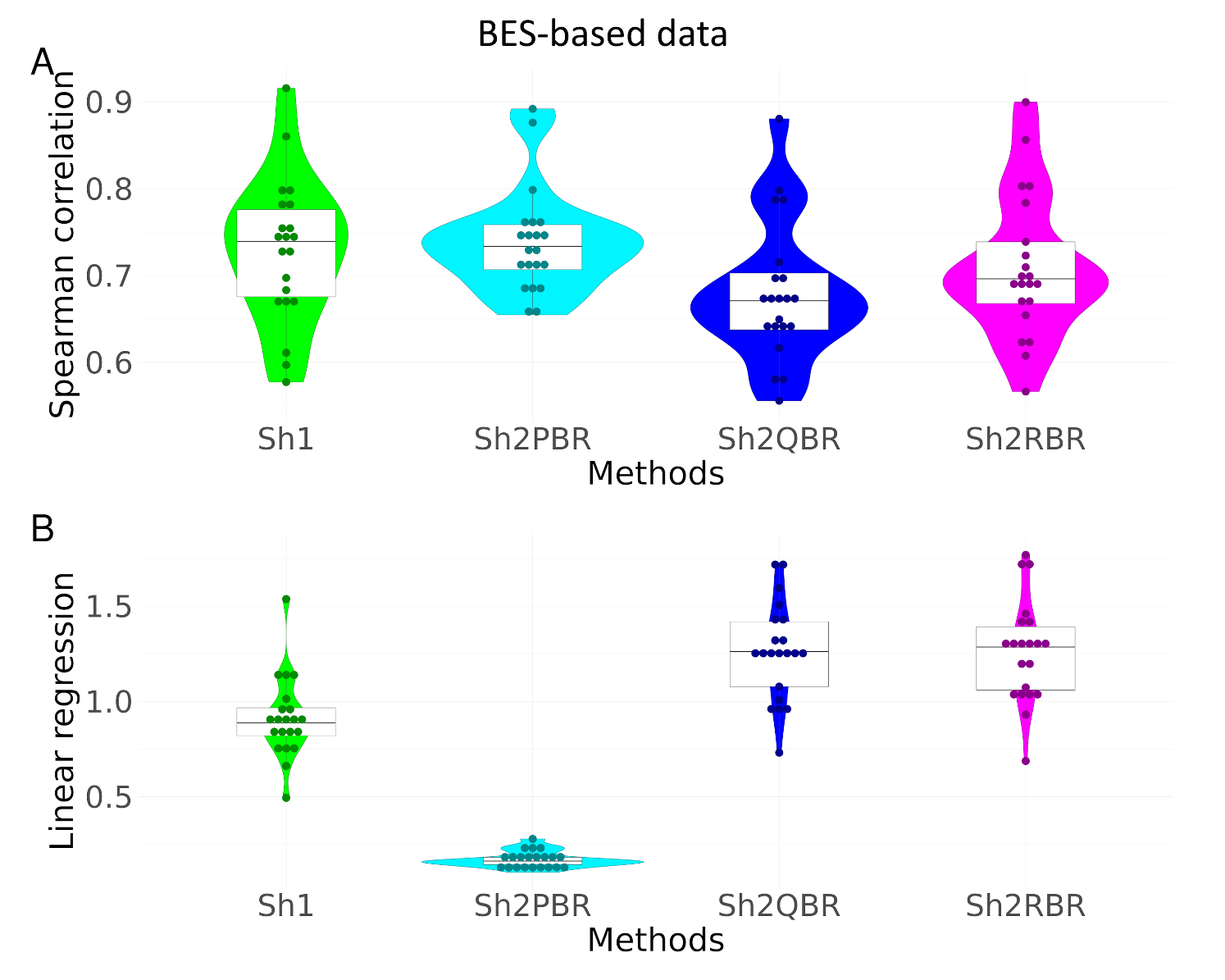


***Supplementary Fig. 6-1***. Comparison of BES values for QN vs Shambhala comparison of 20 cancer types and 129 targeted cancer drugs A: distribution of Spearman correlation coefficients. B: distribution of linear regression coefficient.


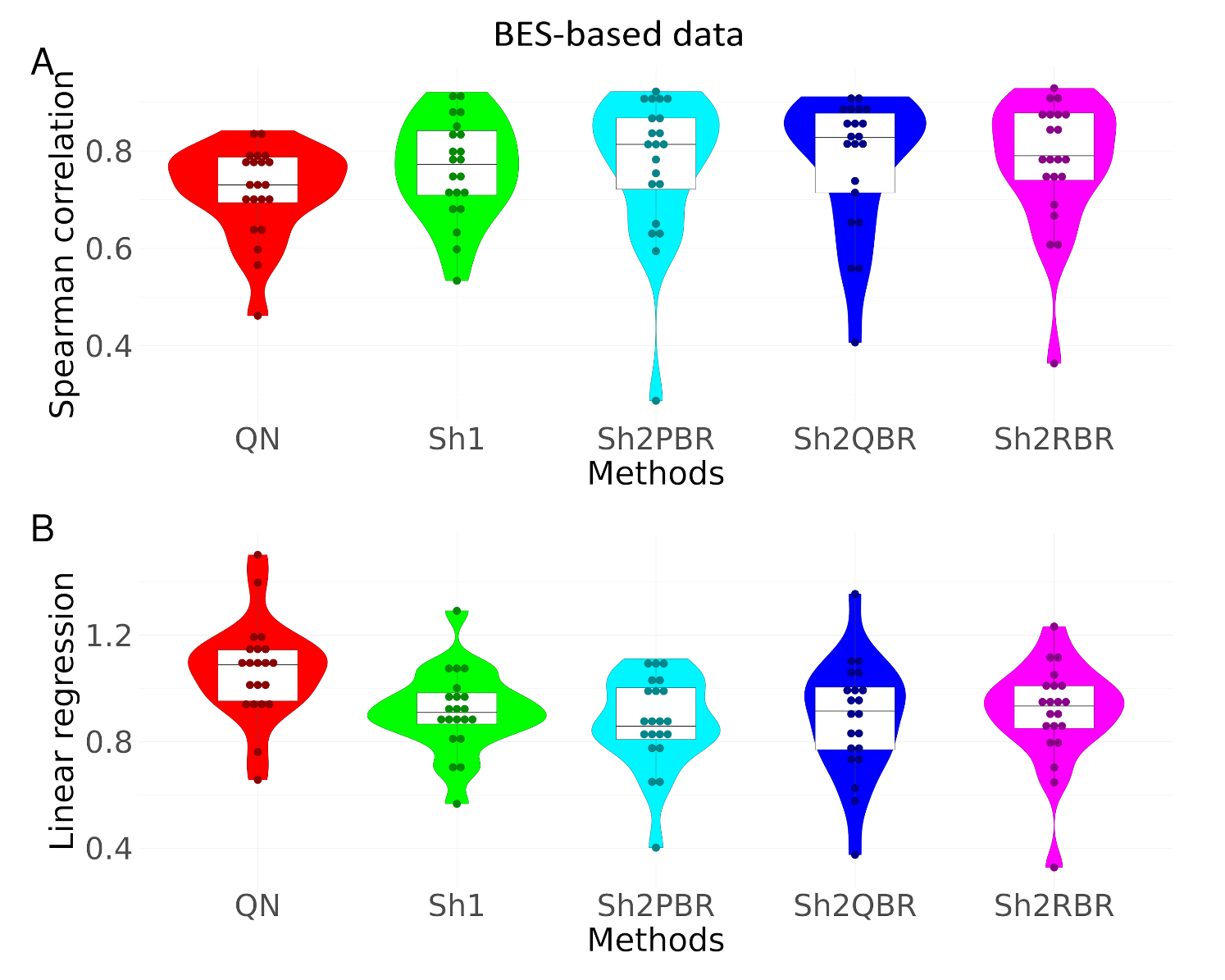


***Supplementary Fig. 6-2.*** Comparison of BES values for BES values calculated with ANTE vs. GTEx normal references for 20 cancer types and 129 targeted cancer drugs A: distribution of Spearman correlation coefficients. B: distribution of linear regression coefficients.

***
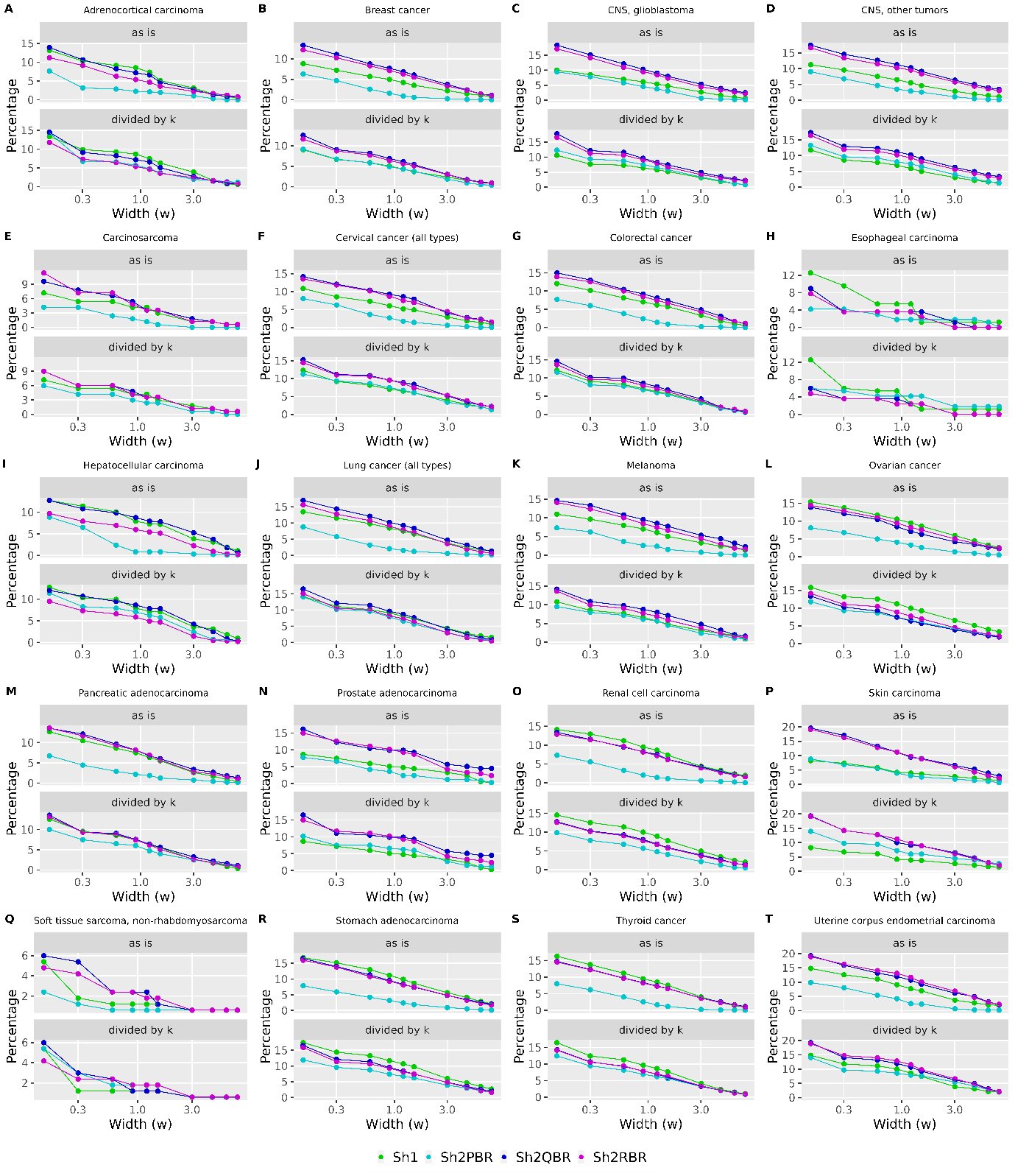
***

***Supplementary Fig. 6-3.*** Percentage of BES values, which change the sign when using Shambhala harmonization instead of QN, as a function of the width (*w*) for the sign-changing significance threshold for 20 different cancer types and 129 anti-cancer target drugs. Mode “as is”: BES values without correction. Mode “divided by *k*”: BES values divided by the corresponding linear regression coefficients (see Fig. 3 in the Main text). The width (*w*) was measured in terms of median absolute values of BES for all cancer cases.

***
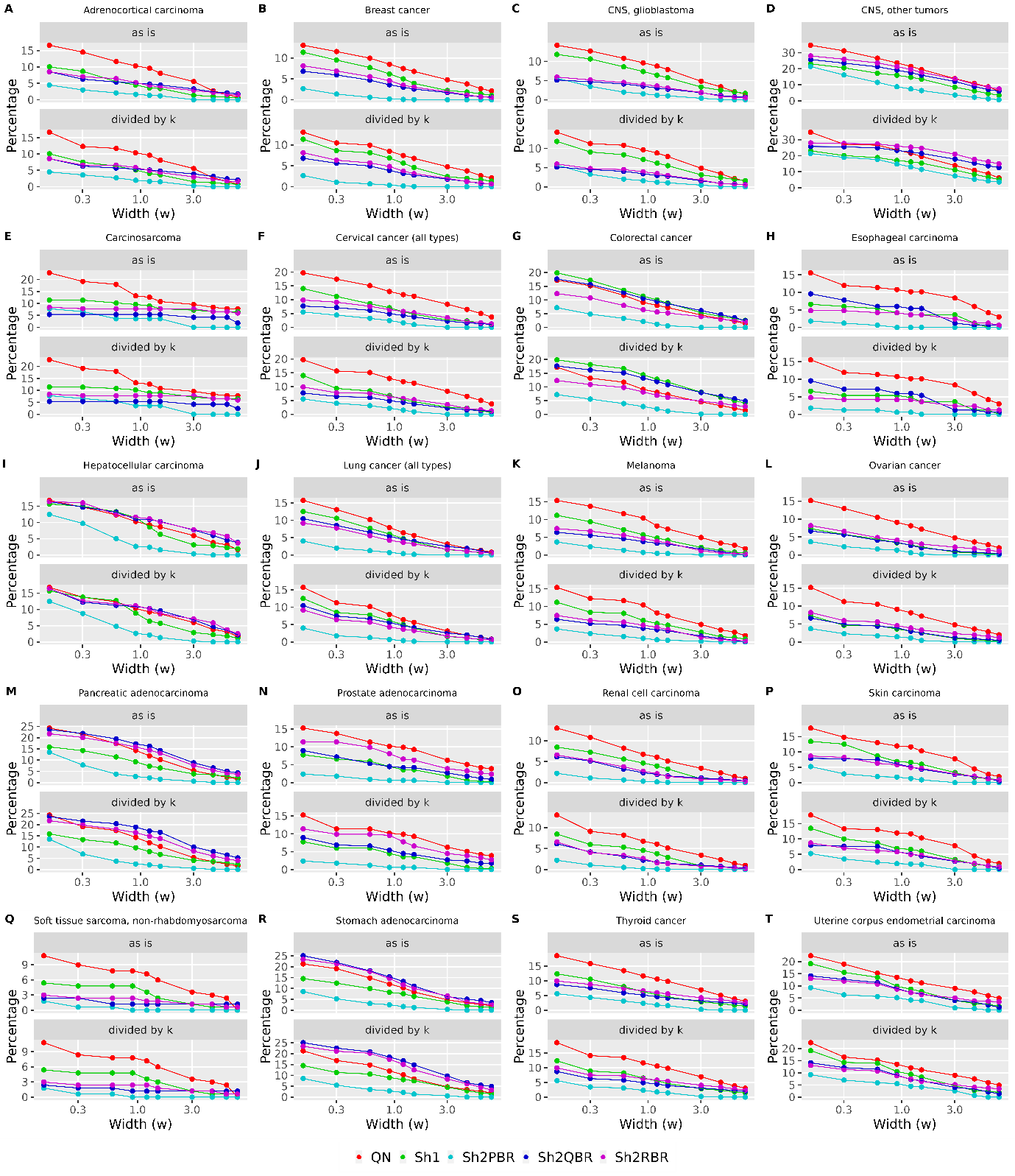
***

***Supplementary Fig. 6-4.*** Percentage of BES values, which change the sign when using GTEx instead of the ANTE normal references, as a function of the width (*w*) for the sign-changing significance threshold for 20 different cancer types and 129 anti-cancer target drugs. Mode “as is”: BES values without correction. Mode “divided by *k*”: BES values divided by the corresponding linear regression coefficients (see Fig. 4 in the Main text). The width (*w*) was measured in terms of median absolute values of BES for all cancer cases.
