# Supplementary material for: Uniformly shaped harmonization combines human transcriptomic data from different platforms while retaining their biological properties and differential gene expression patterns
Source: Front Mol Biosci. 2023 Sep 6;10:1237129. doi: 10.3389/fmolb.2023.1237129 (PMC10511763; doi:10.3389/fmolb.2023.1237129)
Supplement: Supplementary file 4 [file DataSheet3.docx]

Supplementary Material 4

Uniformly shaped harmonization combines human transcriptomic data from different platforms while retaining their biological properties and differential gene expression patterns

Nicolas Borisov, Victor Tkachev, Alexander Simonov, Maxim Sorokin, Ella Kim, Denis Kuzmin, Betul Karademir-Yilmaz, and Anton Buzdin

*** Correspondence:** Nicolas Borisov. [nicolasborissoff@gmail.com](mailto:nicolasborissoff@gmail.com)

***
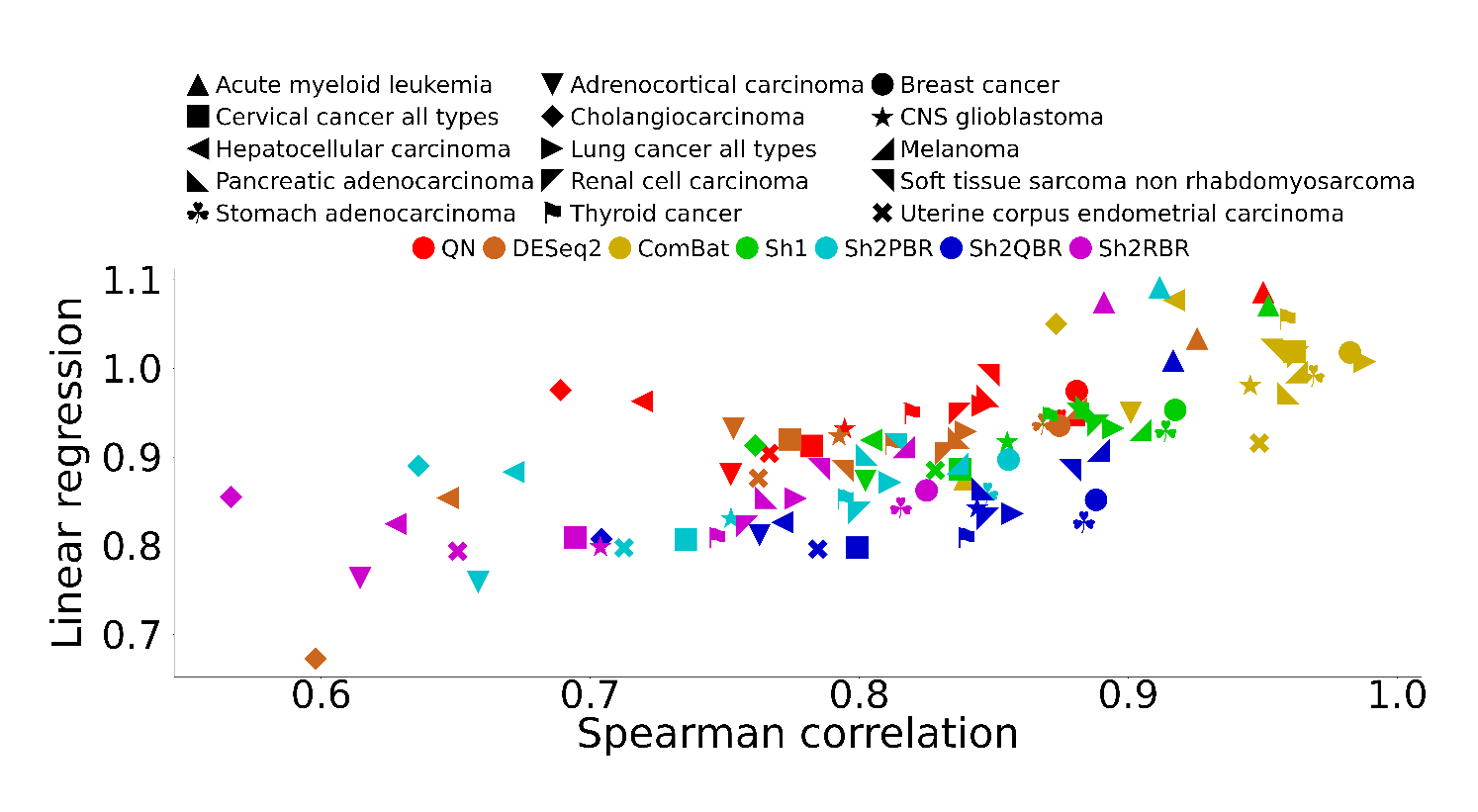
Supplementary Fig. 3-1.*** Distribution of Spearman correlation vs linear regression coefficients between the median log-expression profiles for the TCGA (Tomczak et al., 2015) and Oncobox (Borisov et al., 2022) cancer datasets, and different normalization/harmonization modes.

***
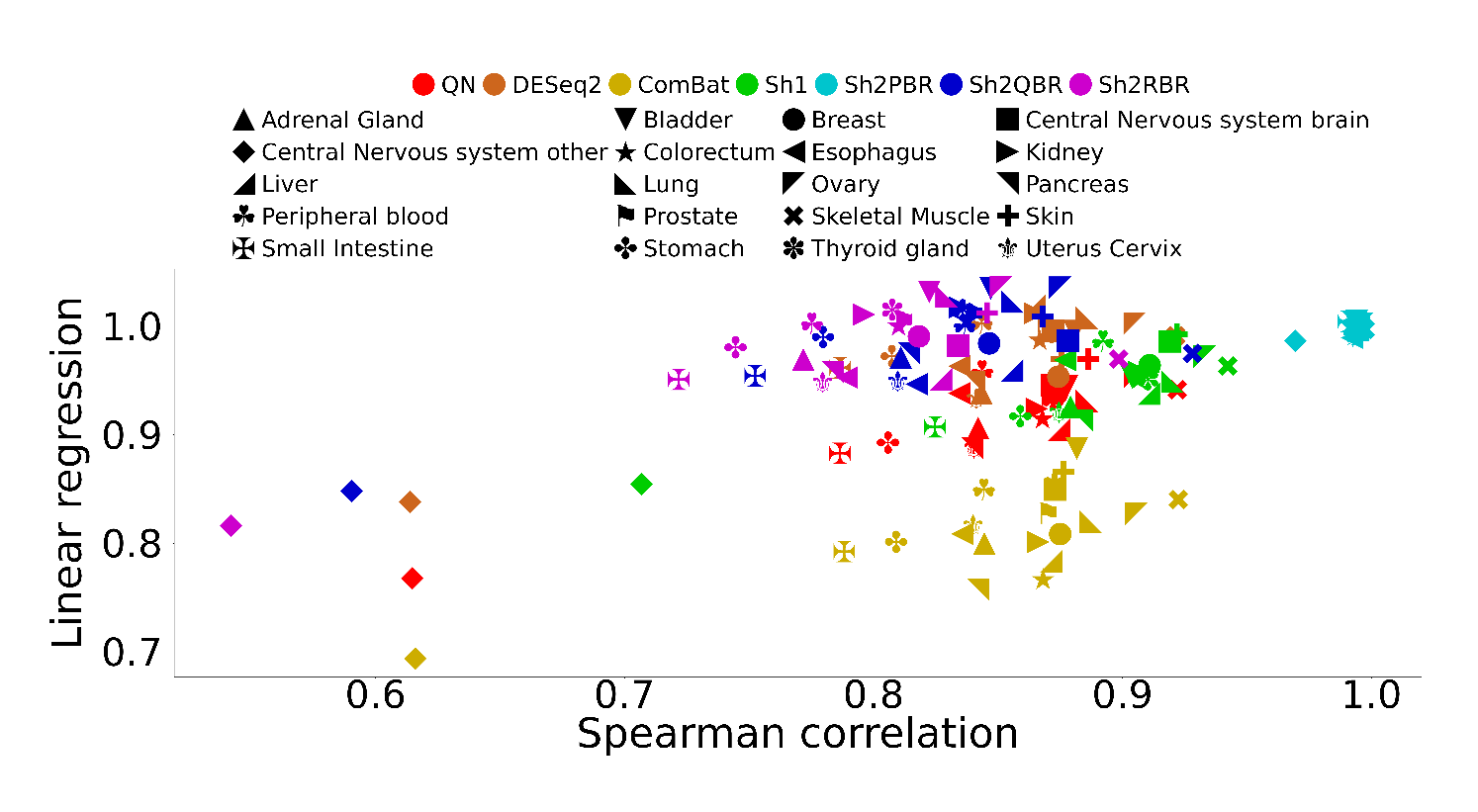
Supplementary Fig. 3-2.*** Distribution of Spearman correlation vs linear regression coefficients between the median log-expression profiles for the GTEx NGS (GTEx Consortium, 2013) and TCGA (Tomczak et al., 2015) normal tissue datasets, and different normalization/harmonization modes.

***
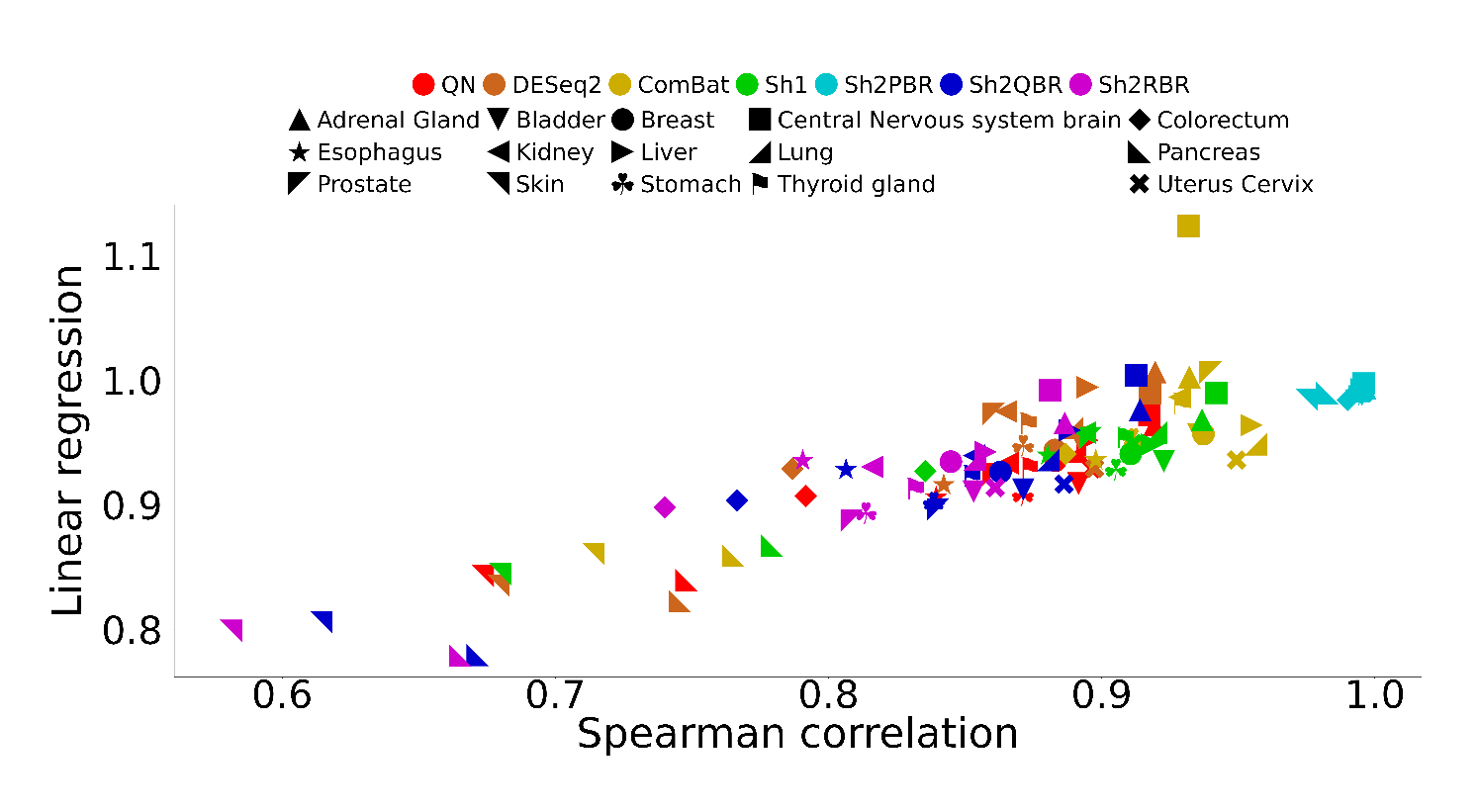
Supplementary Fig. 3-3.*** Distribution of Spearman correlation vs linear regression coefficients between the median log-expression profiles for the GTEx NGS (GTEx Consortium, 2013) and Atlas of Normal Tissue Expression (ANTE) (Suntsova et al., 2019) normal tissue datasets, and different normalization/harmonization modes.


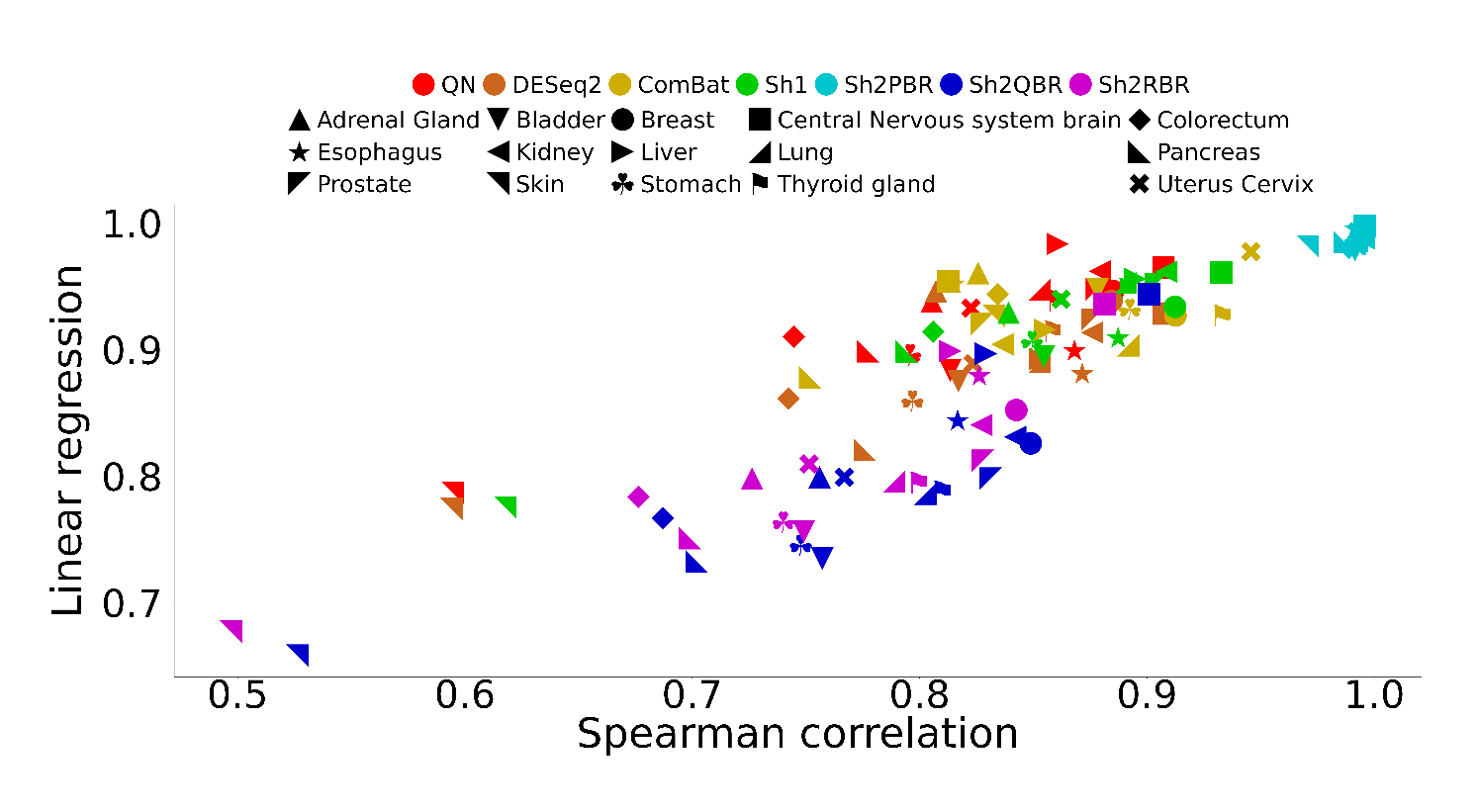


***Supplementary Fig. 3-4.*** Distribution of Spearman correlation vs linear regression coefficients between the median log-expression profiles for the TCGA (Tomczak et al., 2015) and Atlas of Normal Tissue Expression (ANTE) (Suntsova et al., 2019) normal tissue datasets, and different normalization/harmonization modes.


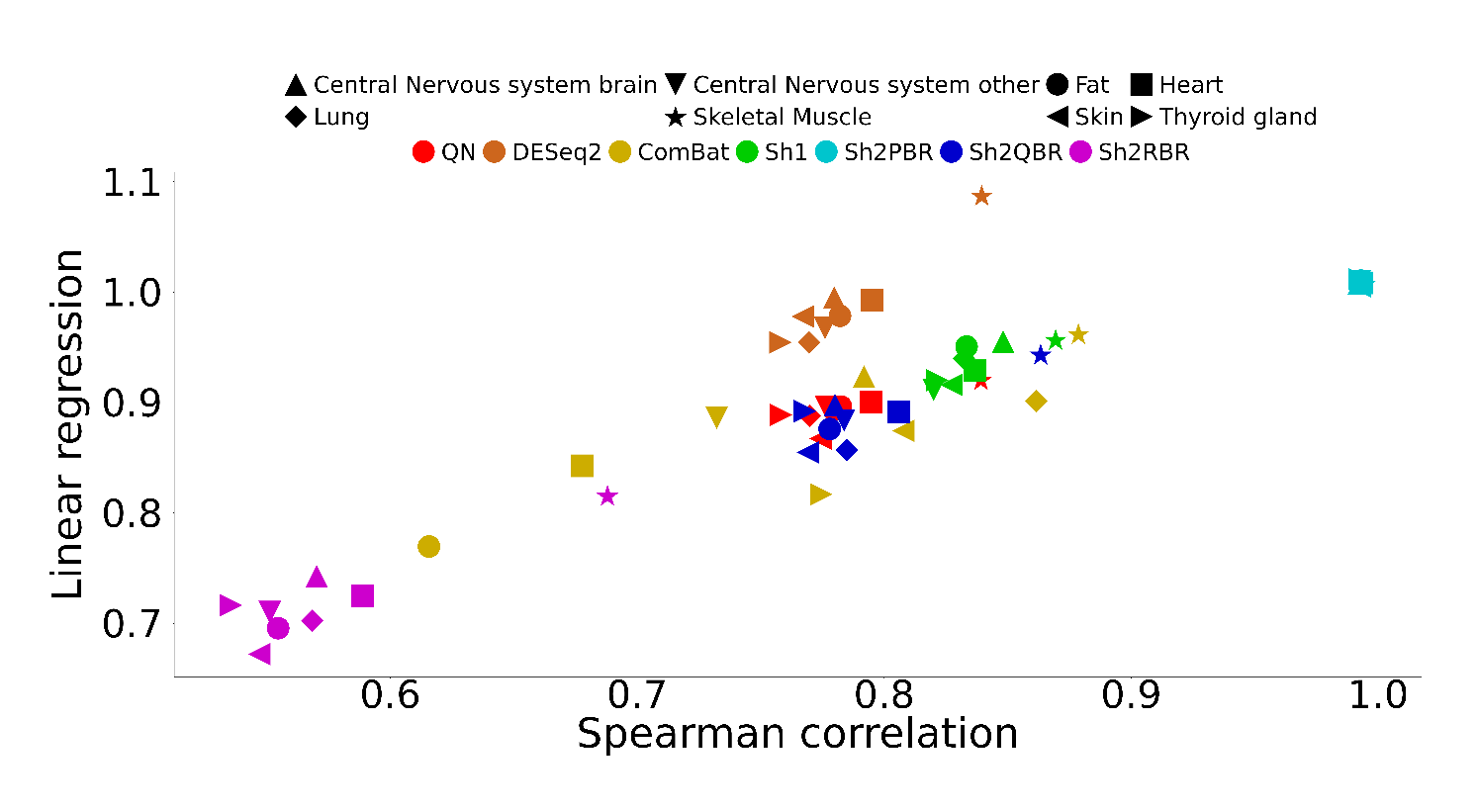


***Supplementary Fig. 3-5.*** Distribution of Spearman correlation vs linear regression coefficients between the median log-expression profiles for the GTEx NGS (GTEx Consortium, 2013) and GTEx Affymetrix HUG1 (GTEx Consortium, 2013). (Suntsova et al., 2019) normal tissue datasets, and different normalization/harmonization modes.


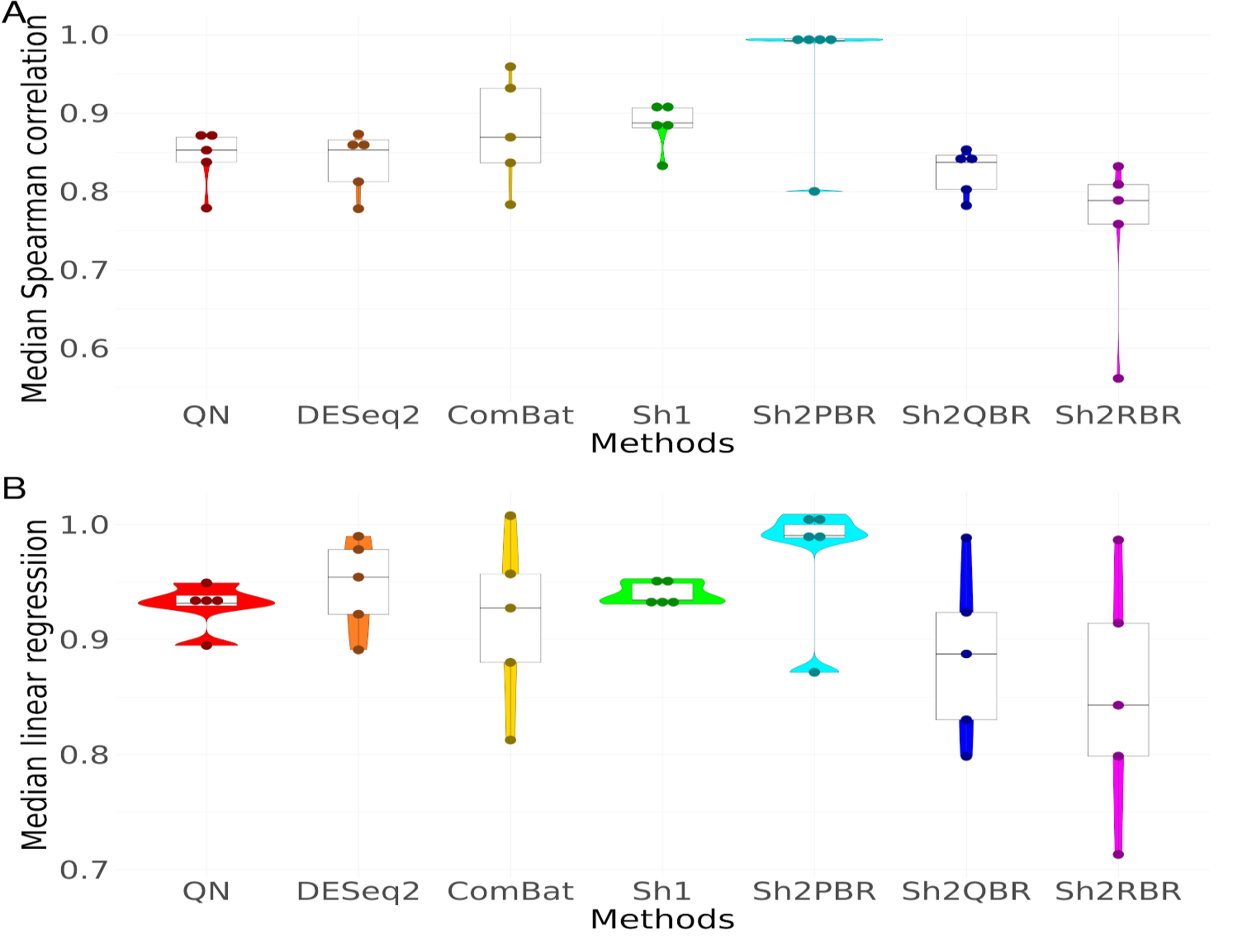


***Supplementary Fig. 3-6***. Distribution of median (over all possible tissue types) Spearman correlation and linear regression coefficients between the same tissue types from training and validation datasets A: Spearman correlation coefficients; B: linear regression coefficients.

**References**

Borisov, N., Sorokin, M., Zolotovskaya, M., Borisov, C., and Buzdin, A. (2022). Shambhala‐2: A Protocol for Uniformly Shaped Harmonization of Gene Expression Profiles of Various Formats. *Current Protocols* 2. doi: 10.1002/cpz1.444.

GTEx Consortium (2013). The Genotype-Tissue Expression (GTEx) project. *Nature Genetics* 45, 580–585. doi: 10.1038/ng.2653.

Suntsova, M., Gaifullin, N., Allina, D., Reshetun, A., Li, X., Mendeleeva, L., et al. (2019). Atlas of RNA sequencing profiles for normal human tissues. *Scientific Data* 6. doi: 10.1038/s41597-019-0043-4.

Tomczak, K., Czerwinska, P., and Wiznerowicz, M. (2015). The Cancer Genome Atlas (TCGA): an immeasurable source of knowledge. *Contemporary Oncology (Poznan, Poland)* 19, A68–A77. doi: 10.5114/wo.2014.47136.
