# Supplementary material for: Uniformly shaped harmonization combines human transcriptomic data from different platforms while retaining their biological properties and differential gene expression patterns
Source: Front Mol Biosci. 2023 Sep 6;10:1237129. doi: 10.3389/fmolb.2023.1237129 (PMC10511763; doi:10.3389/fmolb.2023.1237129)
Supplement: Supplementary file 5 [file DataSheet9.docx]

Supplementary Material 8

Uniformly shaped harmonization combines human transcriptomic data from different platforms while retaining their biological properties and differential gene expression patterns

Nicolas Borisov, Victor Tkachev, Alexander Simonov, Maxim Sorokin, Ella Kim, Denis Kuzmin, Betul Karademir-Yilmaz, and Anton Buzdin

*** Correspondence:** Nicolas Borisov. [nicolasborissoff@gmail.com](mailto:nicolasborissoff@gmail.com)


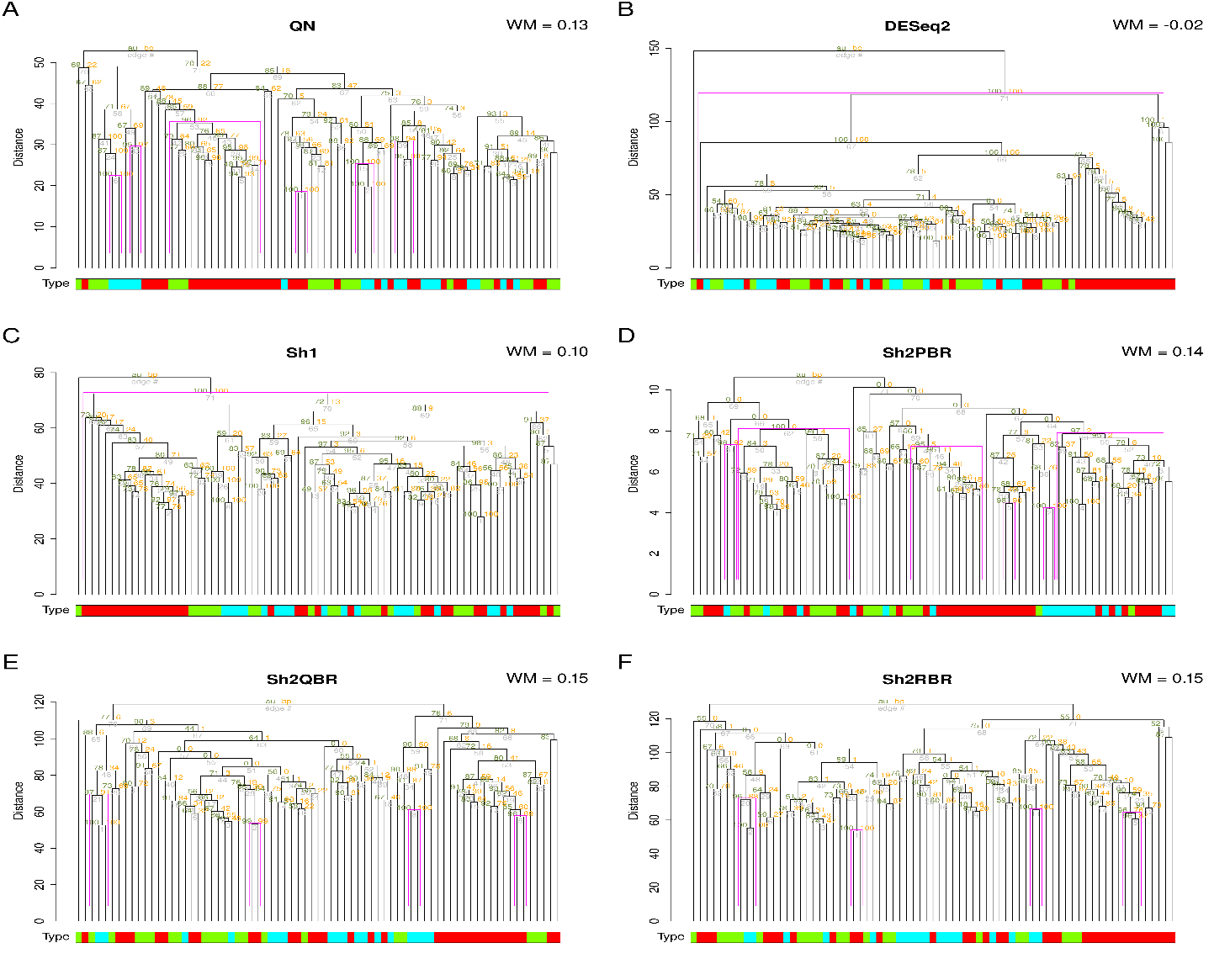


***Supplementary Fig. 8-1.*** Clustering dendrograms for the Oncobox breast cancer (BC) samples after different methods of harmonization. Green – hormone-dependent BC (ER, PR, or both). Red - HER2-dependent BC. Cyan – triple negative BC. WM – watermelon multi-section metric (Zolotovskaia et al., 2020) for the given cancer types.

**References**

Zolotovskaia, M. A., Sorokin, M. I., Petrov, I. V., Poddubskaya, E. V., Moiseev, A. A., Sekacheva, M. I., et al. (2020). Disparity between Inter-Patient Molecular Heterogeneity and Repertoires of Target Drugs Used for Different Types of Cancer in Clinical Oncology. *International Journal of Molecular Sciences* 21, 1580. doi: 10.3390/ijms21051580.
