# Supplementary material for: Uniformly shaped harmonization combines human transcriptomic data from different platforms while retaining their biological properties and differential gene expression patterns
Source: Front Mol Biosci. 2023 Sep 6;10:1237129. doi: 10.3389/fmolb.2023.1237129 (PMC10511763; doi:10.3389/fmolb.2023.1237129)
Supplement: Supplementary file 8 [file DataSheet2.docx]

Supplementary Material 2

Uniformly shaped harmonization combines human transcriptomic data from different platforms while retaining their biological properties and differential gene expression patterns

Nicolas Borisov, Victor Tkachev, Alexander Simonov, Maxim Sorokin, Ella Kim, Denis Kuzmin, Betul Karademir-Yilmaz, and Anton Buzdin

*** Correspondence:** Nicolas Borisov. [nicolasborissoff@gmail.com](mailto:nicolasborissoff@gmail.com)


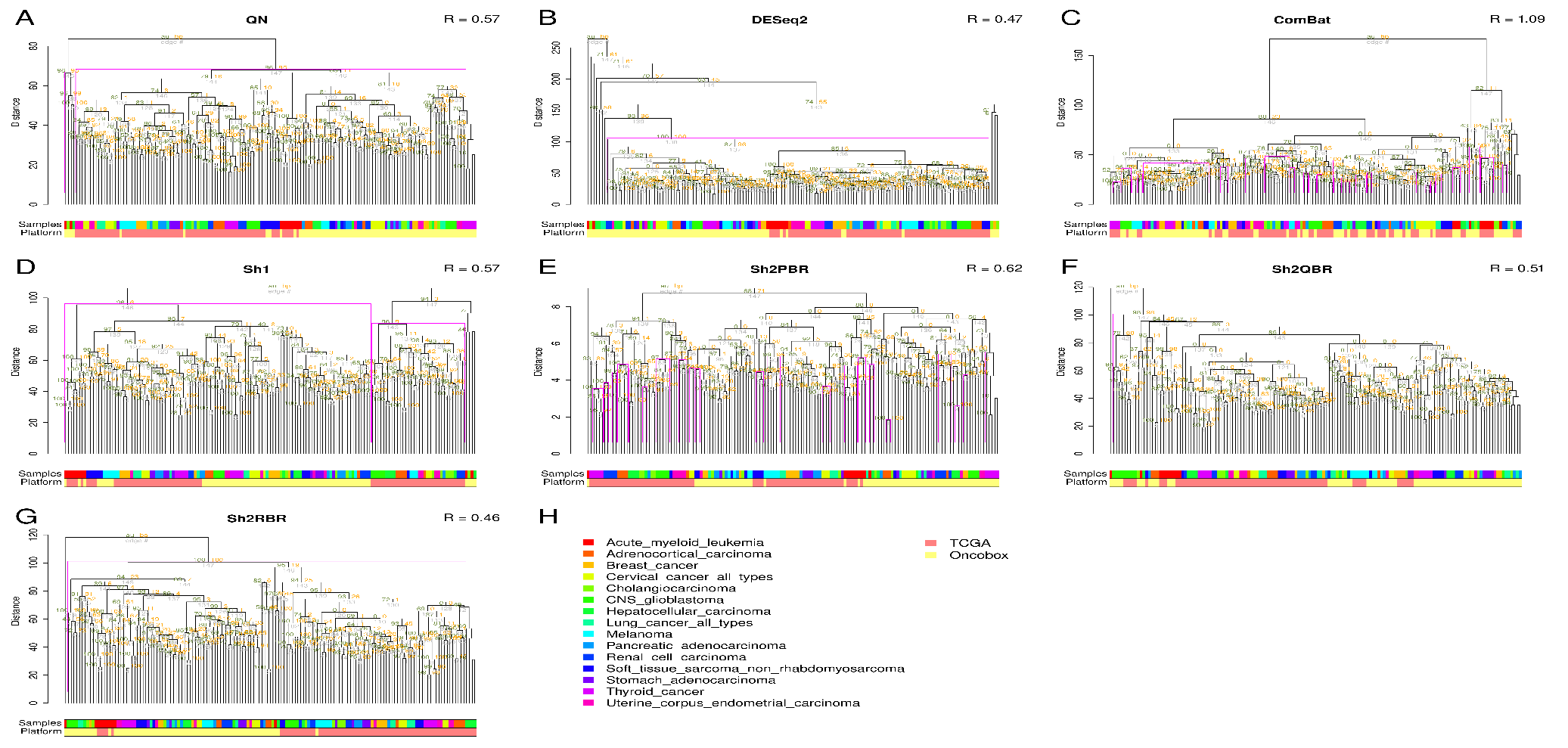


***Supplementary Fig. 2-1.*** Clustering dendrograms for merged cancer datasets TCGA (Tomczak et al., 2015), and Oncobox (Borisov et al., 2022). Normalization/harmonization methods: QN (A), DESeq2 (B), ComBat (C), Sh1 (D), Sh2PBR (E), Sh2QBR (F), Sh2RBR (G). H: legend for the sample types and batches. The ratio of sample-based to platform-based WM metrics is shown in the upper right corner of each panel.


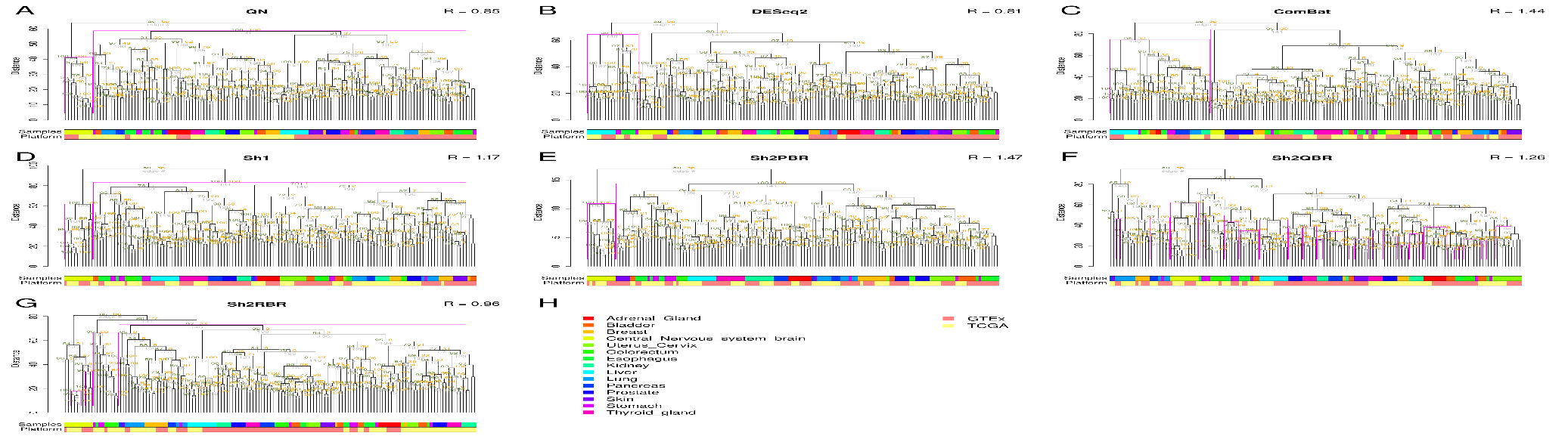


***Supplementary Fig. 2-2.*** Clustering dendrograms for merged normal/control datasets GTEx NGS (GTEx Consortium, 2013), and TCGA (Tomczak et al., 2015). Normalization/harmonization methods: QN (A), DESeq2 (B), ComBat (C), Sh1 (D), Sh2PBR (E), Sh2QBR (F), Sh2RBR (G). H: legend for the sample types and batches. The ratio of sample-based to platform-based WM metrics is shown in the upper right corner of each panel.


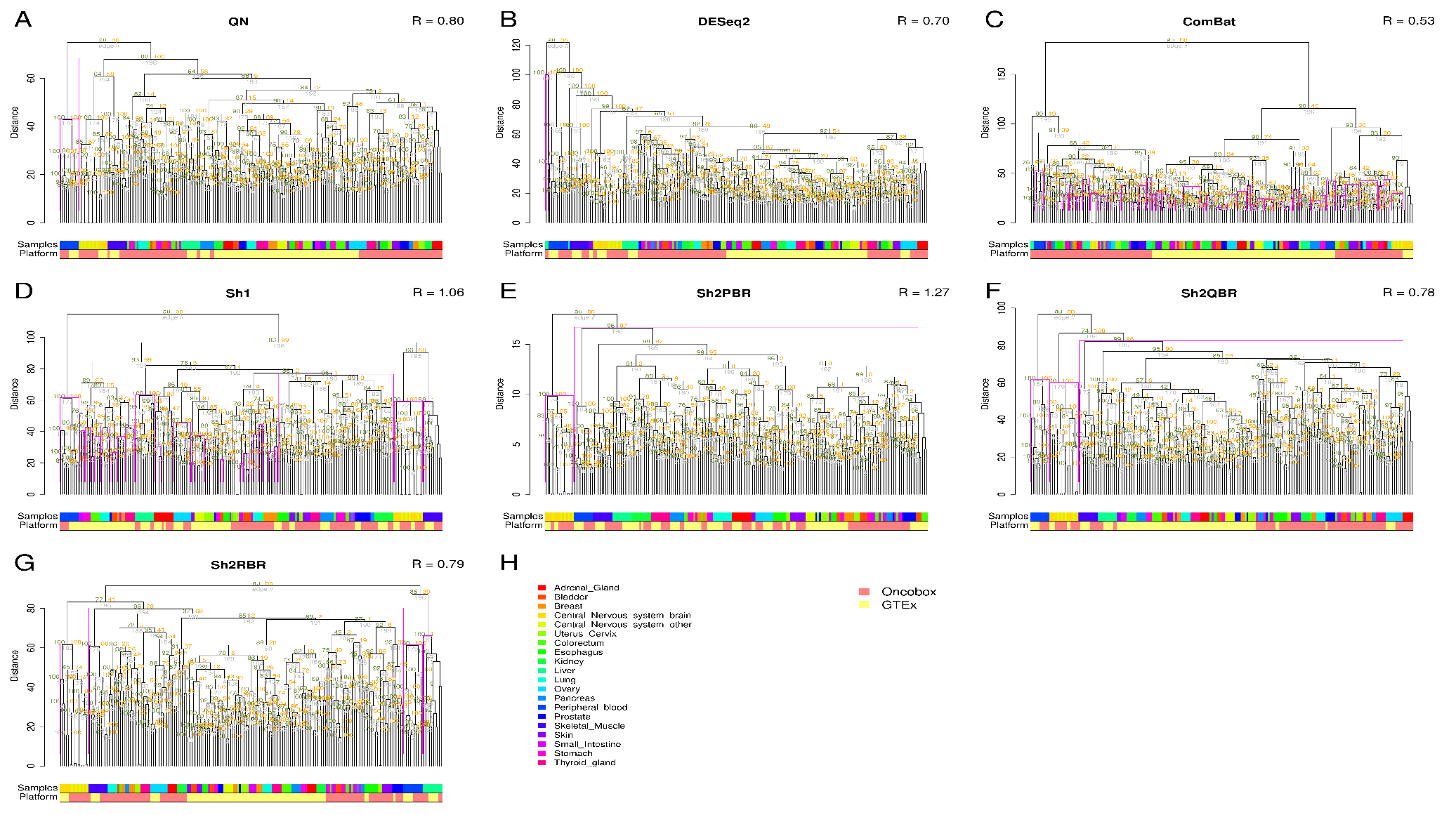


***Supplementary Fig. 2-3.*** Clustering dendrograms for merged normal/control datasets GTEx NGS (GTEx Consortium, 2013), and Oncobox Atlas of Normal Tissue Expression (ANTE) Normalization/harmonization methods: QN (A), DESeq2 (B), ComBat (C), Sh1 (D), Sh2PBR (E), Sh2QBR (F), Sh2RBR (G). H: legend for the sample types and batches. The ratio of sample-based to platform-based WM metrics is shown in the upper right corner of each panel.

***
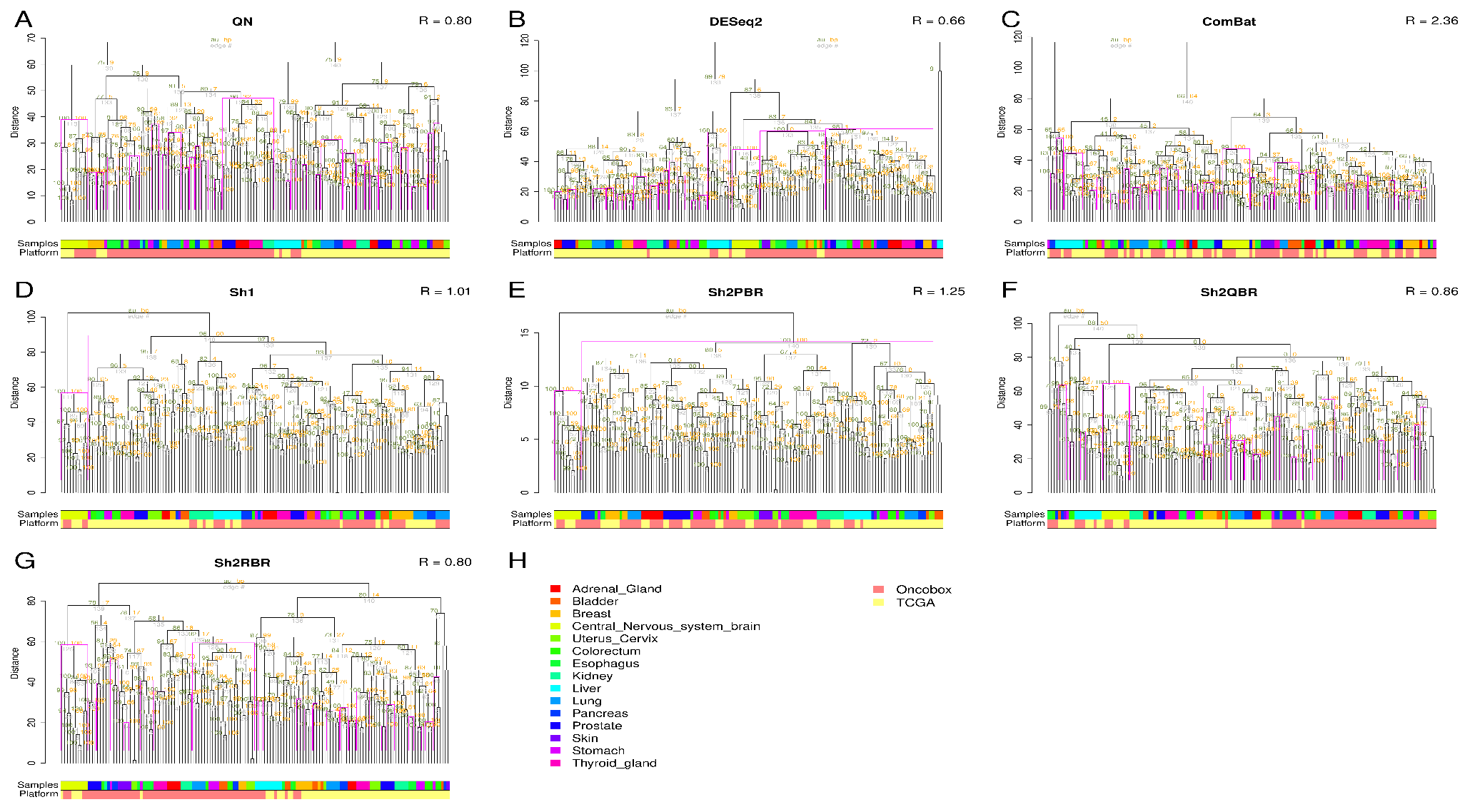
Supplementary Fig. 2-4.*** Clustering dendrograms for merged normal/control datasets TCGA (Tomczak et al., 2015), and Oncobox ANTE normal (Suntsova et al., 2019). Normalization/harmonization methods: QN (A), DESeq2 (B), ComBat (C), Sh1 (D), Sh2PBR (E), Sh2QBR (F), Sh2RBR (G). H: legend for the sample types and batches. The ratio of sample-based to platform-based WM metrics is shown in the upper right corner of each panel.

***
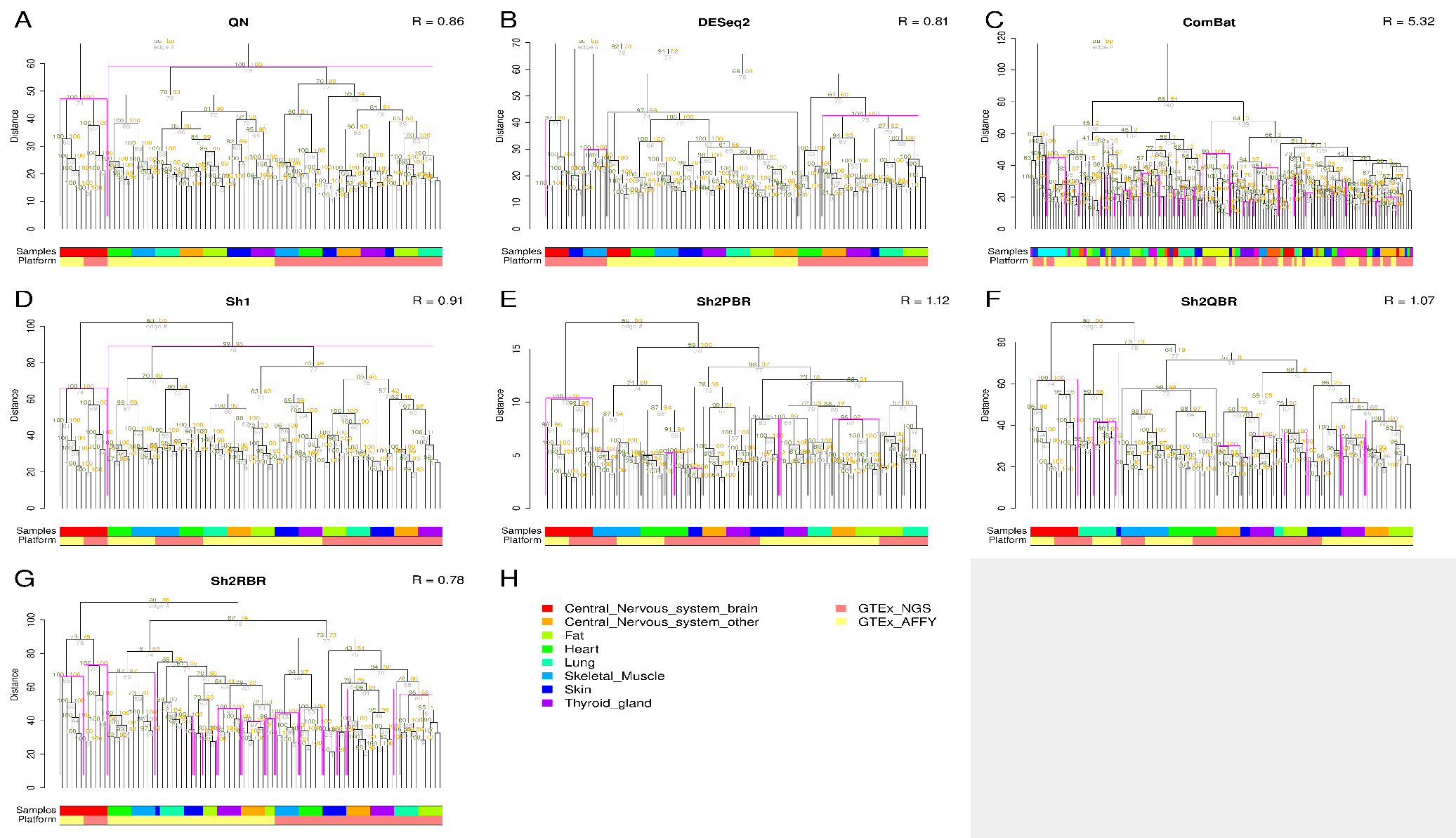
Supplementary Fig. 2-5.*** Clustering dendrograms for merged normal/control datasets GTEx NGS, and GTEx Affymetrix HUG1 (GTEx Consortium, 2013). Normalization/harmonization methods: QN (A), DESeq2 (B), ComBat (C), Sh1 (D), Sh2PBR (E), Sh2QBR (F), Sh2RBR (G). H: legend for the sample types and batches. The ratio of sample-based to platform-based WM metrics is shown in the upper right corner of each panel.

***
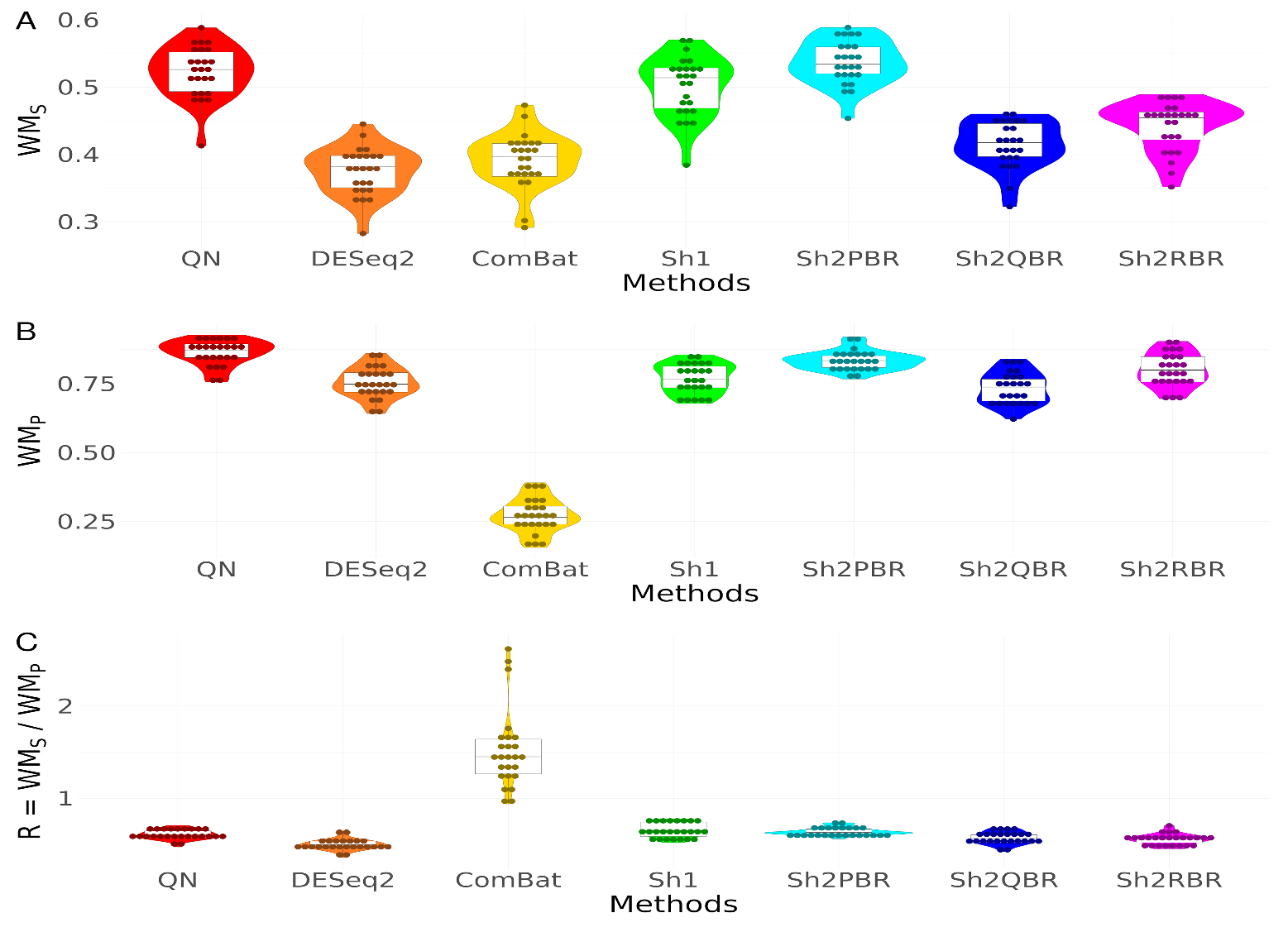
***

***Supplementary Fig. 2-6.*** WM-metrics ranking for different normalization/harmonization methods. (A) *WM_s_*; (B) *WM_p_*; (C) ratio, *R*, of sample type-based (*WM_s_*) to platform type-based (*WM_p_*) metrics for merged cancer datasets TCGA (Tomczak et al., 2015), and Oncobox (Borisov et al., 2022).

***
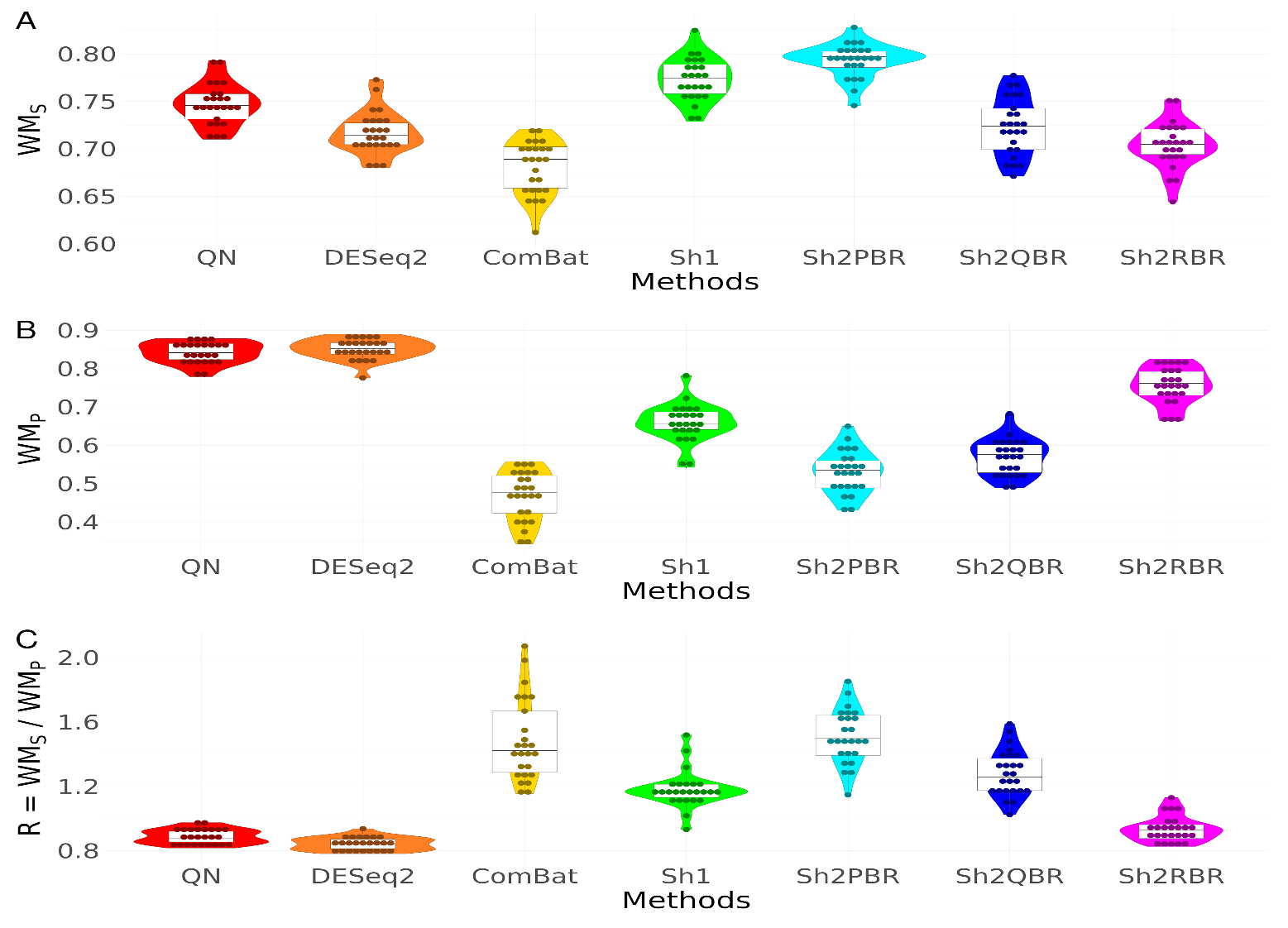
***

***Supplementary Fig. 2-7.*** WM-metrics ranking for different normalization/harmonization methods. (A) *WM_s_*; (B) *WM_p_*; (C) ratio, *R*, of sample type-based (*WM_s_*) to platform type-based (*WM_p_*) metrics for merged normal/control datasets GTEx NGS (GTEx Consortium, 2013), and TCGA (Tomczak et al., 2015).

***
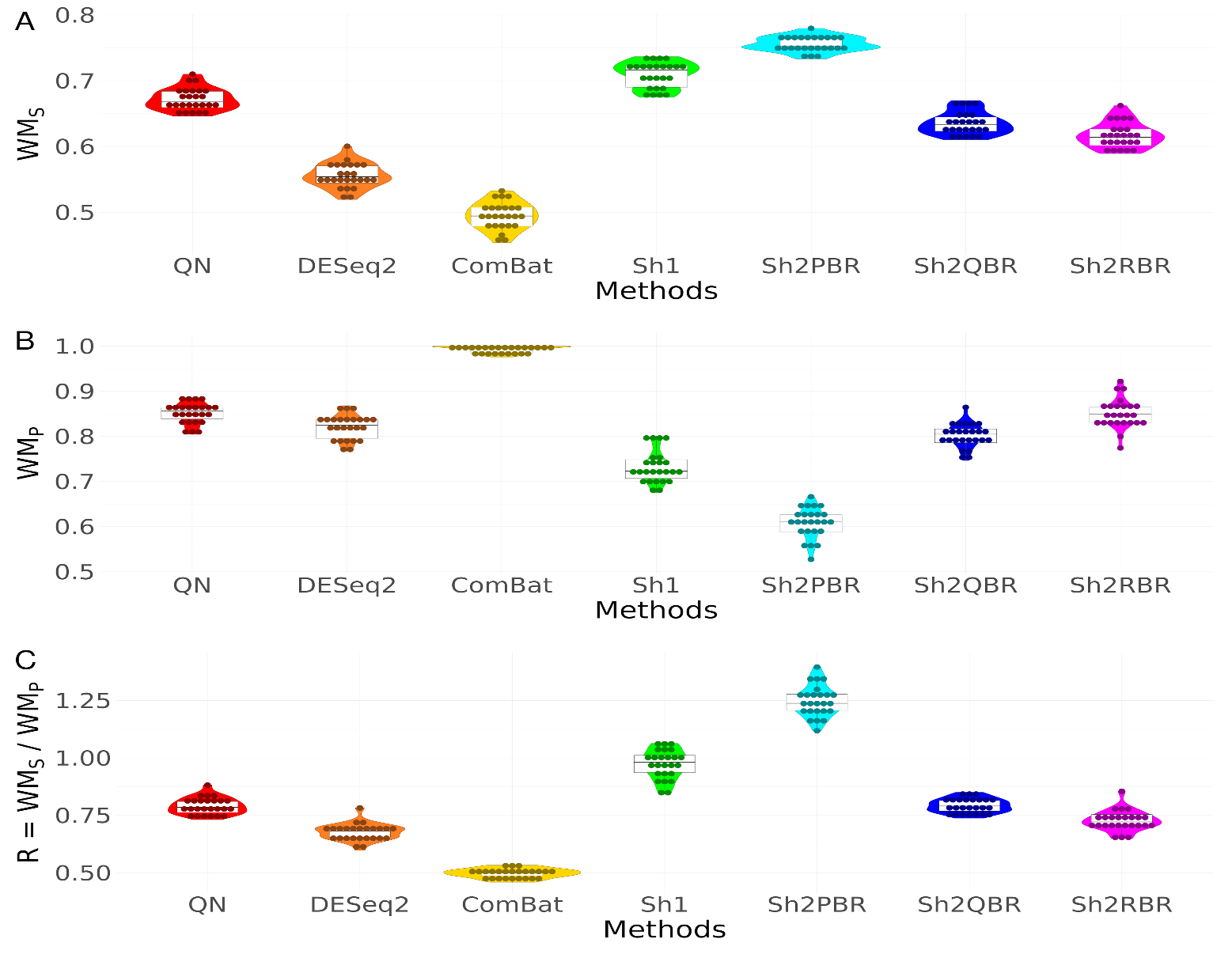
***

***Supplementary Fig. 2-8.*** WM-metrics ranking for different normalization/harmonization methods. (A) *WM_s_*; (B) *WM_p_*; (C) ratio, *R*, of sample type-based (*WM_s_*) to platform type-based (*WM_p_*) metrics for merged normal/control datasets GTEx NGS (GTEx Consortium, 2013), and Oncobox Atlas of Normal Tissue Expression (ANTE) (Suntsova et al., 2019).

***
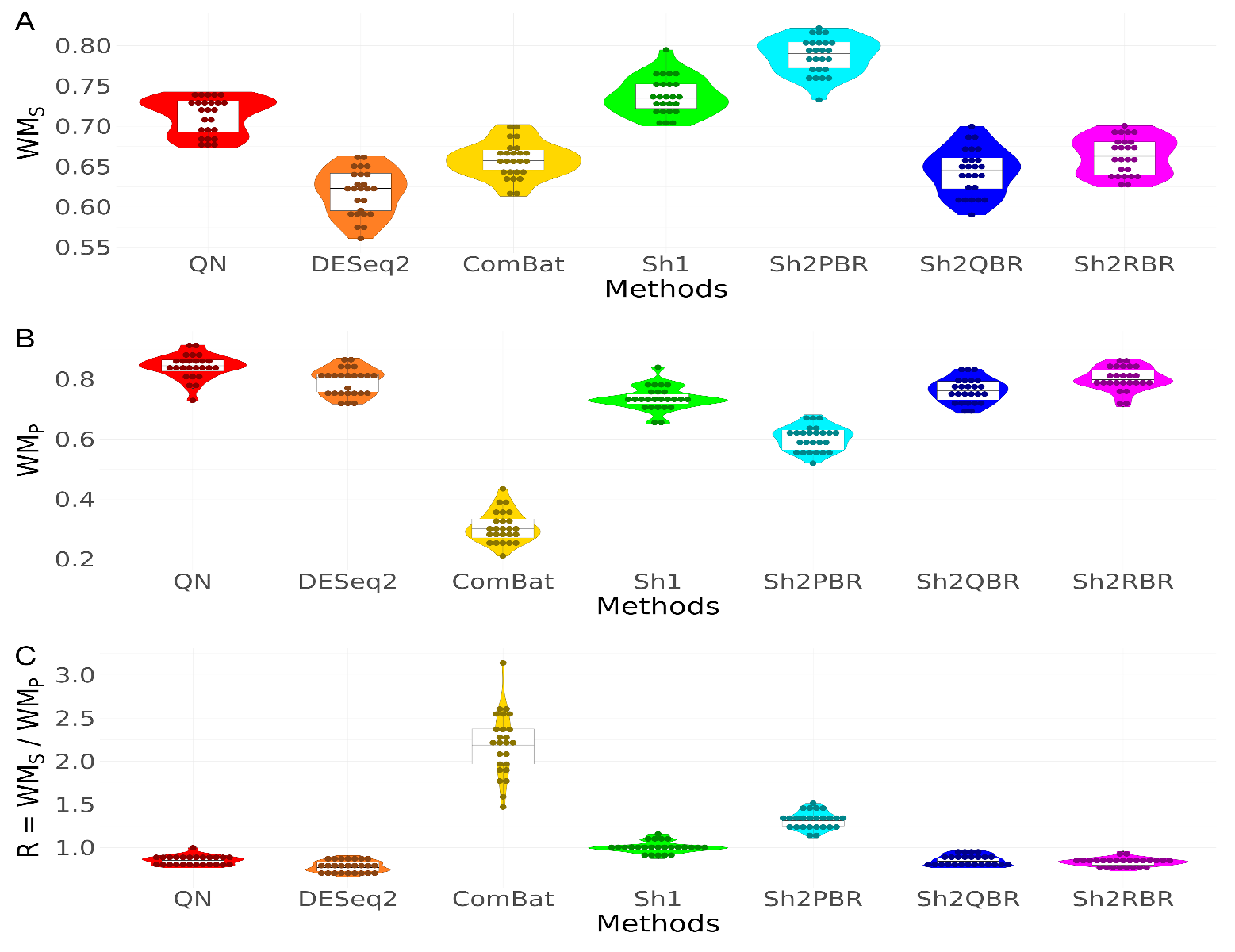
***

***Supplementary Fig. 2-9.*** WM-metrics ranking for different normalization/harmonization methods. (A) *WM_s_*; (B) *WM_p_*; (C) ratio, *R*, of sample type-based (*WM_s_*) to platform type-based (*WM_p_*) metrics for merged normal/control datasets TCGA (Tomczak et al., 2015) and Oncobox ANTE normal (Suntsova et al., 2019).

***
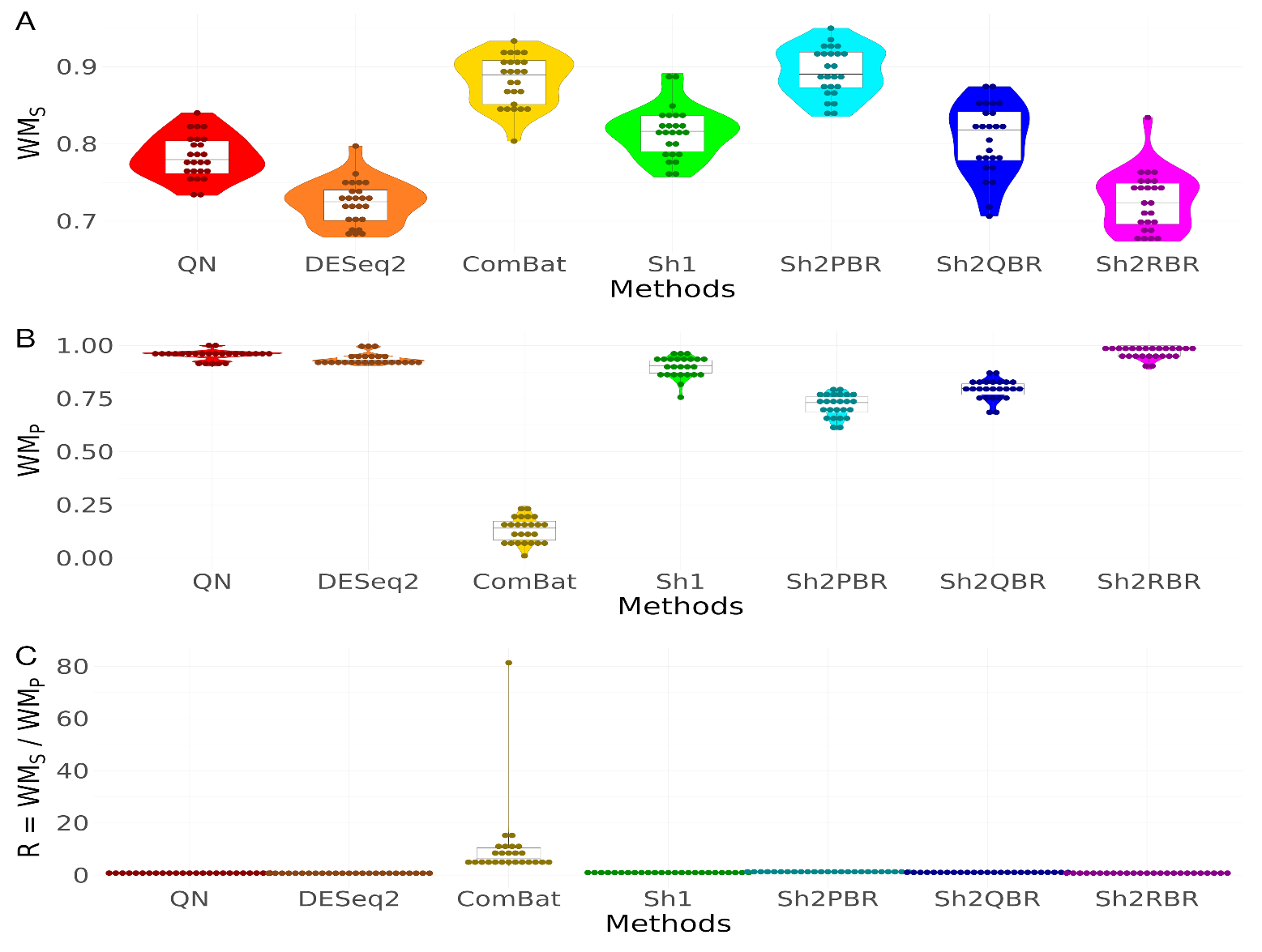
***

***Supplementary Fig. 2-10.*** WM-metrics ranking for different normalization/harmonization methods. (A) *WM_s_*; (B) *WM_p_*; (C) ratio, *R*, of sample type-based (*WM_s_*) to platform type-based (*WM_p_*) metrics for merged normal/control datasets GTEx NGS, and GTEx Affymetrix HUG1 (GTEx Consortium, 2013).

**References**

Borisov, N., Sorokin, M., Zolotovskaya, M., Borisov, C., and Buzdin, A. (2022). Shambhala‐2: A Protocol for Uniformly Shaped Harmonization of Gene Expression Profiles of Various Formats. *Current Protocols* 2. doi: 10.1002/cpz1.444.

GTEx Consortium (2013). The Genotype-Tissue Expression (GTEx) project. *Nature Genetics* 45, 580–585. doi: 10.1038/ng.2653.

Suntsova, M., Gaifullin, N., Allina, D., Reshetun, A., Li, X., Mendeleeva, L., et al. (2019). Atlas of RNA sequencing profiles for normal human tissues. *Scientific Data* 6. doi: 10.1038/s41597-019-0043-4.

Tomczak, K., Czerwinska, P., and Wiznerowicz, M. (2015). The Cancer Genome Atlas (TCGA): an immeasurable source of knowledge. *Contemporary Oncology (Poznan, Poland)* 19, A68–A77. doi: 10.5114/wo.2014.47136.
