# Supplementary material for: Uniformly shaped harmonization combines human transcriptomic data from different platforms while retaining their biological properties and differential gene expression patterns
Source: Front Mol Biosci. 2023 Sep 6;10:1237129. doi: 10.3389/fmolb.2023.1237129 (PMC10511763; doi:10.3389/fmolb.2023.1237129)
Supplement: Supplementary file 11 [file DataSheet1.docx]

Supplementary Material 1

Uniformly shaped harmonization combines human transcriptomic data from different platforms while retaining their biological properties and differential gene expression patterns

Nicolas Borisov, Victor Tkachev, Alexander Simonov, Maxim Sorokin, Ella Kim, Denis Kuzmin, Betul Karademir-Yilmaz, and Anton Buzdin

*** Correspondence:** Nicolas Borisov. [nicolasborissoff@gmail.com](mailto:nicolasborissoff@gmail.com)


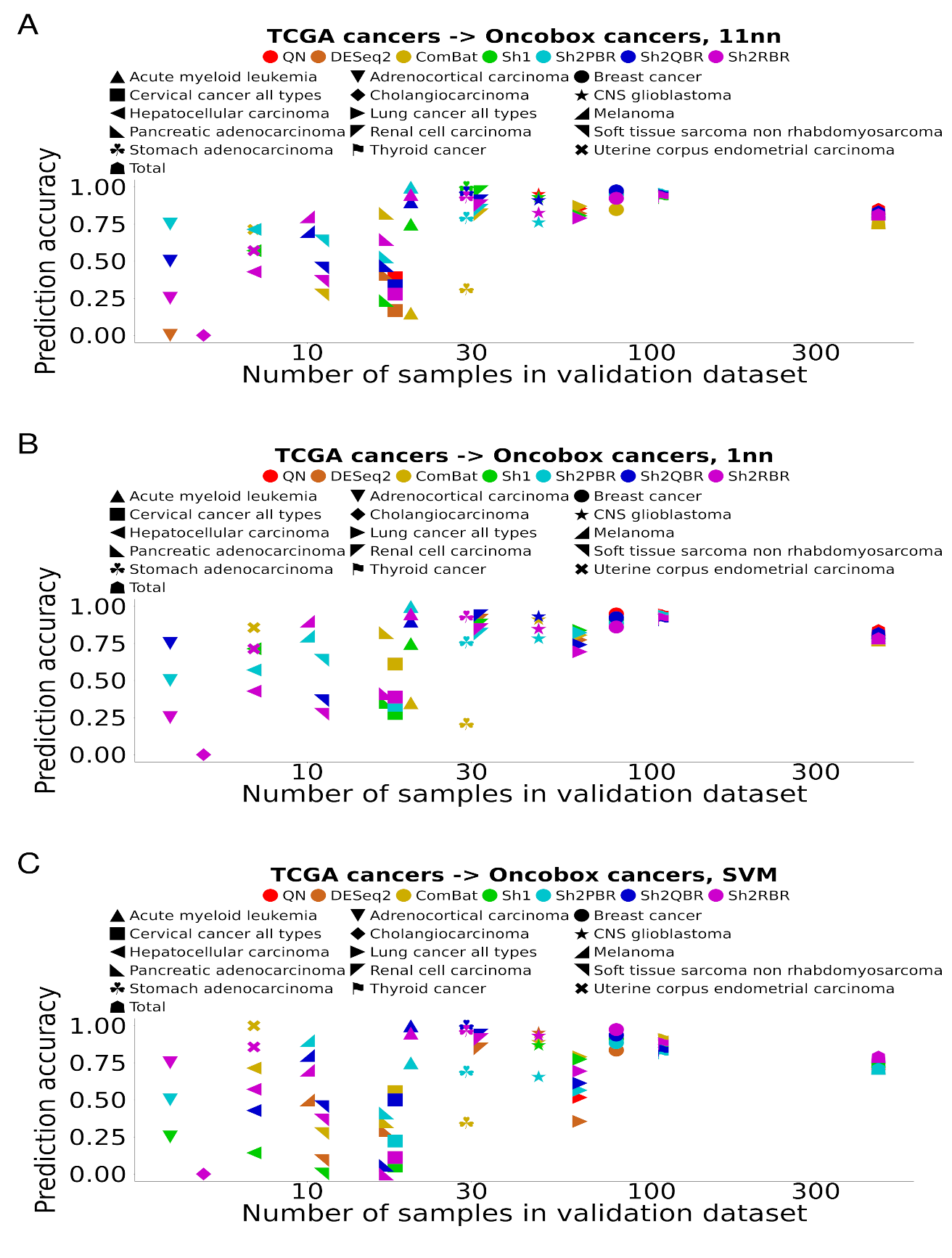


***Supplementary Fig. 1-1.*** Accuracy (i.e. rate of correct predictions for the given class) for a multi-class classifier based on the 11 nearest neighbors (A), 1^st^ nearest neighbor (B), and linear SVM (C). Training dataset: TCGA cancers (Tomczak et al., 2015). Validation dataset: Oncobox cancers (Borisov et al., 2022).

***
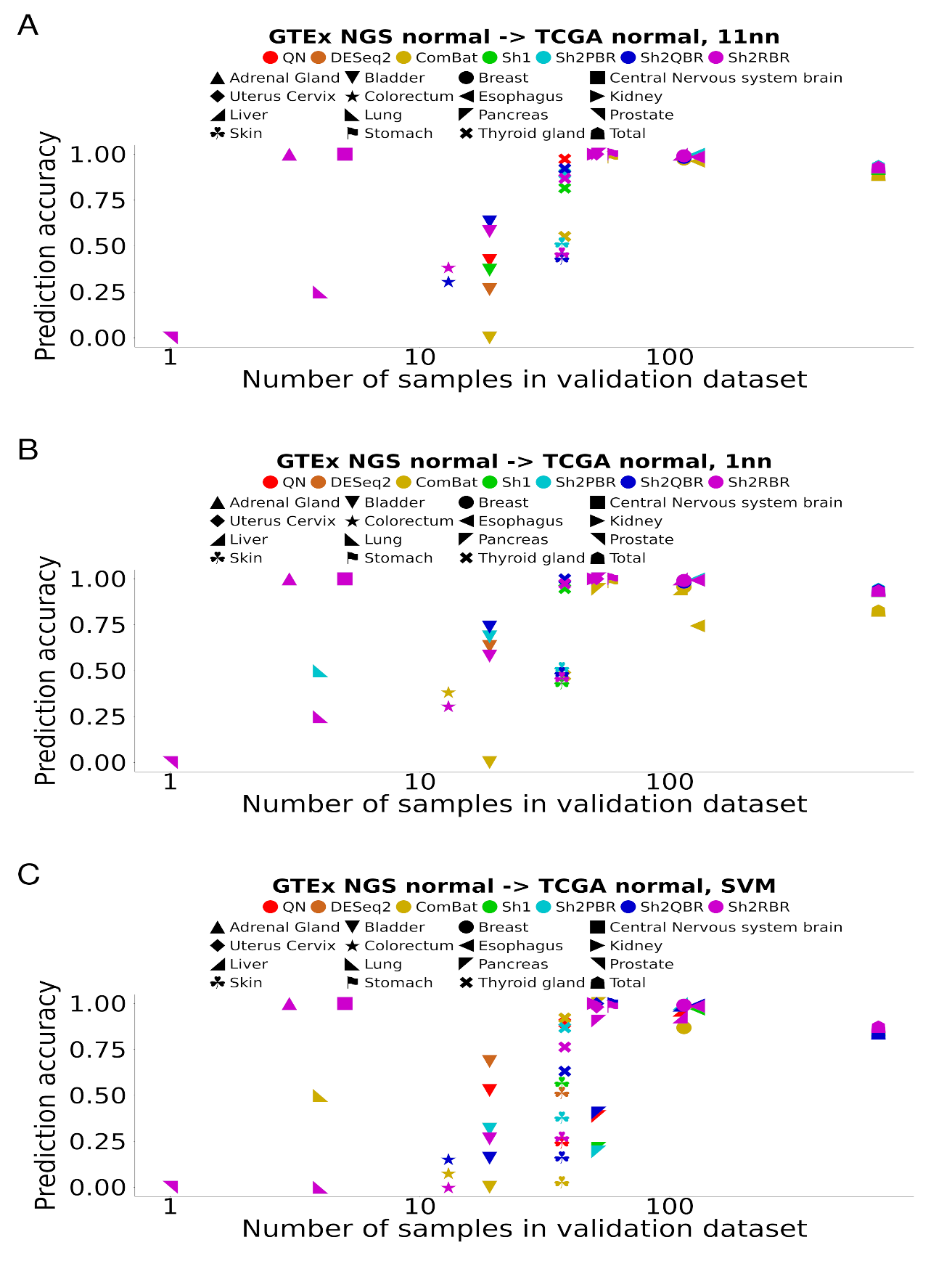
***

***Supplementary Fig. 1-2.*** Accuracy (i.e. rate of correct predictions for the given class) for a multi-class classifier based on the 11 nearest neighbors (A), 1^st^ nearest neighbor (B), and linear SVM (C). Training dataset: GTEx NGS normal (GTEx Consortium, 2013). Validation dataset: TCGA normal (Tomczak et al., 2015).

***
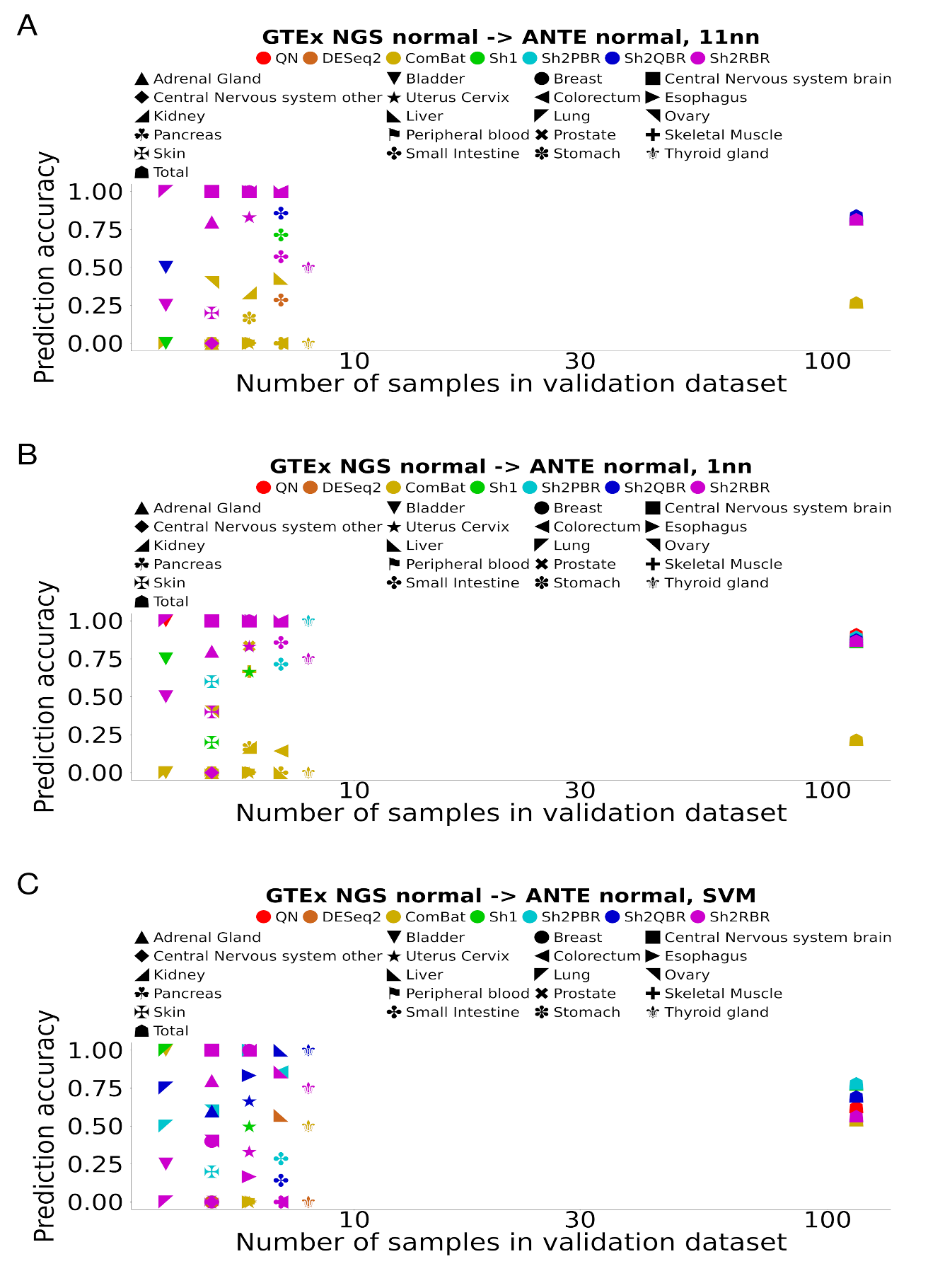
Supplementary Fig. 1-3.*** Accuracy (i.e. rate of correct predictions for the given class) for a multi-class classifier based on the 11 nearest neighbors (A), 1^st^ nearest neighbor (B), and linear SVM (C). Training dataset: GTEx NGS normal (GTEx Consortium, 2013). Validation dataset ANTE normal (Suntsova et al., 2019).

***
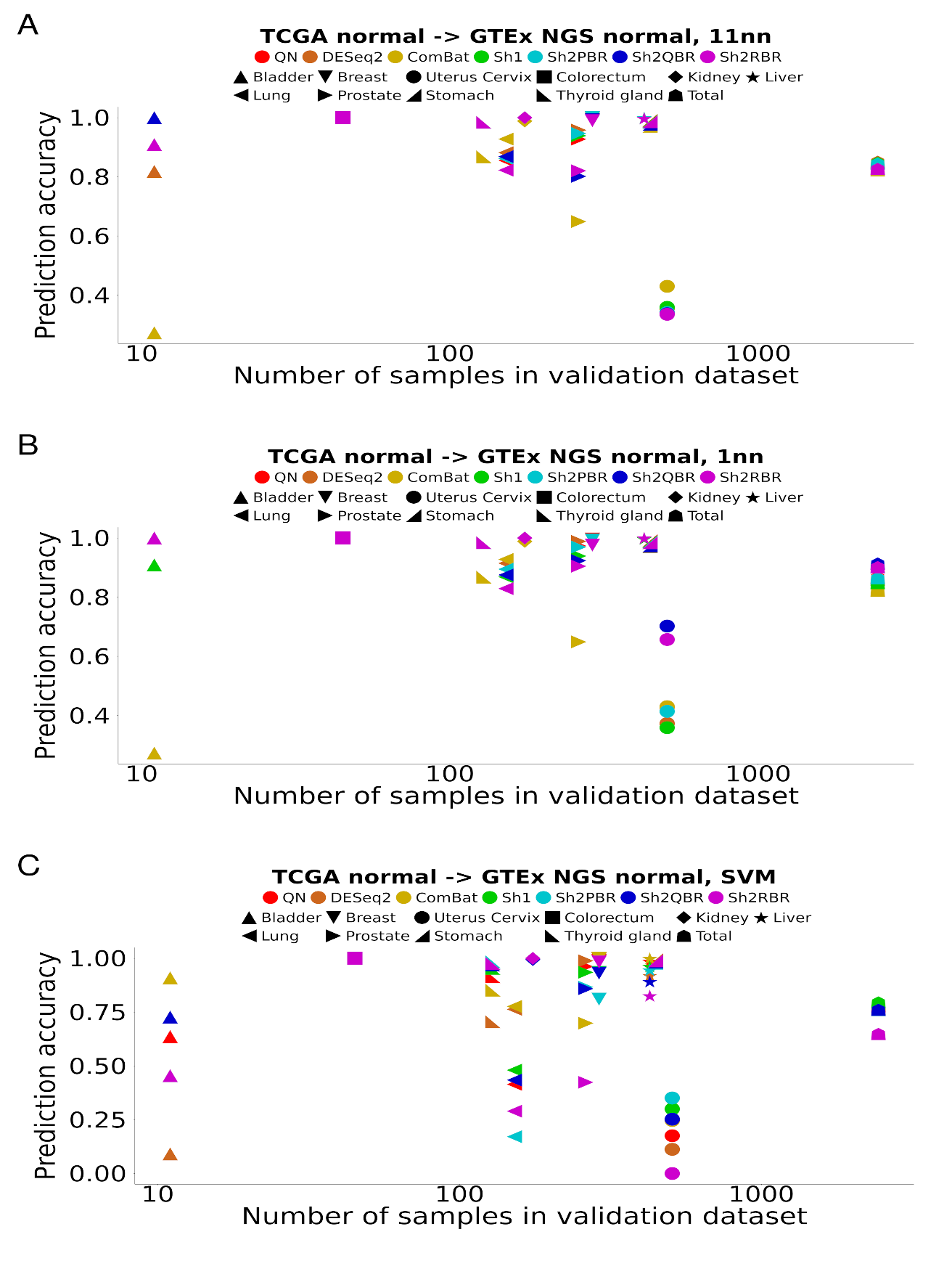
***

***Supplementary Fig. 1-4.*** Accuracy (i.e. rate of correct predictions for the given class) for a multi-class classifier based on the 11 nearest neighbors (A), 1^st^ nearest neighbor (B), and linear SVM (C). Training dataset: TCGA normal (Tomczak et al., 2015). Validation dataset: GTEx NGS normal (GTEx Consortium, 2013)..

***
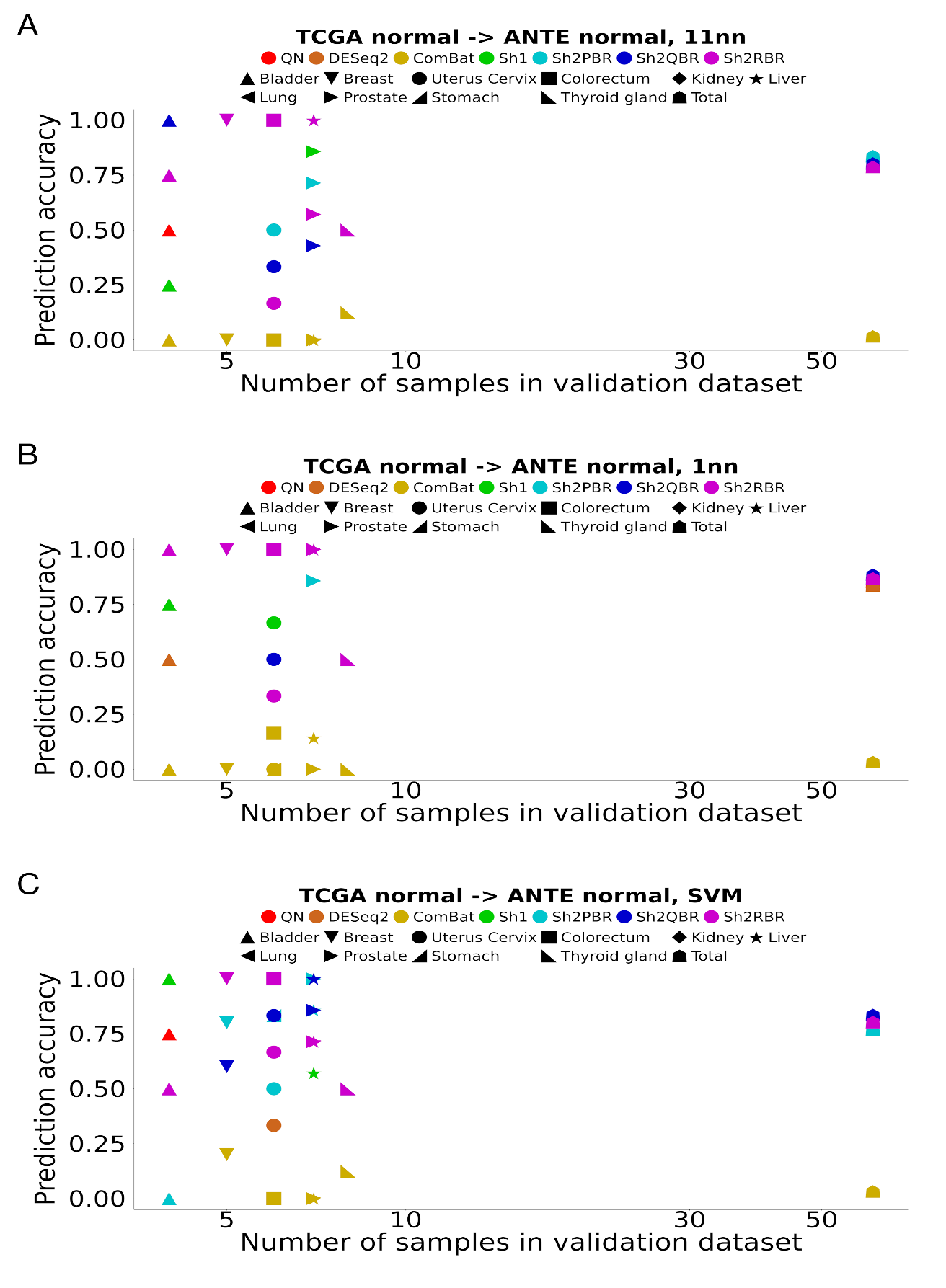
***

***Supplementary Fig. 1-5.*** Accuracy (i.e. rate of correct predictions for the given class) for a multi-class classifier based on the 11 nearest neighbors (A), 1^st^ nearest neighbor (B), and linear SVM (C). Training dataset: TCGA normal (Tomczak et al., 2015). Validation dataset: ANTE normal (Suntsova et al., 2019).


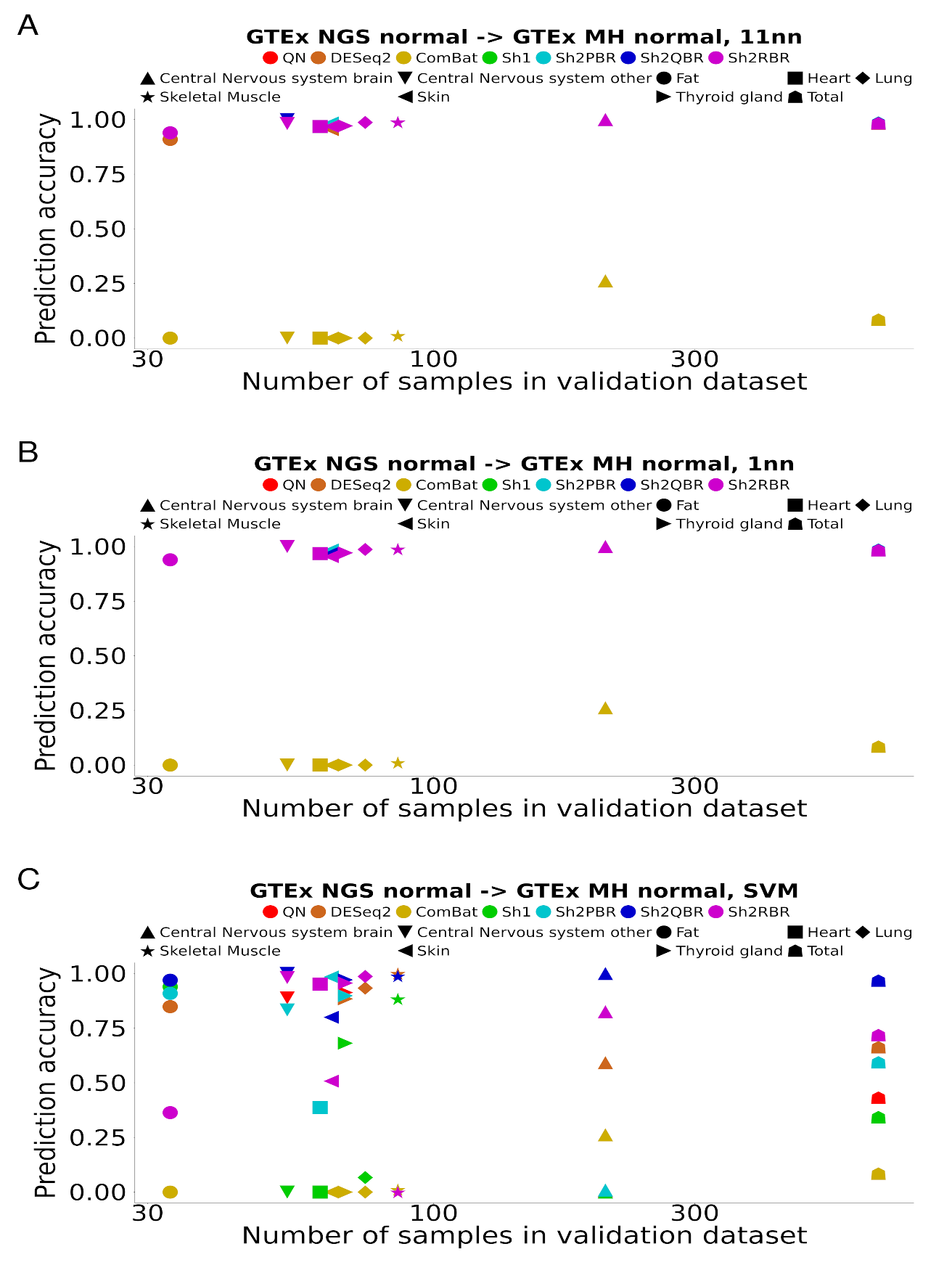


***Supplementary Fig. 1-6.*** Accuracy (i.e. rate of correct predictions for the given class) for a multi-class classifier based on the 11 nearest neighbors (A), 1^st^ nearest neighbor (B), and linear SVM (C). Training dataset: GTEx NGS normal (GTEx Consortium, 2013). Validation dataset: GTEx Affymetrix HUG1 normal (GTEx Consortium, 2013).


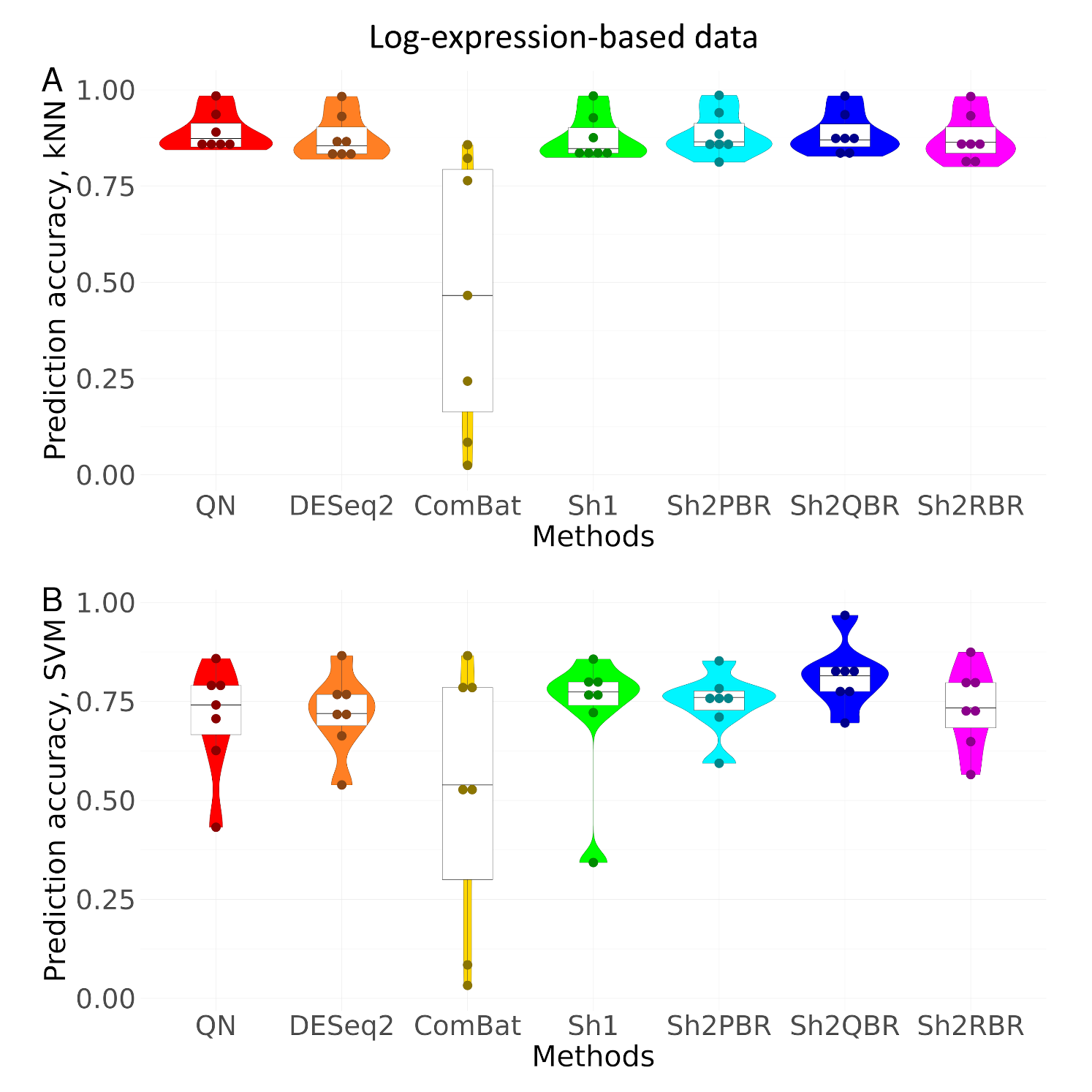


***Supplementary Fig. 1-7.*** Distribution of total tissue type prediction accuracies. A: calculated using local ML methods (averaged over the 11 nearest neighbor and the 1^st^ nearest neighbor methods). B: calculated using global (SVM) ML methods.

**References**

Borisov, N., Sorokin, M., Zolotovskaya, M., Borisov, C., and Buzdin, A. (2022). Shambhala‐2: A Protocol for Uniformly Shaped Harmonization of Gene Expression Profiles of Various Formats. *Current Protocols* 2. doi: 10.1002/cpz1.444.

GTEx Consortium (2013). The Genotype-Tissue Expression (GTEx) project. *Nature Genetics* 45, 580–585. doi: 10.1038/ng.2653.

Suntsova, M., Gaifullin, N., Allina, D., Reshetun, A., Li, X., Mendeleeva, L., et al. (2019). Atlas of RNA sequencing profiles for normal human tissues. *Scientific Data* 6. doi: 10.1038/s41597-019-0043-4.

Tomczak, K., Czerwinska, P., and Wiznerowicz, M. (2015). The Cancer Genome Atlas (TCGA): an immeasurable source of knowledge. *Contemporary Oncology (Poznan, Poland)* 19, A68–A77. doi: 10.5114/wo.2014.47136.
